# A Regularization-Based Adaptive Test for High-Dimensional Generalized Linear Models

**Published:** 2020-07-26

**Authors:** Chong Wu, Gongjun Xu, Xiaotong Shen, Wei Pan

**Affiliations:** Department of Statistics, Florida State University, FL, USA; Department of Statistics, University of Michigan, MI, USA; School of Statistics, University of Minnesota, MN, USA; Division of Biostatistics, University of Minnesota, MN, USA

**Keywords:** Adaptive Test, Truncated Lasso Penalty, Gene-Environmental Interaction

## Abstract

In spite of its urgent importance in the era of big data, testing high-dimensional parameters in generalized linear models (GLMs) in the presence of high-dimensional nuisance parameters has been largely under-studied, especially with regard to constructing powerful tests for general (and unknown) alternatives. Most existing tests are powerful only against certain alternatives and may yield incorrect Type I error rates under high-dimensional nuisance parameter situations. In this paper, we propose the adaptive interaction sum of powered score (aiSPU) test in the framework of penalized regression with a non-convex penalty, called truncated Lasso penalty (TLP), which can maintain correct Type I error rates while yielding high statistical power across a wide range of alternatives. To calculate its *p*-values analytically, we derive its asymptotic null distribution. Via simulations, its superior finite-sample performance is demonstrated over several representative existing methods. In addition, we apply it and other representative tests to an Alzheimer’s Disease Neuroimaging Initiative (ADNI) data set, detecting possible gene-gender interactions for Alzheimer’s disease. We also put R package “*aispu*” implementing the proposed test on GitHub.

## 1. Introduction

Statistical inference in high-dimensional models has been attracting increasing attentions, owing to the surge of high-dimensional data in many fields, such as in genetics and genomics. Accordingly, there is an increasing body of literature on significance testing in high-dimensional linear or generalized linear models (GLMs), mostly on low-dimensional regression coefficients. For example, Wasserman and Roeder (2009); Meinshausen et al. (2009) proposed random sample-splitting approaches to testing on a regression coefficient of interest in a high-dimensional model. Based on the idea of polyhedral selection, Lee et al. (2016) proposed an exact post-selection estimator conditional on the selection event. Meanwhile, many researchers exploit the idea of projection or bias-correction to handle the impact of regularization and high-dimensional nuisance parameters (e.g., Javanmard and Montanari, 2014; Van de Geer et al., 2014; Zhang and Zhang, 2014; Lee et al., 2016; Shi et al., 2019; Ma et al., 2020). In spite of exciting progresses in the last few years, little work has been done to construct more general and powerful tests on high-dimensional regression coefficients in GLMs in the presence of high-dimensional nuisance parameters.

It is noted that, for high-dimensional problems, classical or popular tests may not perform well, even if their asymptotic properties (such as their null distributions) are well established. Fan (1996) gave a simple example: given a *p*-dimensional vector follows a normal distribution, y ∼ *N* (*θ, I*), to test *H*_0_: *θ* = 0 versus *H*_*A*_: *θ* ≠ 0, the likelihood ratio test, Wald test, and score test statistics all share the same form $T={\left\|\text{y}\right\|}_{\text{2}}^{\text{2}}$, which is a sum of squares-type statistic; even under an alternative *H*_*A*_ with ${\left\|\theta \right\|}_{2}^{2}\to \infty$ as *p* → ∞, as long as ${\left\|\theta \right\|}_{2}^{2}=o\left(\sqrt{p}\right)$, the power of the three tests vanishes (i.e. tending to the Type I error rate); in contrast, some adaptive tests can be much more powerful. This example convincingly demonstrates the importance of considering the power of a test in high-dimensional settings and this article aims at filling this gap.

This work was motivated by a critical problem in genetics to identify interaction effects between a genetic marker set and a complex disease like Alzheimer’s. Although univariate single nucleotide polymorphism (SNP) based analyses for identifying gene-environment (*G* × *E*) interactions are popular in the community, relatively few of the findings have been replicated (Manuck and McCaffery, 2014). To improve statistical power and enhance results interpretation, many genetic association studies have now considered an alternate/supplementary approach to jointly test the interaction effect of all SNPs in a biological meaningful marker set, e.g., SNPs in a gene or a pathway (Lin et al., 2013, 2016; Su et al., 2017). Jointly testing the interaction effect of a marker set can be formulated as testing on a high-dimensional parameter (i.e., interactions between possibly high-dimensional genetic variants and environmental factors) in the presence of high-dimensional nuisance parameters (to adjust for the main effects of the genetic variants and other covariates) under a high-dimensional GLM. Since such interactions in human genetics have proven difficult to detect, while specific interaction patterns are largely unknown, it is critical to develop and apply more general and adaptive tests that are powerful across a wide range of unknown alternatives.

To account for impact of high-dimensional nuisance parameters in *G* × *E* interactions testing problems, some variance-component score tests with the sum of squares-type statistics, coupled with the ridge regression to estimate the nuisance parameters under the null, have been proposed (Lin et al., 2013, 2016; Su et al., 2017). For example, Lin et al. (2013) proposed a test called gene-environment set association test (GESAT) by assuming that the *G* × *E* interaction effects follow an unspecified distribution with mean 0 and variance *υ*^2^, then testing *H*_0_ : *υ*^2^ = 0 for the overall *G* × *E* interaction. By noting that the ridge estimator is $\sqrt{n}$-consistent under suitable conditions (Knight and Fu, 2000), they derived the theoretical null distribution for GESAT. While enticing, the $\sqrt{n}$-consistency of the ridge estimator or asymptotic normality of the score vector may not be applicable under high-dimensional situations with finite samples, leading to incorrect Type I error rates. As to be shown in simulations, as the number of the covariates increases, methods based on the ridge penalty yield incorrect Type I error rates and substantial power loss.

Meanwhile, bias-correction based methods have been proposed (Dezeure et al., 2017; Zhang and Cheng, 2017). For example, inspired by the desparsifying Lasso estimator (Van de Geer et al., 2014) and the data-splitting strategy (Wasserman and Roeder, 2009), Zhang and Cheng (2017) proposed a three-step bootstrap-assisted procedure based on a supremum-type statistic to test on high-dimensional regression coefficients in high dimensional regression models. This method can control the Type I error rate well and yield high statistical power under highly sparse alternatives. However, due to the accumulation of estimation errors of the desparsifying Lasso estimator, the estimation errors might be out of control if a burden-type or sum of squares-type statistic is used (Zhang and Cheng, 2017). Moreover, although the data-splitting strategy adopted therein helps control the Type I error rate, it reduces the power as well.

To address those challenges, we propose an adaptive test, referred to as adaptive interaction sum of powered score (aiSPU) test, for testing high-dimensional regression coefficients under GLMs with high-dimensional nuisance parameters. The aiSPU test is new and appealing in two aspects. First, in aiSPU we apply the truncated Lasso penalty (TLP) (Shen et al., 2012), a non-convex penalty, to estimate the high-dimensional nuisance parameter under the null hypothesis. The TLP estimator consistently reconstructs the oracle estimator under mild assumptions, helping maintain correct Type I error rates under a high-dimensional situation. In contrast, the consistency of a convex penalty-based estimator, such as the ridge or Lasso estimator, holds under much stronger conditions. For example, the Lasso estimator is consistent under a strong irrepresentable (Wainwright, 2009), while the ridge estimator is consistent under the assumption that the sample covariance matrix of all the covariates converges to a non-singular matrix (Knight and Fu, 2000). Second, because the true alternative hypothesis is generally complex and unknown, we apply the idea of an adaptive testing (Pan et al., 2014). We first construct a group of interaction sum of powered score (iSPU) tests such that hopefully at least one of them would be powerful for a given alternative. The proposed adaptive test then data-adaptively selects the one with the most significant result with a proper adjustment for multiple testing to control the Type I error rate, and thus achieves high power.

To apply the proposed test, we establish its asymptotic null distribution. In particular, we derive the joint asymptotic distribution for a set of the iSPU tests. The marginal distribution of each iSPU test statistic converges to either a normal distribution or an extreme value distribution under some conditions. Based on the theoretical results, we develop an asymptotic way to calculate the *p*-values for the iSPU and aiSPU simultaneously. We demonstrate the superior performance of the proposed test with some theoretical power analyses under local alternatives. Further, as to be shown in simulations and real data analyses, the proposed aiSPU test would yield correct Type I error rates and higher statistical power than several existing methods under a wide range of high-dimensional alternative hypotheses, ranging from highly dense to highly sparse alternatives.

The rest of the paper is organized as follows. In Section 2, we review two representative tests before proposing our new aiSPU test. Results for simulations and analysis of the Alzheimer’s Disease Neuroimaging Initiative (ADNI) data are presented in Sections 3 and 4, respectively. We conclude with a short discussion in Section 5. All technical details, proofs, and extensive simulation results are relegated to Appendices. We have released open source R package *aispu* implementing the proposed test on GitHub (https://github.com/ChongWu-Biostat/aispu), and will upload it to CRAN soon.

## 2. Methods

### 2.1. Notation and model

Even though our study was motivated by detecting gene-environmental interactions, our proposed method is general and applicable to many other problems, thus we introduce our method in a general framework. Suppose we have *n* independent and identically distributed (IID) observations $\left\{\left(Y_{i},Z_{i},X_{i}\right):i=1,2,\ldots ,n\right\}$, for which we denote an *n*-vector outcome (response)$Y={\left(Y_{1},\ldots ,Y_{n}\right)}^{\prime }$, an *n* × *q* matrix $\mathbb{Z}=Z^{\prime }{\left(Z_{1}^{'},\ldots ,Z_{n}^{'}\right)}^{\prime }$ for *q* nuisance covariates (including the intercept term) with $Z_{i}=\left(Z_{i1},\ldots ,Z_{iq}\right)$, and an *n*×*p* matrix $X={\left(X_{1}^{'},..,X_{n}^{'}\right)}^{\prime }$ for *p* variables of interest with $X_{i}=\left(X_{i1},\ldots ,X_{ip}\right)$. Without loss of generality, we assume that *E*(*X*_*i*_) = 0 as otherwise each *X*_*i*_ can be re-centered by its sample mean. We consider a generalized linear model with the canonical link function,

(1) $$E\left(Y|X,\mathbb{Z}\right)=g^{-1}\left(X\beta +\mathbb{Z}\vartheta \right),$$

where *p*-vector *β* and *q*-vector *ϑ* are unknown parameters, and *g* is the canonical link function. We are interested in testing

(2) $$H_{0}:\beta =0\;\;\;\text{versus}\;\;H_{1}:\beta \neq 0,$$

while treating *ϑ* as the high-dimensional nuisance parameter. We target the situation with “large *q* and *p*.”

**Remark 1** Numerous real-world problems can be formulated as testing high-dimensional parameters under GLMs in the presence of high-dimensional nuisance parameters. For example, when testing the interaction between a genetic marker set and a set of environmental variables, we can let ℤ be the environmental factors, genotypes in the marker-set, and some important covariates, and let X be the SNP-environment interaction variables. Here we consider a large number of SNPs in a marker-set, leading to high-dimensional q and p. Another example is testing gene-gene interactions (Cordell, 2009), a problem can be formulated with ℤ being all the SNPs from two genes and X being their interactions.

### 2.2. Related existing methods

In this subsection, we review two representative methods: a variance component type test called GESAT (Lin et al., 2013) and a bias-correction based test (Zhang and Cheng, 2017).

By assuming *β*_*j*_’s follow an arbitrary distribution with mean zero and variance *υ*^2^, GESAT converts testing *H*_0_ : *β* = 0 to testing $H_{0}^{\prime }:v^{2}=0$, which can be conducted via the following sum of squares-type statistic:$Q={\left(Y-\mu \left(\hat{\vartheta }\right)\right)}^{\prime }XX^{\prime }\left(Y-\mu \left(\hat{\vartheta }\right)\right)$, where $\mu \left(\hat{\vartheta }\right)=g^{-1}\left(\mathbb{Z}\hat{\vartheta }\right)$ and $\hat{\vartheta }$ is estimated under the null model,

(3) $$E\left(Y|\mathbb{Z}\right)=g^{-1}\left(\mathbb{Z}\vartheta \right).$$

To account for high-dimensionality of *ϑ*, GESAT applies the ridge regression to estimate *ϑ* under the null model (3). Using the property that the ridge estimator $\hat{\vartheta }$ is $\sqrt{n}$-consistent under suitable conditions (Knight and Fu, 2000), they showed that test statistic *Q* asymptotically follows a mixture of *χ*^2^ distributions under the *H*_0_, thus *p*-values can be calculated accordingly (Lin et al., 2013).

Meanwhile, a three-step bootstrap-assisted procedure (Zhang and Cheng, 2017) has been proposed to test on *H*_0_. First, it randomly splits the sample into two sub-samples. Second, it screens out the irrelevant variables of X based on the first sub-sample. After screening, denote the reduced model S={j:j∉{irrelevant variables}}. Third, it computes the desparsifying Lasso estimator ${\left\{{\overset{\vee }{\beta }}_{j}\right\}}_{j\in S}$ and the corresponding variance estimator ${\overset{\vee }{w}}_{jj}$ based on the second sub-sample. The non-studentized (NST) and studentized (ST) supremum type statistic are $\max_{j\in S}\sqrt{n}\left|\overset{\vee }{\beta }\right|$ and $\max_{j\in S}\sqrt{n}\left|{\overset{\vee }{\beta }}_{j}\right|/\sqrt{{\overset{\vee }{w}}_{jj}}$, respectively. Zhang and Cheng (2017) then applied a bootstrap-assisted procedure to calculate their *p*-values.

Though appealing, both tests have limitations. First, a test based on the ridge penalty (such as GESAT) might yield incorrect Type I error rates when the dimensionality of nuisance parameters *ϑ* (i.e., *q*) is high. Note that the null distribution of GESAT is derived based on the $\sqrt{n}$-consistent ridge estimator, which requires that the sample covariance matrix of *Z*_*i*_ converges to a non-singular matrix (Knight and Fu, 2000); this assumption will not hold when *q > n*. This may explain incorrect Type I error rates of GESAT as to be shown in simulations. Second, the existing tests might be powerless under some alternatives. It is well known that a sum of squares-type statistic (for example, GESAT) and a supremum-type statistic (for example, NST and ST) are more powerful for dense and highly sparse nonzero signals, respectively (Pan et al., 2014). However, for moderately dense nonzero signals, neither may be powerful: there might not exist one or few components of *β* to represent a strong departure from *H*_0_, whereas a sum of squares statistic might accumulate too much noises or estimation errors through summing over the non-informative components. Furthermore, both NST and ST only use a sub-sample to construct test statistics, further reducing power. As to be shown in simulations, the above methods would lose substantial power under some alternatives.

### 2.3. New test statistics

There are two main challenges for constructing a powerful test in a high-dimensional setting. First, estimating the high-dimensional *ϑ* under *H*_0_ is not trivial. Second, because the underlying association patterns are unknown, it is crucial to construct an adaptive test such that it can maintain high power across a wide range of alternatives.

To accurately estimate the high-dimensional *ϑ* under the null model (3), we apply penalized regression by imposing the truncated Lasso penalty (TLP) (Shen et al., 2012) on the nuisance parameter *ϑ*. For gene-environmental interaction problems, we can impose no penalty on a subset of some pre-specified low-dimensional covariates to keep some important covariates, such as age and gender. TLP is defined as TLP(x,τ)=min(|x|,τ) for a scalar *x* and a tuning parameter *τ*. It can be regarded as the Lasso penalty for a small |x|≤τ, but imposes no penalty for a large |x|>τ. We use 10-fold cross-validation to select the tuning parameters for TLP and denote $\hat{\vartheta }$ as the TLP estimate of *ϑ* under *H*_0_.

To maintain high power across various alternatives, we construct an adaptive test. Up to some constant, the score vector $U={\left(U_{1},\ldots ,U_{p}\right)}^{\prime }$ for *β* in (1) is $U_{j}=\frac{1}{n}\sum_{i=1}^{n}\left(Y_{i}-{\hat{\mu }}_{0i}\right)X_{ij}$ for 1 ≤ *j* ≤ *p*, where ${\hat{\mu }}_{0i}=g^{-1}\left(Z_{i}\hat{\vartheta }\right)$. Denote

$$U_{ij}=\left(Y_{i}-{\hat{\mu }}_{0i}\right)X_{ij}$$

for 1 ≤ *i* ≤ *n* and 1 ≤ *j* ≤ *p*. We first propose a class of test statistics called interaction sum of powered score (iSPU) with power index *γ >* 0 as

$$L\left(\gamma \right)=\sum_{j=1}^{p}{\left(\frac{1}{n}\sum_{i=1}^{n}U_{ij}\right)}^{\gamma }.$$

Since $L{\left(\gamma \right)}^{1/\gamma }\to \max_{1\leq i\leq p}\left|\frac{1}{n}\sum_{i=1}^{n}U_{ij}\right|$ as even integer *γ* → ∞, we define *L*(∞) as

$$L\left(\infty \right)=\underset{1\leq j\leq p}{\max}\frac{n{\left(\frac{1}{n}\sum_{i=1}^{n}U_{ij}\right)}^{2}}{{\overset{\vee }{\sigma }}_{jj}},$$

where $\overset{\vee }{\text{Σ}}={\left({\overset{\vee }{\sigma }}_{kj}\right)}_{p\times p}$ is the covariance matrix with ${\overset{\vee }{\sigma }}_{kj}=\mathrm{cov}\left[U_{1k},U_{1j}\right]$ for 1 ≤ *k, j* ≤ *p*.

As to be shown in simulations, the power of *L*(*γ*) depends on the unknown *β* under specific alternatives. Since in general there is no uniformly most-powerful test, to maintain high power across various alternatives, we propose the adaptive interaction sum of powered score (aiSPU) to combine the multiple iSPU tests with different *γ*:

$$T_{\text{aiSPU}}=\underset{\gamma \in \Gamma }{\min}P_{L\left(\gamma \right)},$$

where $P_{L\left(\gamma \right)}$ is the *p*-value for *L*(*γ*) and Γ contains the candidate values of *γ*, e.g., Γ={1,2,…,6,∞}. We take the minimum *p*-value to approximately select the most powerful candidate test; *T*_aiSPU_ is the test statistic, but no longer a genuine *p*-value.

To emphasize the penalty we use, in some places, we denote iSPU(*γ*) and aiSPU explicitly with the penalty, say TLP, as iSPU(TLP,*γ*) and aiSPU(TLP), respectively.

**Remark 2** Accurate estimation of ϑ under the null is crucial in the situation with a high-dimensional nuisance parameter. Because the $\sqrt{n}$-consistency of the ridge estimator may not hold under a (relatively) high-dimensional situation, a test coupled with ridge regression may yield incorrect Type I error rates. The estimation errors of the desparsifying Lasso estimator might be out of control if a burden-type or sum of squares-type statistic is used (Zhang and Cheng, 2017), while the three-step bootstrap-assisted procedure based on a supremum-type statistic will not be powerful under dense alternatives. In contrast, because TLP enjoys the selection consistency and optimal parameter estimation under some mild conditions (Shen et al., 2012), aiSPU controls Type I error rates and achieves high power under a wide range of high-dimensional situations.

**Remark 3** The proposed aiSPU test can be viewed as an extension of the aSPU test (Wu et al., 2019) to high-dimensional nuisance parameter situations. The aSPU test was proposed for the situations with large p but small q, while the aiSPU test targets situations with large p and large q. Thus, the Type I error rate can be controlled by aiSPU, but not by aSPU in high-dimensional nuisance parameter situations (large q).

**Remark 4** Our proposed test may share some limitations of the standard score test with possible loss of power under H_A_, which can be fixed by taking an approach as shown in Wang (2016).

### 2.4. Asymptotic null distribution

In this subsection, we derive the asymptotic null distribution for iSPU. Before stating the theorem, we define necessary notation as follows. Let $\mu_{0}\equiv {\left(\mu_{01},\ldots ,\mu_{0n}\right)}^{\prime }$ be the conditional mean of *Y* under *H*_0_, where $\mu_{0i}=E\left(Y_{i}|H_{0}\right)=E\left(Y_{i}|Z_{i}\right)=g^{-1}\left(Z_{i}\vartheta^{0}\right)$ and *ϑ*^0^ is the population value of *ϑ*. Write $S_{ij}=\left(Y_{i}-\mu_{0i}\right)X_{ij}$ for 1 ≤ *i* ≤ *n* and 1 ≤ *j* ≤ *p*. We further define the corresponding covariance matrix $\text{Σ}={\left(\sigma_{kj}\right)}_{p\times p}$ with $\sigma_{kj}=\text{Cov}\left[S_{1k},S_{1j}\right]$ for 1 ≤ *k, j* ≤ *p*. For simplicity, we denote $L\left(\gamma ,\mu_{0}\right)=\sum_{j=1}^{p}L^{\left(j\right)}\left(\gamma ,\mu_{0}\right)$ with $L^{\left(j\right)}\left(\gamma ,\mu_{0}\right)={\left(\frac{1}{n}\sum_{i=1}^{n}S_{ij}\right)}^{\gamma }$ for 1 ≤ *i* ≤ *n* and 1 ≤ *j* ≤ *p*. Then the mean and variance of *L*(*γ, µ*_0_) can be denoted by $\psi \left(\gamma \right)=\sum_{j=1}^{p}\psi^{\left(j\right)}\left(\gamma \right)$ with $\psi^{\left(j\right)}\left(\gamma \right)=E\left[L^{\left(j\right)}\left(\gamma ,\mu_{0}\right)|H_{0}\right]$, and by $\omega^{2}\left(\gamma \right)=\mathrm{var}\left[L\left(\gamma ,\mu_{0}\right)|H_{0}\right]$, respectively.

Theorem 1 shows that (1) each iSPU(*γ*) converges to either a normal distribution or an extreme value distribution; (2) iSPU(∞) and iSPU with a finite *γ* are asymptotically independent under the *H*_0_.

Theorem 1. Under assumptions C1–C7 stated in Appendix A and under the null hypothesis

H_0_, for any fixed and finite Γ set we have:

- For finite candidate values γ in Γ, that is, $\Gamma^{\prime }=\Gamma \backslash \left\{\infty \right\}$, the vector of the iSPU test statistics ${\left[\left\{L\left(\gamma \right)-\psi \left(\gamma \right)\right\}/\omega \left(\gamma \right)\right]}_{\gamma \in \Gamma^{\prime }}^{'}$ converges weakly to a normal distribution with mean 0 and covariance matrix $\text{R}\left(\Gamma^{\prime }\right)=\left(\rho_{st}\right)$, i.e., $N\left(0,\text{R}\left(\Gamma^{\prime }\right)\right)$ as n, p → ∞, where ψ(γ), ω(γ), and $\text{R}\left(\Gamma^{\prime }\right)$ are defined in Appendix B.
- For γ = ∞, let a_p_ = 2 log p − log log p, for any real number x, $Pr\left\{L\left(\infty \right)-a_{p}\leq x\right\}\to \exp\left\{-\pi^{-1/2}\exp\left(-x/2\right)\right\}$.
- ${\left[\left\{L\left(\gamma \right)-\psi \left(\gamma \right)\right\}/\omega \left(\gamma \right)\right]}_{\gamma \in \Gamma^{\prime }}^{'}$ is asymptotically independent of L(∞), that is, the joint distribution of $\left[{\left\{L\left(\gamma \right)-\psi \left(\gamma \right)\right\}/\omega \left(\gamma \right)}_{\gamma \in \Gamma^{\prime }}^{'}\right]$ and L(∞) − a_p_ converges weakly to the product of the limiting distributions given in (i) and (ii).

**Remark 5** We leave the technical details and assumptions into the Appendices A–C. Intuitively speaking, L(γ, µ_0_) with a finite γ follows a normal distribution asymptotically when S_ij_1 and S_ij_2 are independent for j_1_ ≠ j_2_. Under moment assumptions that put constraints on correlation structures, we prove that the asymptotically normal still holds for L(γ) with a finite γ and a TLP-based estimate $\hat{\vartheta }$. For L(∞), we derive its distribution based on theorems in Cai et al. (2014). Of note, Wu et al. (2019) derived a similar Theorem under a much simpler context; our current proof is different and more challenging due to the technical complications under the adopted penalized regression framework to deal with the presence of high-dimensional nuisance parameters.

**Remark 6** In Theorem 1, we assume technical assumptions, such as a sparsity assumption regarding the effect from ℤ (C3) and a feature selection assumption involving Hellinger distance (C7). Because C7 is hard to validate in practice, we propose a stronger than needed beta-min like condition C7*, which is a sufficient assumption for C7. As to be shown in simulations, when the effect from ℤ is non-sparse but with sparse strong signals, our proposed method still works. In other words, under situations where C3 and C7* have been violated but C7 might hold, our proposed method still works.

These technical assumptions are used to establish the difference between µ_0_ and ${\hat{\mu }}_{0}$ is a small order term, which can be ignored in the theoretical derivation. Once we have a good estimate of the conditional mean of Y under H_0_ (i.e., µ_0_), our proposed method works. In principle, our proposed aiSPU test can be extended to consider higher-order interactions within ℤ if µ_0_ can be well estimated. We leave this interesting topic for future study.

Next, we briefly discuss how to calculate *p*-values and leave the detailed procedures into the Appendix B. According to Theorem 1, we can calculate *p*-values asymptotically. The *p*-values for individual iSPU(*γ*) can be calculated via either a normal or an extreme value distribution. The *p*-value for aiSPU can be calculated by the following two steps. First, by $\mathrm{cov}\left[L\left(t,\mu_{0}\right),L\left(s,\mu_{0}\right)\right]=o\left(pn^{-\left(t+s\right)/2}\right)$ if *s*+*t* is odd and by Theorem 1 part three, iSPU with even *γ*, odd *γ*, *γ* = ∞ are asymptotically independent to each other (see Appendix B for details). Because for a finite *γ*, L(γ)−ψ(γ)/ω(γ) follows a standard normal distribution, taking the minimum *p*-value as test statistics equals to taking the maximum of |L(γ)−ψ(γ)|/ω(γ) as the test statistics. Further define $t_{O}=\max_{\text{odd}\;\;\gamma \in \Gamma }\left|\left(L\left(\gamma \right)-\psi \left(\gamma \right)\right)\right|/\omega \left(\gamma \right)$ and $t_{E}=\max_{\text{even}\;\;\gamma \in \Gamma }\left(L\left(\gamma \right)-\psi \left(\gamma \right)\right)/\omega \left(\gamma \right)$ as the observed test statistics from the data and calculate the p-values for *t*_*O*_, *t*_*E*_, and *L*(∞) as $p_{O}=Pr\left[\max_{\text{odd}\;\;\gamma \in \Gamma }\left|\left(L\left(\gamma \right)-\psi \left(\gamma \right)\right)/\omega \left(\gamma \right)\right|>t_{O}\right]$, $p_{E}=Pr\left[\max_{\text{even}\;\;\gamma \in \Gamma }\left(L\left(\gamma \right)-\psi \left(\gamma \right)\right)/\omega \left(\gamma \right)>t_{E}\right]$, and *p*_∞_ equals to the *p*-value of iSPU(∞). Specifically, we use *pmvnorm()* in R package *mvrnorm* to calculate the normal tail probabilities of *p*_*O*_ and *p*_*E*_. Second, we take the minimum *p*-value from the above three categories, that is, $p_{\min}=\min\left\{p_{O},p_{E},p_{\infty }\right\}$. By the asymptotic independence among *p*_*O*_, *p*_*E*_, and *p*_∞_, the asymptotic p-value for the aiSPU test is $p_{\text{aiSPU}}=1-{\left(1-p_{\min}\right)}^{3}$.

Of note, calculating *ψ*(*γ*), *ω*(*γ*) and R(Γ′) involves $\text{Σ}=\left(\sigma_{kj}\right)$, which is unknown and has to be estimated in practice. We apply either the banding method of Bickel and Levina (2008) or a parametric bootstrap-based method to estimate covariance matrix Σ (see Remark S2 in Appendix B for details).

Meanwhile, we can calculate *p*-values by the parametric bootstrap (see Appendix B for details). The parametric bootstrap may estimates more accurately the *p*-values than the asymptotics-based method, but it is highly computational extensive, especially at a high significance level. To facilitate data analyses in the wider community, we have developed an R package “*aispu*”, implementing both methods.

**Remark 7** We recommend using the asymptotic-based method when p is large and using the parametric bootstrap-based method when p is small. Our proposed asymptotic-based method may have a better performance when p is large due to two reasons. First, residual bootstrap and pairs bootstrap are known to be problematic under a high-dimensional setting (El Karoui and Purdom, 2018). We expect that parametric bootstrap may have a similar problem when p is large. Second, by Theorem 1, estimation error can be ignored as both n and p go to infinity. On the other hand, when p is small, the parametric bootstrap-based method may achieve superior performance than the asymptotic-based method because the asymptotic theory in Theorem 1 may not hold.

### 2.5. Asymptotic power analysis

We analyze the asymptotic power of the aiSPU test. Under an alternative $H_{A}:\beta \neq 0$, we first derive approximations to the mean and variance of *L*(*γ, µ*_0_) with *γ <* ∞, denoted by $\psi_{A}\left(\gamma \right)=E\left[L\left(\gamma ,\mu_{0}\right)|H_{A}\right]$ and by $\omega_{A}^{2}\left(\gamma \right)=\mathrm{var}\left[L\left(\gamma ,\mu_{0}\right)|H_{A}\right]$, respectively. Then we derive the asymptotic power under a local alternative. In the end, we discuss the choice of the Γ set. To save space, we put technical details into the Appendix D.

First, we define some necessary notations. Let *β*^0^ be the true value of *β* and $\mu_{0}^{A}\equiv {\left(\mu_{01}^{A},\ldots ,\mu_{0n}^{A}\right)}^{\prime }$ with $\mu_{0i}^{A}=E\left(Y_{i}|X_{i},Z_{i};H_{A}\right)=g^{-1}\left(X_{i}\beta^{0}+Z_{i}\vartheta^{0}\right)$ being the conditional mean of *Y*_*i*_ under *H*_*A*_. We further define $\tilde{\psi }\left(\gamma \right)=E\left[L\left(\gamma ,\mu_{0}^{A}\right)|H_{A}\right]$ and ${\tilde{\omega }}^{2}\left(\gamma \right)=\mathrm{var}\left[L\left(\gamma ,\mu_{0}^{A}\right)|H_{A}\right]$.

The high dimensionality of *X*_*i*_ makes the identification of the leading order term of the test statistic *L*(*γ*) quite challenging. Here, we consider a local alternative such that for j=1,2,…,p, $\Delta_{j}=E\left[\left(\mu_{01}^{A}-\mu_{01}\right)X_{1j}\right]=O\left(n^{-1/2}{\left(\log p\right)}^{\kappa }\right)$ with *κ >* 0, which allows the identification of the leading order term. This condition restricts that ∆_*j*_ is a small term, which further implies that $\psi_{A}\left(\gamma \right)-\tilde{\psi }\left(\gamma \right)$ and $\omega_{A}^{2}\left(\gamma \right)-{\tilde{\omega }}^{2}\left(\gamma \right)$ are relatively small. Under the local alternative, we denote the set of locations of the signal variables by $S_{\eta }=\left\{j:\Delta_{j}\neq 0;1\leq j\leq p\right\}$ and the cardinality of $S_{\eta }$ by *p*^1−*η*^, where 0 ≤ *η* ≤ 1 is the parameter controlling the sparsity level.

We now analyze the power of the proposed aiSPU test. Let *p*_*α*_ be the critical threshold for the aiSPU test under *H*_0_ with the significance level *α*. Because $T_{\text{aiSPU}}=\min_{\gamma \in \Gamma }P_{L\left(\gamma \right)}$ the statistical power under *H*_*A*_ satisfies $Pr\left(T_{\text{aiSPU}}=\min_{\gamma \in \Gamma }P_{\text{iSPU}\left(\gamma \right)}<p_{\alpha }\right)\geq Pr\left(P_{\text{iSPU}\left(\gamma \right)}<p_{\alpha }\right)$. Thus the asymptotic power of aiSPU is 1 if there exists a *γ* ∈ Γ such that $Pr\left(P_{\text{iSPU}\left(\gamma \right)}<p_{\alpha }\right)\to 1$. In other words, to study the asymptotic power of the aiSPU, we only need to discuss the power of iSPU(*γ*) for *γ* ∈ Γ. For that purpose, Theorem 2 shows the asymptotic distribution of *L*(*γ, µ*_0_) with any finite and fixed *γ* under 0 ≤ *η <* 1*/*2.

Theorem 2. Under the assumptions C8–C9 in Appendix A and the alternative H_A_ with 0 ≤ η < 1/2 and $\Delta_{j}=O\left(n^{-1/2}{\left(\log p\right)}^{\kappa }\right)$ with κ > 0, for any fixed and finite Γ′ set, ${\left[\left\{L\left(\gamma ,\mu_{0}\right)-\psi_{A}\left(\gamma \right)\right\}/\omega_{A}\left(\gamma \right)\right]}_{\gamma \in \Gamma^{\prime }}^{'}$ converges weakly to a multivariate normal distribution with mean zero as n, p → ∞.

**Remark 8** Under the local alternative 0 ≤ η < 1/2, by noting that ${\left(\log p\right)}^{c\kappa }/p^{\eta }=o\left(1\right)$, we have $\psi_{A}\left(\gamma \right)-\tilde{\psi }\left(\gamma \right)=\sum_{j=1}^{p}\sum_{c=1}^{\gamma }\left(\begin{array}{c} \gamma \\ c \end{array}\right)\Delta_{j}^{c}O\left(n^{-\left(\gamma -c\right)/2}\right)=o\left(pn^{-\gamma /2}\right)$. Similarly, we have $\omega_{A}^{2}\left(\gamma \right)-{\tilde{\omega }}^{2}\left(\gamma \right)=o\left(pn^{-\gamma }\right)$. Then a proof similar to that of Theorem 1 for any fixed and finite Γ set (part one) yields Theorem 2.

For simplicity, we assume µ_0_ is known under H_A_ and derive Theorem 2 with L(γ) = L(γ, µ_0_). While this simplification ignores the estimation errors of ${\hat{\mu }}_{0}$ and thus induces a gap between Theorem 2 and our proposed test, Theorem 2 still provides useful insights regarding which iSPU(γ) achieves the highest power under different alternatives. These insights are in line with our simulation results. To establish Theorem 2 with estimated ${\hat{\mu }}_{0}$ is quite challenging because we need to estimate and quantify the estimation error of ${\hat{\mu }}_{0}$ under a misspecified model, which is unknown and an interesting question. We leave it for future research.

Theorem 2 gives the asymptotic power of iSPU(*γ*) at the significance level *p*_*α*_ as

$$Pr\left(P_{\text{iSPU}\left(\gamma \right)}<p_{\alpha }\right)=\{\begin{array}{ll} \Phi \left\{\frac{\psi_{A}\left(\gamma \right)-\tilde{\psi }\left(\gamma \right)-z_{p\alpha }\tilde{\omega }\left(\gamma \right)}{w_{A}\left(\gamma \right)}\right\}, & \gamma \;\text{is}\;\text{even},\\ \Phi \left\{\frac{\psi_{A}\left(\gamma \right)-\tilde{\psi }\left(\gamma \right)-z_{p_{{\;}_{\alpha }/2}}\tilde{\omega }\left(\gamma \right)}{w_{A}\left(\gamma \right)}\right\}+\Phi \left\{-\frac{\psi_{A}\left(\gamma \right)-\tilde{\psi }\left(\gamma \right)-z_{p_{{\;}_{{\;}_{\alpha }/2}}}\tilde{\omega }\left(\gamma \right)}{w_{A}\left(\gamma \right)}\right\}, & \gamma \;\text{is odd}, \end{array}$$

where Φ and $z_{p_{\alpha }}$ is the standard normal cumulative distribution function and its (1 − *p*_*α*_)th quantile, respectively. Because $\tilde{\omega }\left(\gamma \right)/\omega_{A}\left(\gamma \right)$ is bounded, the asymptotic power of iSPU(*γ*) is mainly determined by $\left\{\psi_{A}\left(\gamma \right)-\tilde{\psi }\left(\gamma \right)\right\}/\omega_{A}\left(\gamma \right)$. Further note that *ω*_*A*_(*γ*) is of order $p^{1/2}n^{-\gamma /2}$ and thus the power goes to 1 if $\left(\psi_{A}\left(\gamma \right)-\tilde{\psi }\left(\gamma \right)\right)n^{\gamma /2}p^{-1/2}\to \infty$. In particular, the asymptotic power of iSPU(1) and iSPU(2) goes to 1 if $p^{-1/2}n^{1/2}\sum_{i}\Delta_{i}\to \infty$ and $p^{-1/2}n\sum_{i}\Delta_{i}^{2}\to \infty$, respectively.

Note that iSPU(∞) is expected to lose power substantially when max_*j*_ |∆_*j*_| is small, i.e., $\max_{j}\left|\Delta_{j}\right|=o\left(\log{\left(p\right)}^{1/2}n^{-1/2}\right)$(Cai et al., 2014), while iSPU(1) and iSPU(2) are expected to be powerful under dense but weak signals (e.g., $\max_{j}\left|\Delta_{j}\right|=o\left(n^{-1/2}\right)$) alternatives. Thus, we discuss dense alternatives (0 ≤ *η <* 1/2) and sparse alternatives (*η* ≥ 1/2) separately.

Under different dense alternatives, different iSPU(*γ*) tests achieve the highest power. To further study the power of different iSPU tests and gain insights about how to choose the Γ set, we consider a particular alternative where the ∆_*j*_ is fixed at the same level. To be specific, we consider the local alternative such that $\Delta_{1}=\cdots =\Delta_{p}=\Delta =n^{-1/2}r^{1/2}$, where *r* → 0 as *n, p* → ∞. As shown in the Appendix D, under this alternative, iSPU(1) is more powerful than any other iSPU(*γ*) tests. Similarly, we show that iSPU(2) is asymptotically more powerful than other iSPU(*γ*) tests under the alternative where the absolute values of the ∆_*j*_ are the same but about half being positive while the other half being negative.

We then briefly discuss the sparse alternatives with *η >* 1*/*2. Under the sparse *H*_*A*_ with *η* ≥ 1/2, any iSPU test with a finite *γ* loses power. For example, for any *η <*1/2, the power of iSPU(1) converges to 1 when $p^{-1/2}n^{1/2}\sum_{j}\Delta_{j}\to \infty$; however, $\Delta_{j}=O\left(n^{-1/2}{\left(\log p\right)}^{\kappa }\right)$ and $\sum_{j}\Delta_{j}=p^{1-\eta }O\left(n^{-1/2}{\left(\log p\right)}^{\kappa }\right)$, leading to $p^{-1/2}n^{1/2}\sum_{j}\Delta_{j}\sim p^{1/2-\eta }{\left(\log p\right)}^{\kappa }\to 0$ when *η >* 1/2. Thus the asymptotic power of iSPU(1) is strictly less than 1 when *η* ≥ 1*/*2. For other finite *γ*, we have similar results. On the other hand, a supremum-type test like iSPU(∞) is known to be powerful against sparse alternatives (Cai et al., 2014), therefore, the asymptotic power of aiSPU is 1 if that of iSPU(∞) converges to 1.

Overall, we recommend including small *γ* values such as 1, 2 to maintain high power under dense alternatives. As to be shown in simulations, iSPU with a medium *γ* value is often the most powerful in a finite sample. To achieve a balance between the asymptotic and finite-sample performances, including medium *γ* values such as 3,…,6 in Γ is recommended. This recommendation is also supported by our previous studies (Xu et al., 2016; Wu et al., 2019). Because iSPU(∞) is powerful under the sparse alternative, we recommend including ∞ in Γ. In summary, we recommend use Γ={1,2,…,6,∞} as our default setting.

## 3. Simulations

### 3.1. Simulation settings

To facilitate fair and unbiased comparisons, we adopted the simulation settings similar to those in Lin et al. (2013); Zhang and Cheng (2017).

#### Simulation settings for *G* × *E* interactions.

We simulated genotypes as in Wang and Elston (2007). First, a latent vector $s={\left(s_{1},\ldots ,s_{p}\right)}^{\prime }$ was generated from a multivariate normal distribution N(0,V), where $V=\left(V_{kj}\right)$ had a first-order autoregressive covariance structure with $V_{kj}=\rho^{\left|k-j\right|}$. Second, a haplotype was generated by dichotomizing the latent vector *s* with some pre-specified minor allele frequencies (MAFs), each of which was randomly sampled from a uniform distribution between 0.1 and 0.3 for common variants (unless otherwise stated for rare variants). Third, the above two steps were repeated to generate two independent haplotypes and for subject *i*, the genotype value $G_{i}={\left(G_{i1},\ldots ,G_{ip}\right)}^{\prime }$ was the sum of the two haplotypes. We set *ρ* = 0 to generate independent SNPs unless otherwise stated.

As in Lin et al. (2013), we generated a binary outcome by the following logistic regression model

$$\text{logit}\left[P\left(Y_{i}=1|Z_{i},E_{i},G_{i}\right)\right]=\vartheta_{0}+\vartheta_{1}Z_{1i}+\vartheta_{2}Z_{2i}+\vartheta_{3}E_{i}+\vartheta_{4}^{'}G_{i}+\beta^{\prime }G_{i}\times E_{i},$$

where *ϑ*_0_ = log(0.4/0.6), *ϑ*_1_ = 0.05, *ϑ*_2_ = 0.057, *ϑ*_3_ = 0.64, and

$$\vartheta_{4}=\underset{q_{1}}{\underset{︸}{(0.4,\ldots ,0.4}},\underset{q_{2}}{\underset{︸}{-0.4,\ldots ,-0.4}},\underset{p-q_{1}-q_{2}}{\underset{︸}{{0,\ldots ,0)}^{\prime }}}.$$

*Z*_1_ was generated from a normal distribution while *Z*_2_ was generated from a Bernoulli distribution. Environmental variable *E* was generated from a Bernoulli distribution, taking on 1 and −1 with an equal probability. *G*_*i*_ × *E*_*i*_ is the gene-environmental interaction for subject *i*. As in a case-control study, we sampled *n*/2 cases and *n*/2 controls in each data set. We were interested in testing *H*_0_ to see whether there is any gene-environment interaction. Under *H*_*A*_, the gene-environmental interaction effect patterns are generally complex and unknown. For example, for xeroderma pigmentosum, there is no main genetic effect, but both environmental (ultraviolet light) effect and gene-environmental interaction effect exist (Hunter, 2005). To consider various scenarios, we randomly chose ⌊ps⌋ elements in *β* to be non-zero and their values were generated from a uniform distribution *U* (−*c, c*) unless otherwise stated.

#### Simulation settings for high-dimensional linear models.

We generated $X_{n\times p}$ and ${\mathbb{Z}}_{n\times q}$ from a multivariate normal distribution; that is, we had independent draws $X_{i}\sim N\left(0,\Xi_{1}\right)$ and $Z_{i}\sim N\left(0,\Xi_{2}\right)$ for i=1,…,n, where $\Xi_{1}$ and $\Xi_{2}$ were block diagonal symmetric matrices. The response *Y* was generated from a high-dimensional linear model:

Y=ℤϑ+Xβ+ϵ,

where $\vartheta ={\left(\vartheta_{1},\ldots ,\vartheta_{q}\right)}^{\prime }$, $\beta ={\left(\beta_{1},\ldots ,\beta_{p}\right)}^{\prime }$, and each element of *ϵ* followed a standard normal distribution. We set *ϑ*_1_ = *ϑ*_2_ = 0.4 and other *ϑ*_*j*_ = 0. We considered testing *H*_0_ and *H*_*A*_ in (2).

Under *H*_*A*_, ⌊ps⌋ elements in *β* were set to be non-zero, where *s* ∈ [0, 1] controlled the level of signal sparsity. The indices of non-zero elements of *β* were uniformly distributed, and their values were generated from a uniform distribution *U* (−*c, c*) unless specified otherwise. We set *n* = 200, *q* = 1000, and *p* = 1000.

For each simulation setting, we generated 1,000 data sets to evaluate the empirical size and power at the significance level *α* = 0.05. The candidate set of *γ* for the aiSPU was taken to be Γ={1,…,6,∞} unless otherwise stated.

To evaluate the effect of penalization, we further presented the results of aiSPU with two different ways of estimating the nuisance parameter *ϑ* under *H*_0_. First, we considered the oracle estimator, which is defined as the MLE with the knowledge/oracle about which covariates are non-informative (i.e. their effect size is 0) under *H*_0_, denoted as aiSPU(Oracle). Second, under the situation with *n > p*, we considered using the MLE to estimate *ϑ*, denoted as aiSPU(Full). Note that aiSPU(Full) equals to the aSPU (Wu et al., 2019).

For comparison, under *G* × *E* interaction settings, we applied GESAT (Lin et al., 2013) for common variants, and applied both iSKAT (Lin et al., 2016) and MiSTi (Su et al., 2017) for rare variants. To confirm that the theoretical null distribution of GESAT may not hold under a relatively high-dimensional situation, we calculated the *p*-value of GESAT by a simulation-based method, denoted as GESAT-sim. As a benchmark, we further considered the univariate minimum *p*-value (UminP) test, which first tests for SNP-environment interaction for each SNP, then takes their minimum *p*-value as the test statistic, and finally performs a corresponding Bonferroni adjustment. Under high-dimensional linear model settings, we conducted the three-step procedure with NST and ST statistics (Zhang and Cheng, 2017).

### 3.2. Results for *G* × *E* interactions

In many set-based *G* × *E* testing applications, the number of genetic variants *p* is relatively large but still smaller than the sample size *n*. Thus, we conducted two types of simulations: *n > p* or *n < p*.

#### Simulations with *n > p*.

First, we conducted simulations with *n* = 2000, *q*_1_ = 2, *q*_2_ = 0, and varying *p* to evaluate Type I error rates of different tests under different scenarios, ranging from low-dimensional to relatively high-dimensional. Note that the dimension of the nuisance parameter *ϑ* was *q* = *p* + 4, while that of the parameter *β* being tested was *p*. Table 1 shows the empirical Type I error rates, indicating that GESAT (Lin et al., 2013) yielded an inflated Type I error rate when *p* was large. Of note, even though by searching a much larger upper bound for the tuning parameter (say, $\sqrt{n}$ instead of the default $\sqrt{n/\log\left(n\right)}$) somewhat alleviated the problem, GESAT still yielded an inflated Type I error rate. For example, the Type I error rate of GESAT with tuning parameter searching up to $\sqrt{n}$ was 0.253 for the situation with *n* = 2000 and *p* = 300. In contrast, GESAT-sim maintained the correct Type I error rate, confirming that the theoretical null distribution of GESAT was not applicable when *q* was relatively large. As expected, aiSPU(Full) maintained the correct Type I error rate when *q* was relatively small and yielded an inflated Type I error rate when *q* was large, indicating penalized estimation of *ϑ* was necessary when *q* was relatively large. As expected, both aiSPU(Oracle) and aiSPU(TLP) yielded well-controlled Type I error rates for all the situations considered.

**Figure 1:**
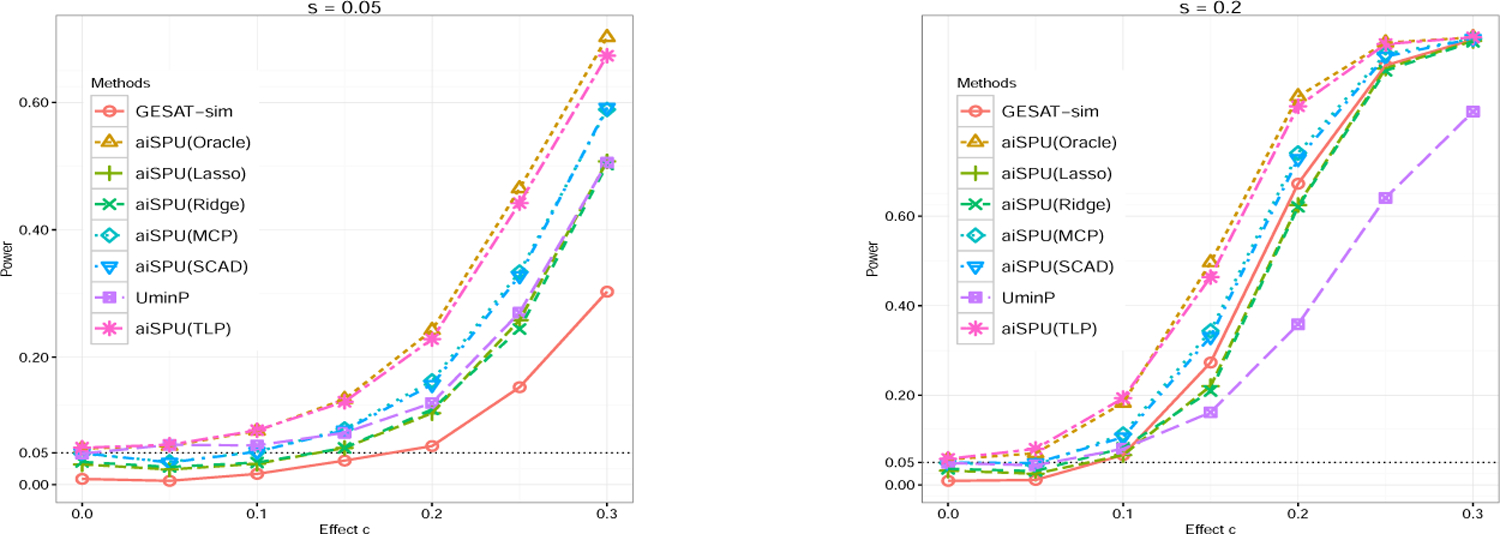
Power comparison for different methods in under *G* × *E* interaction simulations with *n* = 2000, *p* = 300 and *q*_1_ = *q*_2_ = 20. *n*, *p*, *q*_1_, and *q*_2_ stand for the sample size, number of terms in *G* × *E* interaction, number of the positive genetic main effects, and number of the negative genetic main effects, respectively. We varied the sparsity level *s*.

Next, we studied the effect of the number of non-zero nuisance parameters. Here we evaluated the performance of iSPU and aiSPU with some popular penalties, such as the Lasso and ridge. Table 2 shows the results of *n* = 2000, *p* = 300, and varying *q*_1_ = *q*_2_. When *q*_1_ = *q*_2_ was relatively large (*q*_1_ = *q*_2_ = 20 or *q*_1_ = *q*_2_ = 30), both the ridge and Lasso yielded slightly conservative Type I error rates and thus power loss (Figure 1). In contrast, aiSPU(TLP) provided results that were similar to those of aiSPU(Oracle). Again, GESAT yielded inflated Type I error rates because its theoretical null distribution was not applicable with relatively larger *p* and *p > n*. The results of *n* = 2000, *p* = 200, and varying *q*_1_ = *q*_2_ show similar conclusions (Table S1 in Appendix E).

To evaluate empirical power, we considered two cases: (a) under relatively low dimensional situations; (b) under relatively high-dimensional situations. Figure 1 shows the power of different methods under relatively high-dimensional situations with *n* = 2000, *p* = 300, and *q*_1_ = *q*_2_ = 20. Because both the Lasso and ridge yielded slightly conservative Type I error rates, aiSPU(Ridge) and aiSPU(Lasso) were less powerful than aiSPU(TLP). Perhaps because TLP better approximated the optimal *L*_0_ constraint (Shen et al., 2012), aiSPU(TLP) achieved higher power than aiSPU(MCP) and aiSPU(SCAD). As a benchmark, UminP performed relatively well when the signal was sparse. Figure S1 shows that iSPU with different *γ* was more powerful under different sparsity levels. However, due to its adaptivity, aiSPU was the overall winner (Figure S1 in Appendix E). The results for correlated SNPs (*ρ* = 0.3) or *q*_1_ = *q*_2_ = 50 showed similar patterns as in Figure 1 and thus were relegated to the Appendix E (Figures S2 and S3). Under relatively low-dimensional situations with *n* = 2000 and *p* = 25 or 50 (Figures S4 and S5), GESAT yielded well-controlled Type I error rates and achieved very similar power as GESAT-sim and iSPU(2). As expected, GESAT achieved higher power than iSPU(∞) under dense signal situations, but lower power than iSPU(∞) under sparse signal situations. In comparison, aiSPU achieved robustly high power under various scenarios. For the situation with *n* = 2000 and *p* = 75, while GESAT (regardless of how larger a searching region for the tuning parameter) had a slightly inflated Type I error rate, the results showed similar patterns as before (Figure S6 in Appendix E).

Next, similar to that in Su et al. (2017), we considered rare variants by generating SNPs with MAFs ranging from 0.005 to 0.05 while keeping the other simulation aspects unchanged. As expected, when *p* was relatively high, both iSKAT and MiSTi yielded inflated Type I error rates due to the theoretical null distribution is not applicable under relatively larger *p* and *p > n* situations. In contrast, aiSPU(TLP) maintained the correct Type I error rate under relatively high-dimensional situation (Tables S2 and S3 in Appendix E). Figure S7 shows the power comparison under the different low dimensional situations. Again, even though different tests may be more powerful under certain situations, aiSPU achieved robust high power across all the situations considered.

#### Simulations with *n < p*.

We conducted simulations with *n* = 200, *p* = 1000, *q*_1_ = 2, *q*_2_ = 2, and varying sparsity level *s*. Since GESAT yielded incorrect Type I error rates in high dimensional settings, the results of GESAT were not shown here.

First, we evaluated the performance of the asymptotic theory in Theorem 1 for finite samples. Table 3 shows the empirical Type I error rates and statistical power under *s* = 0.005. The iSPU and aiSPU yielded well-controlled Type I error rates. The results of the tests based on asymptotics were close to those based on the bootstrap, supporting Theorem 1. The results of other simulation settings (*s* = 0.001, *s* = 0.01,*s* = 0.05, *s* = 0.2, and informative variables in *β* were generated from a uniform distribution *U* (0, *c*)) showed similar patterns and were relegated into the Appendix E (Tables S4–S8). We further studied the situation when both main effects and interaction effects exist for the same set of SNPs and again showed similar patterns as expected (Table S9 in Appendix E).

**Figure 2:**
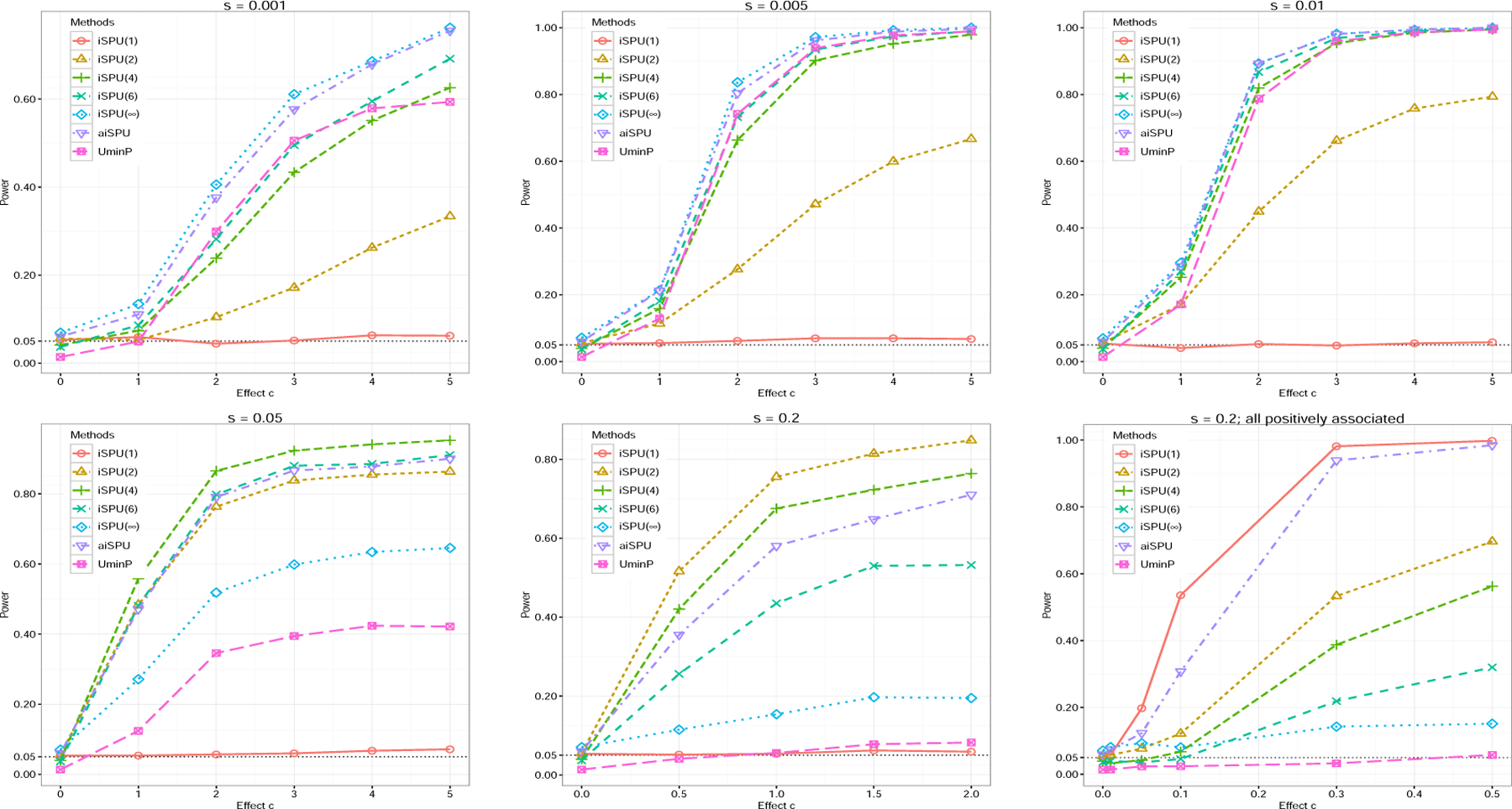
Power comparison for different methods under *G*× *E* interaction simulations with *n* = 200, *p* = 1000. We varied the sparsity level *s*. In last sub-figure, we generated informative variables in *β* from a uniform distribution *U* (0*, c*).

Next, we compared statistical power. Figure 2 shows the empirical power for the tests under different sparsity levels *s*. When the signal was highly sparse, iSPU(∞) was more powerful than other tests (*s* = 0.001 and *s* = 0.005). As signal became relatively sparse (*s* = 0.05), iSPU(4) was the most powerful, closely followed by iSPU(6) and aiSPU, demonstrating the power gain by using some iSPU(*γ*) test with 2 *< γ <* ∞ in a finite sample situation. When the signal became relatively dense with different association directions (*s* = 0.2), iSPU(2) was more powerful. For last sub-figure of Figure 2, we generated non-zero values of the parameter from a uniform distribution *U* (0*, c*) instead, and iSPU(1) was the winner. All these simulation results confirmed the previous asymptotic power analysis. By combining information from different iSPU tests, aiSPU was an overall winner, either achieving the highest power or having power close to that of the winner in any setting. In comparison, UminP achieved relatively high power when the signal was sparse (*s* = 0.001, *s* = 0.005, and *s* = 0.01), but lost power substantially when the signal was dense (*s* = 0.05 and *s* = 0.2).

**Figure 3:**
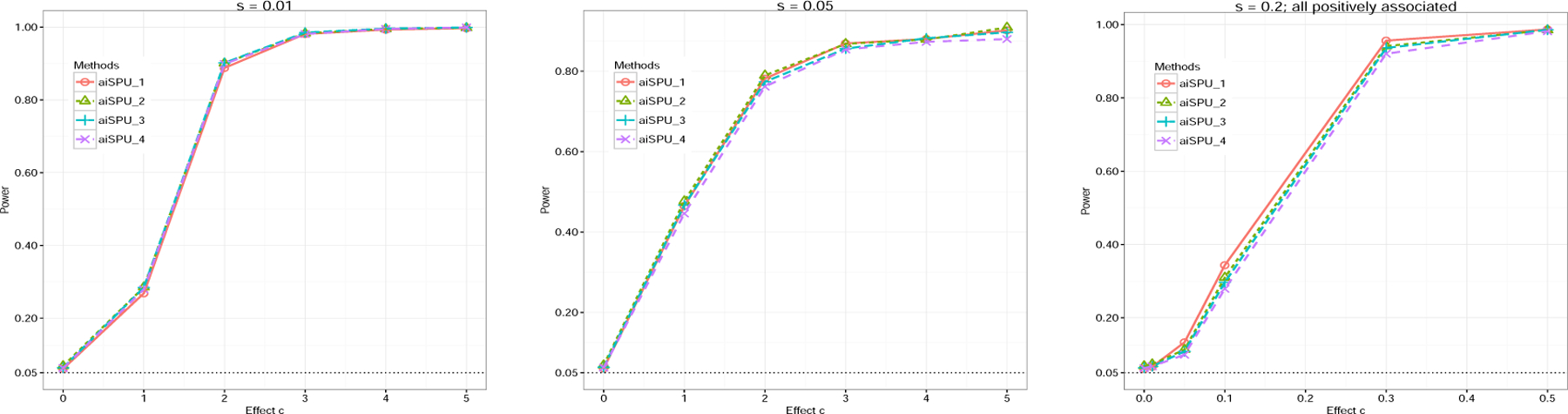
Empirical power of aiSPU with different Γ set under *G*×*E* interaction simulations with *n* = 200, *p* = 1000. aiSPU_1, aiSPU_2, aiSPU_3, aiSPU_4 represent aiSPU with $\Gamma_{1}=\left\{1,2,3,4;\infty \right\}$, $\Gamma_{2}=\left\{1,2,\ldots ,6,\infty \right\}$, $\Gamma_{3}=\left\{1,\ldots ,8,\infty \right\}$, and $\Gamma_{4}=\left\{1,2,\ldots ,10,\infty \right\}$, respectively. We varied the sparsity level *s*. In last sub-figure, we generated none-zero elements of *β* from a uniform distribution *U* (0*, c*).

Next, we briefly discussed the sensitivity of the aiSPU test to the choice of Γ set. Figure 3 shows the results of aiSPU with different Γ sets under different sparsity levels (*s* = 0.01, *s* = 0.05, and *s* = 0.2), indicating that the aiSPU test was robust to the choice of Γ. The results for other settings showed similar patterns and were relegated to the Appendix E (Figure S8).

**Figure 4:**
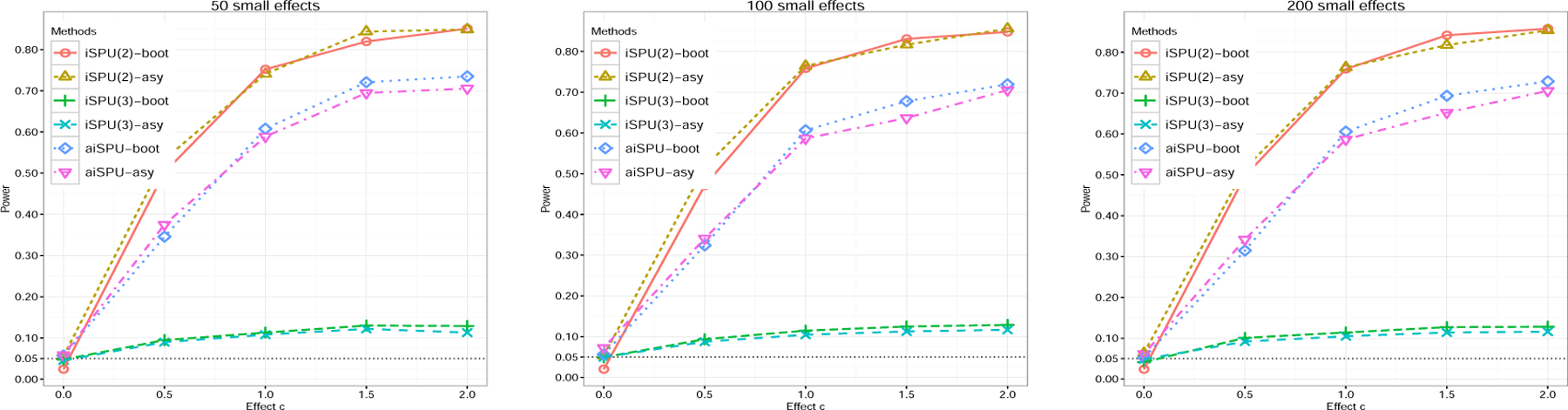
Empirical power of aiSPU under *G* × *E* interaction simulations with *n* = 200, *p* = 1000, and sparsity level *s* = 0.2. We randomly selected a pre-specified number of variables in ℤ and set the effect size followed a uniform distribution *U* (−0.01, 0.01). -boot and -asy stand for the results based on bootstrap and asymptotics, respectively.

Next, we briefly evaluated the robustness of the aiSPU test. Theorem 1 assumes that the effect of ℤ is sparse and strong. While this assumption is usually required by a penalized regression method, it might be violated in real applications. For example, under an omnigenic model (Liu et al., 2019), many variables in ℤ (i.e., SNPs) have weak effects, and only a few variables have strong effects. To evaluate the impact of the violation of the sparse effect assumption on ℤ, we kept the simulation setting unchanged except that we randomly selected a pre-specified number of variables in ℤ and set non-zero small effect sizes for those selected variables. Figure 4 shows that aiSPU yielded well-controlled Type I error rates and achieved high power. Perhaps because the contribution of the small-effect variables in ℤ is relatively small to the estimation of $\hat{Y}$, the results of the tests based on asymptotics were close to those based on the bootstrap, indicating Theorem 1 is relatively robust to the violation of sparse effect assumption on ℤ. We further varied the effect size for the randomly selected small effect variables in ℤ and obtained similar results (Figure S9 in Appendix E).

Next, we investigated whether aiSPU with other non-convex penalties such as SCAD (Fan and Li, 2001) and MCP (Zhang, 2010) would yield results similar to that with TLP. Perhaps because TLP enjoys the selection consistency and optimal parameter estimation under some mild assumptions (Shen et al., 2012), aiSPU(TLP) often achieved higher power than both aiSPU(MCP) and aiSPU(SCAD) (Figure S10). Interestingly, aiSPU(SCAD) yielded inflated Type I error rates under a linear model setting (Figure S11). In Summary, aiSPU(TLP) generally achieved higher power and controlled Type I error rates. Further- more, we have provided some theoretical guarantee for aiSPU(TLP) and thus recommend using aiSPU with TLP as our default setting.

### 3.3. Results for linear models

First, the aiSPU test maintained correct Type I error rates, for which the asymptotics- and bootstrap-based methods gave similar results under different sparsity levels and association directions (Tables S10–S13 in Appendix E). Similarly, both NST and ST yielded well-controlled Type I error rates (NST: 0.055 and ST: 0.061 at the significance level *α* = 0.05).

**Figure 5:**
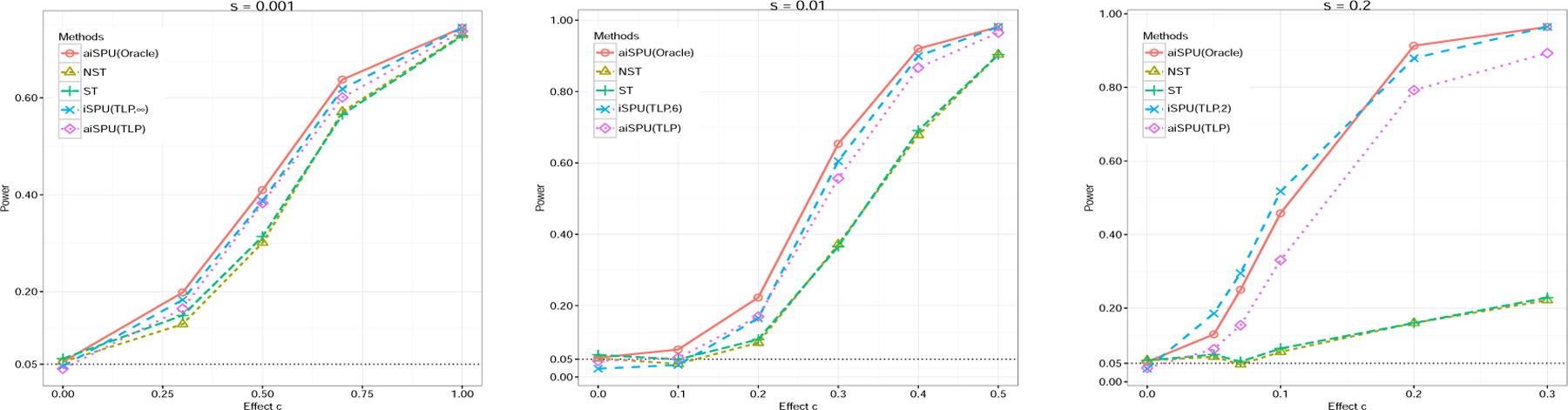
Power comparison for different tests under high-dimensional linear models simulations. We varied the sparsity level *s*.

Next, we assess statistical power. Figure 5 shows the empirical power for the tests under different sparsity levels *s*. Because the TLP estimator could consistently reconstruct the oracle estimator under mild assumptions (Shen et al., 2012), aiSPU(TLP) and aiSPU(Oracle) yielded similar results. Note that both NST and ST base their test statistics on a sub-sample, while aiSPU is on the whole sample; partly due to this difference in using the sample, aiSPU and iSPU(∞) were more powerful than both NST and ST even under a highly sparse alternative (i.e., with only one nonzero component in *β*; *s* = 0.001). Under other denser alternatives, aiSPU was way more powerful than both NST and ST. As in the simulations for *G* × *E* interaction, when the signal was relatively sparse (*s* = 0.01), iSPU(6) was the most powerful, highlighting the power gain by using some iSPU(*γ*) test with 2 *< γ <* ∞. In contrast, SPU(2) was more powerful when the signal became dense (*s* = 0.2). Again, all these simulation results confirmed the previous asymptotic power analysis. By combining different iSPU tests, aiSPU maintained high power across a wide range of alternative scenarios.

In the end, we briefly compared the computational time among some competing methods, the parametric bootstrap-based aiSPU, and the asymptotics-based aiSPU (Figure S12 in Appendix E), showing that the asymptotic-based aiSPU was generally computationally more efficient. Of note, we implemented penalized regression with TLP in R, which is not computationally efficient in high-dimensional settings. We expect that the computational time for the asymptotics-based aiSPU can be further reduced once we implement aiSPU in C or other more efficient computer languages.

In summary, owing to its adaptivity, the power of aiSPU remained high, being either the winner or close to the winner in any setting. In particular, the aiSPU(TLP) test performed similarly to aiSPU(Oracle) and yielded well-controlled Type I error rates, presumably because the TLP estimator could consistently reconstruct the oracle estimator under mild conditions.

## 4. Real data analyses

Alzheimer’s disease (AD) is the most common form of dementia, affecting millions of patients worldwide. The Alzheimer’s Disease Neuroimaging Initiative (ADNI) is a longitudinal, multisite observational study of elderly subjects with normal cognitive (healthy controls), mild cognitive impairment, or AD (Jack et al., 2008). The major goal of ADNI is to better understand the underlying mechanism of mild cognitive impairment (MCI) and AD (Jack et al., 2008). ADNI1 has recruited 819 elderly subjects to participate in the research. See www.adni-info.org for the latest information.

Several case-control studies suggest that AD is far more pronounced in females and gene-gender interaction may play roles in AD. Thus, we reanalyzed the ADNI1 data set to study whether the effect of genetic variants on AD risk is modified by gender.

Following set-ups in Altmann et al. (2014), we used the data of the Caucasian subjects in either the healthy control or MCI group, who had complete information on the environmental factor (gender) and covariates (age, years of education, and intracranial volume measured at baseline). For the outcome of interest, we set *Y*_*i*_ = 1 for any subject *i* in the MCI group, while setting *Y*_*i*_ = 0 for the other group. For the genotype data, we ran standard quality control steps to pre-process the data. In brief, we filtered out all SNPs with a genotyping rate *<* 0.95, those with a minor allele frequency *<* 0.05, and those failing to pass the Hardy-Weinberg equilibrium test (*p*-value *<* 10^−5^). Further, we imputed the missing SNPs by a Michigan Imputation Server (Das et al., 2016) with the 1000 Genomes Phase 1 v3 European samples as the reference panel. We restricted our analysis to the HapMap3 SNP subset and pruned SNPs with a criterion of linkage disequilibrium *r*^2^ *>* 0.2 using a sliding window of size 200 SNPs and a moving step of 20. According to the human genome reference hg19, we obtained the genomic coordinates of SNPs and genes, and assigned an SNP to a gene if it is located within 5,000 base pairs upstream or downstream of the gene’s coding region. We extracted candidate pathways from the KEGG database (Kanehisa et al., 2009). As other pathway-based analyses (O’Dushlaine et al., 2015; Pan et al., 2015), we restricted our analyses to the pathways containing between 10 and 200 genes. In total, we analyzed 578 subjects and 96 KEGG pathways. To account for multiple testing, we applied the Bonferroni correction and used a slightly conservative cutoff 0.05/100 = 5 × 10^−4^. Because other studies have reported an *APOE* gene and gender interaction on AD (Altmann et al., 2014), we tested the *APOE* and gender interaction as well. For testing main genetic effects, we applied aSPU (Pan et al., 2014) while adjusting for the same covariates as in testing *Gt* × *E* interactions.

Table 4 summarizes the results of our analysis. aiSPU identified one significant pathway “Fructose and mannose metabolism” (hsa00051, *p*-value = 0.0003) for *G* × *E* interaction, while GESAT failed to identify any significant pathways, showcasing possibly improved power of aiSPU over GESAT. Note that pathway “Fructose and mannose metabolism” contained 134 SNPs and thus, relative to the sample size, can be regarded as high-dimensional. The *p*-value of aiSPU was smaller than that of iSPU(1) and iSPU(2) but larger than iSPU(∞). Interestingly, aSPU failed to reject the null hypothesis of no main effects of the pathway (*p*-value = 0.54 by aSPU).

Next, we tested the *APOE* and gender interaction. Note that *APOE* contained 5 SNPs and can be viewed as a low dimensional situation. aSPU yielded a *p*-value of 0.007, confirming the strong association of *APOE* on AD. Further, aiSPU yielded a *p*-value of 0.039 for the *G* × *E* interaction, suggesting a potential *APOE* and gender interaction. In contrast, GESAT yielded a *p*-value of 0.56, failing to detect any *G* × *E* interactions. Similarly, with a Bonferroni-adjusted *p*-value of 0.30, UminP also failed to detect *G* × *E* interactions. By analyzing a large, multisite, longitudinal data from National Alzheimer’s Coordinating Center, Altmann et al. (2014) discovered *APOE* -gender interaction. They found that healthy female *APOE4* carriers had an almost 2-fold increased risk to develop MCI or AD when compared to female noncarriers (Altmann et al., 2014). By contrast, healthy male *APOE4* carriers had little increase in risk (Altmann et al., 2014). These findings support a possible interaction between *APOE* and gender on AD. In summary, our analyses have demonstrated that aiSPU is more powerful than GESAT in identifying gene-environment interactions when analyzing the ADNI1 data set.

## 5. Discussion

In this paper, we have proposed and studied an adaptive aiSPU test for high-dimensional parameters in GLMs in the presence of high-dimensional nuisance parameters. Our proposed aiSPU test takes advantage of both the TLP estimator (Shen et al., 2012) and data adaptive testing ideas (Pan et al., 2014), and thus enjoys several theoretical and practical benefits: first, the Type I error rate is well controlled; second, it maintains high statistical power under various scenarios, ranging from highly sparse to highly dense alternatives; third, it is computationally efficient as its *p*-values can be calculated via its asymptotic null distribution.

Several new methods (Ma et al., 2020; Shi et al., 2019; Sur and Candès, 2019; Fei and Li, 2019; Zhu et al., 2019) have recently been proposed for statistical inference with high-dimensional generalized linear models. However, they mainly focused on related but different questions with different approaches. Specifically, Ma et al. (2020) considered a global testing problem using a debiased Lasso based method with generalized low-dimensional projection. Sur and Candès (2019) quantified and corrected the bias of maximum likelihood estimators when the sample size and the dimensionality of parameters are in the same order. Fei and Li (2019) proposed a multi-sample splitting and averaging method to test a fixed subset of parameters. Shi et al. (2019) and Zhu et al. (2019) extended the score/Wald/likelihood ratio tests to (non-convex) penalized/constrained regression to test a subset of parameters of size much smaller than the sample size. In principle, due to its data-adaptive feature, aiSPU (with suitable modifications) may be a powerful tool to tackle these related problems, though rigorous investigation is warranted. We leave it for future research.

We conclude with several potential extensions of our approach. First, as transcriptome-wide association studies (TWAS) (Gamazon et al., 2015; Gusev et al., 2016) that incorporate eQTL-derived weights into a weighted Sum test (Xu et al., 2017) to both improve statistical power and enhance biological interpretation, our proposed method can incorporate eQTL-derived weights into the test statistics of iSPU(*γ*) and aiSPU. Also, some other functional weights (He et al., 2017; Ma and Wei, 2019) can be equally applied. We expect that integrating functional genomic information will improve power and gain insights into the mechanisms of complex traits. Second, we mainly considered interactions between a genetic marker set and an environmental variable. We expect the same approach can be applied to other biological problems. For example, by replacing the environmental variable *E* with a treatment, we can test for interactions between a genetic marker set and the treatment, which is at the core of personalized medicine. More generally, our method can be potentially applied to other high-dimensional problems. For example, with some technical modifications, our method may be capable of simultaneous inference on submatrices of a high-dimensional precision matrix. The proposed method can also be extended to the asymptotically independent *U* -statistics framework as recently introduced in He et al. (2020). We leave these for future research.

## Supplementary Material

1

## Figures and Tables

**Table 1:** Empirical Type I error rates of various tests for *G* × *E* interaction in simulations with *n* = 2000, *q*_1_ = 2, *q*_2_ = 0, and varying *p*. *n*, *p*, *q*_1_, and *q*_2_ stand for the sample size, number of terms in *G* × *E* interaction, number of the positive genetic main effects, and number of the negative genetic main effects, respectively.

*P*	25	50	70	100	200	300	400	500
GESAT	0.061	0.055	0.090*	0.103*	0.277*	0.636*	0.944*	1.000*
GESAT-sim	0.050	0.048	0.062	0.050	0.051	0.044	0.051	0.047
aiSPU(Full)	0.071	0.057	0.080*	0.085*	0.199*	0.551*	0.944*	1.000*
aiSPU(Oracle)	0.067	0.049	0.064	0.052	0.052	0.046	0.057	0.047
aiSPU(TLP)	0.061	0.054	0.057	0.053	0.053	0.042	0.060	0.047

* Inflated Type I error rates.

**Table 2:** Empirical Type I error rates of various tests for *G* × *E* interaction in simulations with *n* = 2000, *p* = 300 and varying *q*_1_ = *q*_2_. *n*, *p*, *q*_1_, and *q*_2_ stand for the sample size, number of terms in *G* × *E* interaction, number of the positive genetic main effects, and number of the negative genetic main effects, respectively.

*q*_1_ = *q*_2_	2	5	7	10	20	30
GESAT	0.637*	0.636*	0.628*	0.641*	0.657*	0.633*
GESAT-sim	0.043	0.030	0.026	0.010**	0.004**	0.002**
aiSPU(Ridge)	0.058	0.046	0.045	0.027	0.023**	0.017**
aiSPU(Lasso)	0.048	0.039	0.035	0.028	0.023**	0.016**
aiSPU(Full)	0.584*	0.594*	0.598*	0.634*	0.690*	0.712*
aiSPU(Oracle)	0.054	0.057	0.054	0.056	0.062	0.055
aiSPU(TLP)	0.058	0.052	0.057	0.053	0.058	0.057

* Inflated Type I error rates;

** Conservative Type I error rates.

**Table 3:** Empirical Type I errors and power (in percentage) of various tests under *G* × *E* interactions with *p* = 1000 and *n* = 200. Zero signal strength *c* = 0 represents Type I errors, while c≠0 represents powers. The sparsity level was *s* = 0.005, leading to 5 non-zero elements in *β*. The results outside and inside parentheses were calculated from parametric bootstrap- and asymptotics-based methods, respectively.

*c*	0	1	2	3	4	5
iSPU(1)	4.9 (4.8)	5.9 (5.6)	6.2 (6.1)	6.4 (6.6)	5.8 (6)	5.8 (5.7)
iSPU(2)	2.6 (5.2)	6.8 (11.8)	22.2 (28.5)	43.5 (47.5)	58.2 (61.3)	64.2 (67.8)
iSPU(3)	5.8 (5.6)	8.5 (8.1)	29.9 (28.7)	52.2 (51.4)	63.9 (62.1)	70.3 (69.2)
iSPU(4)	3 (3.9)	14.9 (17.1)	65.7 (67.1)	89.7 (90.4)	96 (96)	98.2 (98.4)
iSPU(5)	5.9 (5)	17 (15.6)	61.5 (60)	82.8 (81.3)	90.1 (88.9)	92.3 (92.5)
iSPU(6)	3.7 (3.2)	21.8 (19)	75.6 (74)	94.9 (93.7)	98.4 (97.9)	99.2 (99.2)
iSPU(∞)	8.5 (7.5)	26.8 (22.2)	85 (83.3)	97.6 (97.4)	99.6 (99.6)	100 (100)
aiSPU	5.8 (6.1)	20.7 (21.5)	79.4 (80.5)	95.4 (96.1)	98.8 (99.4)	99.8 (99.7)

**Table 4:** P-values from the association analysis of the ADNI1 data set to detect interactions between gender and genetic variants (in KEGG pathway hsa00051 or gene APOE).

	iSPU(*γ*)							aiSPU	GESAT
	*γ* = 1	*γ* = 2	*γ* = 3	*γ* = 4	*γ* = 5	*γ* = 6	*γ* = ∞		
hsa00051	0.017	0.017	0.014	0.010	0.006	0.003	0.0001	0.0003	0.016
*APOE*	0.022	0.032	0.042	0.059	0.068	0.079	0.112	0.039	0.56

## Appendix A. Assumptions

We further decompose *ϑ*^0^ into two parts:$\vartheta^{0}\equiv {\left(\vartheta_{1}^{0},\ldots ,\vartheta_{q}^{0}\right)}^{\prime }={\left(\vartheta_{A_{0}}^{0},0_{A_{0}^{c}}\right)}^{\prime }$, where $A_{0}\equiv \left\{j:\vartheta_{j}^{0}\neq 0\right\}$ is the set of nonzero coefficients of *ϑ*^0^ with size |*A*_0_| = *q*_0_, and $0_{A_{0}^{c}}$ is a vector of 0’s. Define $\tilde{\mathbb{Z}}$ as the (nuisance) covariate matrix containing the variables in *A*_0_, and the oracle estimate ${\hat{\vartheta }}^{o}$ as the maximum likelihood estimate (MLE) given that *A*_0_ is known priori.

We need the following assumptions to establish the asymptotic null distribution.

*C1.* The eigenvalues of Σ are bounded, that is, $B^{-1}\leq \lambda_{\min}\left(\text{Σ}\right)$, $\lambda_{\max}\left(\text{Σ}\right)\leq B$ for some finite constant *B*, where *λ*_min_(Σ) and *λ*_max_(Σ) denote the minimum and maximum eigenvalues of matrix Σ, respectively. Moreover, the absolute value of any corresponding correlation element is strictly smaller than 1, i.e., $\max_{1\leq i\neq j\leq p}\left|\sigma_{ij}\right|/\sqrt{\sigma_{ij}\sigma_{ij}}<1-\xi$ for some constant *ξ >* 0.

*C2.* Under *H*_0_ : *β* = 0, we have $E\left[S_{1j}^{3}\right]=0$ 0 for 1 ≤ *j* ≤ *p*. There exist some constants *q* and *K*_0_ *>* 0 such that $E\left[\exp\left(\varrho S_{1j}^{2}/\sigma_{jj}\right)\right]\leq K_{0}$ for 1 ≤ *j* ≤ *p*.

*C3. $\tilde{\mathbb{Z}}$*is uniformly bounded. We further assume $E\left(X_{1j}|\tilde{\mathbb{Z}}\right)\neq 0$ only holds for $j\in P_{0}\subset \left\{1,\ldots ,p\right\}$ with the size of *P*_0_, denoted by *p*_0_, satisfying $p_{0}=O\left(p^{\eta_{1}}\right)$ for a small positive *η*_1_.

*C4.* We assume $\frac{1}{p}\sum_{j_{1},j_{2}}\left|E\left[S_{1j_{1}}S_{1j_{2}}\right]\right|=O\left(1\right)$ and $\frac{1}{p}\sum_{j_{1}\notin P_{0},j_{2}\notin P_{0}}\left|E\left[X_{ij_{1}}X_{ij_{2}}|\tilde{\mathbb{Z}}\right]\right|=O\left(1\right)$.

*C5.* We assume $q\leq \exp\left(nC_{\min}\left(\vartheta^{0}\right)/d_{0}\right)$ and $pq_{0}^{4}/n^{2}=o\left(1\right)$, where *d*_0_ is some constant, $C_{\min}\left(\vartheta^{0}\right)\equiv \inf_{\left\{\vartheta^{A}=(\vartheta_{A},0_{A^{c}}:A\neq A_{0},\left|A\right|\leq q_{0}\right\}}-\log(1-h^{2}\left(\vartheta^{A},\vartheta^{0}\right)/\max\left(\left|A_{0}\backslash A\right|,1\right)$, and h(⋅,⋅) is the Hellinger distance. We further assume the model is sparse under the null, that is, $q_{0}=O\left(n^{\eta_{2}}\right)$ for a small positive *η*_2_.

*C6.* There exist some positive constants *K*_1_ and *K*_2_ such that $K_{1}<E\left[\epsilon_{0i}^{2}|\mathbb{Z}=z\right]<K_{2}$, where $\epsilon_{0i}=Y_{i}-\mu_{0i}$, 1 ≤ *i* ≤ *n*. We further assume $\lim\inf_{n\to \infty }n^{-1}\lambda_{\min}\left({\tilde{\mathbb{Z}}}^{\prime }W\tilde{\mathbb{Z}}\right)>0$, where $W=\text{diag}\left\{E\left(\epsilon_{01}^{2}|\mathbb{Z}\right),\ldots ,E\left(\epsilon_{0n}^{2}|\mathbb{Z}\right)\right\}$.

*C7. $-\log\left(1-h^{2}\left(\vartheta ,\vartheta^{0}\right)\right)\geq -d_{1}\log\left(1-h^{2}\left(\vartheta_{\tau^{+}},\vartheta^{0}\right)\right)-d_{3}q\tau^{d_{2}}$*for some constants *d*_1_, *d*_2_, and *d*_3_, where *d*_1_ − *d*_3_ *>* 0, $\vartheta_{\tau^{+}}=\left(\vartheta_{1}I\left(\left|\vartheta_{1}\right|\geq \tau \right),\ldots ,\vartheta_{q}I\left(\left|\vartheta_{q}\right|\geq \tau \right)\right)$ and *h*(*ϑ, ϑ*^0^) is the Hellinger distance between the two probability distributions specified by *ϑ* and *ϑ*^0^. For some constant *c*_0_ and any $\epsilon /2^{4}<t<\epsilon \leq 1$, $H\left(t,B_{A}\right)\leq c_{0}{\left(\log q\right)}^{2}\left|A\right|\log\left(2\epsilon /t\right)$, with |*A*| ≤ *q*_0_, where $H\left(\cdot ,B_{A}\right)$ is the bracketing Hellinger metric entropy of $B_{A}$, $B_{A}=F_{A}\cap \left\{h\left(\vartheta ,\vartheta^{0}\right)\leq 2\epsilon \right\}$ is a local parameter space, and $F_{A}=\left\{g^{1/2}\left(\vartheta ,y\right)\right\}$ is a collection of square root densities with *g*(*ϑ, y*) be a probability density for *Y*_1_.

*C8.* Under $H_{A}:\beta \neq 0$, we have $E\left[{\left({\tilde{S}}_{ij}\right)}^{3}\right]=0$ for 1 ≤ *j* ≤ *p*.

*C9. $\tilde{W}=\left\{{\tilde{W}}^{\left(j\right)}=\left({\tilde{S}}_{ij},i=1,\ldots ,n\right):j\geq 1\right\}$*is *α*-mixing such that $\alpha_{\tilde{W}}\left(s\right)\leq M\delta^{s}$, where *δ* ∈ (0, 1) and *M* is some constant.

**Remark S1** Assumption C1 is commonly used in the high-dimensional setting (Cai et al., 2014; Wu et al., 2019), assuming that the underlying true covariance matrix Σ is non-singular. Assumption C2 assumes sub-Gaussian-type tails of S_1j_, which is also common. Both assumptions C1 and C2 are only used to establish the weak convergence of L(∞, µ_0_) and not needed for L(γ, µ_0_) with a finite γ.

Assumption C3 assumes the underlying true model under H_0_ is sparse, which is often reasonable in real data applications and penalized regression framework. Note that we assume that each $X_{\dot{j}}$ is centered, which partially supports the assumption that $E\left[X_{ij}|\tilde{\mathbb{Z}}\right]\neq 0$ only for j ∈ P_0_ with the size of P_0_ in a small order of p, i.e., $p_{0}=O\left(p^{\eta_{1}}\right)$. For example, in our motivating gene-environment interaction testing problems, $X_{ij}=G_{ij}\times E_{i}$ and $E\left[X_{ij}|\tilde{\mathbb{Z}}\right]\neq 0$ holds if and only if $E\left[G_{ij}|\tilde{\mathbb{Z}}\right]\neq 0$, where $\tilde{\mathbb{Z}}$ contains common covariates, environmental factors, and important SNPs selected by our penalized regression model. Of note, genome-wide association studies with around a hundred thousand subjects only identified from a few hundred to a few thousand significant SNPs for each of the traits, which were some tiny proportions of all the SNPs being tested (about 10 million) (Buniello et al., 2018). In other words, the majority of SNPs G_j_ are independent of common covariates. Furthermore, because linkage disequilibrium (LD) is often local, SNP G_j_ is only correlated with a small proportion of the SNPs being tested (see Figure S13 for an example). Then $E\left[X_{ij}|\tilde{\mathbb{Z}}\right]\neq 0$ only for j ∈ P_0_ with the size of P_0_, $p_{0}=O\left(p^{\eta_{1}}\right)$. One caveat is that even though C3 usually holds for genetic and genomic data, C3 may fail in other applications, perhaps leading to Theorem 1 invalid. We leave this interesting topic for future research.

Assumption C4 is a moment assumption and assumes a weak dependence structure. Intuitively speaking, many random vectors meet this moment assumption. For example, random vectors $\zeta ={\left(\zeta_{1,}\zeta_{2},\ldots \right)}^{\prime }$, where ζ_i_ only correlates a finite number of ζ_j_; then ζ satisfies moment condition. It also includes an α-mixing type weak dependence as a special case, which has been broadly used in time series and spatial statistics and adopted previously in high-dimensional testing problems (Xu et al., 2016; Wu et al., 2019). To account for the effects of nuisance parameters, we further assume conditionally moment assumption, which is a natural extension of the moment assumption.

Assumption C5 is a relatively strong assumption needed to prove Theorem 1. It imposes some restrictions on the growth rate of p such that $p=O\left(n^{2-\eta_{3}}\right)$ for a small positive η_3_. Zhang and Cheng (2017) assumed ${\left(\log\left(pn\right)\right)}^{7}/n\leq N_{1}n^{-N_{2}}$ for some positive constants N_1_ and N_2_ to establish the theory for a bias-correction based test statistic, which is weaker than C5. A stronger condition is needed here to establish the joint asymptotic distribution of L(γ) with different γ’s. Nevertheless, C5 allows p/n → ∞. Assumption C5 imposes a weak restriction on q, allowing exponentially many nuisance parameters $q=\exp\left(nC_{\min}\left(\vartheta^{0}\right)/d_{0}\right)$. Shen et al. (2012) showed that this is a necessary condition for TLP to be selection consistent. C5 also assumes the sparsity on the ϑ^0^, which is common adopted by penalized regression and by bias correction-based methods. For example, under the nearly optimal condition q_0_ = o(n/(log p)^2^), the debiased Lasso estimator follows a Gaussian distribution asymptotically (Javanmard and Montanari, 2018). Also, the sparsity assumption regarding q_0_ may be relaxed. For example, the sparsity assumption is q_0_ = o(n/ log p) in a directed graphical model with TLP constraint (Li et al., 2019). More importantly, the sparsity assumption might not be essential for our proposed method. As shown in the simulation section (Figures 4 and S9), our proposed method still worked when the sparsity assumption was violated.

The first part of Assumption C6 is common in GLMs, for example, as adopted in Fan and Song (2010); Guo and Chen (2016). By Theorem 5.39 in Vershynin (2010), we have $\lim\inf_{n\to \infty }n^{-1}\lambda_{\min}\left({\tilde{\mathbb{Z}}}^{\prime }W\tilde{\mathbb{Z}}\right)>0$ with high probability. To simplify the technical details in the proof of the weak convergence result in Theorem 1, here we assume $\lim\inf_{n\to \infty }n^{-1}\lambda_{\min}\left({\tilde{\mathbb{Z}}}^{\prime }W\tilde{\mathbb{Z}}\right)>0$.

Assumption C7 is needed for feature selection consistency and optimal parameter estimation by TLP for GLMs (Shen et al., 2012). However, for linear and logistic regression, Assumption C7 can be substituted by the following C7* for verification purpose (Shen et al., 2012).

C7*.$\gamma_{\min}^{2}\min_{A:\left|A\right|\leq q_{0},A\neq A_{0}}\lambda_{\min}\left({\text{Θ}}_{A_{0}\backslash A}-{\text{Θ}}_{A_{0}\backslash A,A}{\text{Θ}}_{A}^{-1}{\text{Θ}}_{A,A_{0}\backslash A}\right)\geq d_{0}\frac{\log q}{n}$, where d_0_ is some constant independent of (n, q, q_0_), Θ is the covariance of ℤ with the jkth element cov(Z_j_, Z_k_), ${\text{Θ}}_{A_{0}\backslash A}$ is a submatrix of Θ by keeping rows and columns corresponding to a subset A_0_ \ A of predictors, and ${\text{Θ}}_{A_{0}\backslash A,A}$ is a submatrix of Θ by keeping rows corresponding to a subset A_0_ \ A of predictors and columns corresponding to a subset A of predictors, $\gamma_{\min}=\gamma_{\min}\left(\vartheta^{0}\right)\equiv \min\left\{\left|\vartheta_{k}^{0}\right|:\vartheta_{k}^{0}\neq 0\right\}$ is the resolution level of the true regression coefficients, and $\lambda_{\min}\left({\text{Θ}}_{A_{0}\backslash A}-{\text{Θ}}_{A_{0}\backslash A,A}{\text{Θ}}_{A}^{-1}{\text{Θ}}_{A,A_{0}\backslash A}\right)\geq \min_{B⊃A_{0}:\left|B\right|\leq 2q_{0}}\lambda_{\min}\left({\text{Θ}}_{B}\right)$.

Of note, C7 involves Hellinger distance, which is hard to verify in practice. For verification purposes, we propose a stronger than needed assumption C7*, which is sufficient for C7. C7* imposes a lower bound for coefficient strength (like a beta-min condition), which might be violated in practice. However, our proposed method might still work when assumption C7* (i.e., beta-min like assumption) is violated but C7 holds. In addition, we use this technical assumption to prove the TLP-based estimator achieves feature selection consistency, and thus the difference between µ_0_ and ${\hat{\mu }}_{0}$ can be well controlled. In practice, as long as we have a good estimate ${\hat{\mu }}_{0}$, our proposed method works. For example, as shown in simulations, our proposed method aiSPU still worked when the coefficient for ℤ was non-sparse but with sparse and strong signals, which violated the assumption C7* but not necessarily C7. To further illustrate this, we provide an example. Suppose the coefficient $\vartheta^{0}={\left(1,\frac{1}{\sqrt{n}},\frac{1}{\sqrt{n}},\cdots ,\frac{1}{\sqrt{n}}\right)}^{\prime }$, $\tau =\frac{1}{2\sqrt{2}}$ and d_1_ = 1. Then log(1 − h^2^(ϑ, ϑ^0^)) equals to $\log\left(1-h^{2}\left(\vartheta_{\tau^{+}},\vartheta^{0}\right)\right)$. This leads to the assumption C7 holds, coefficient for ℤ is non-sparse, and C7* may not hold. A similar example has been provided for TLP in the context of constrained maximum likelihood inference (Zhu et al., 2019). We leave the exciting topic on further relaxing Assumption C7 for future research.

## Appendix B. Calculating *p*-values

In this subsection, we describe how to calculate *p*-values by both parametric bootstrap and asymptotics-based methods in details.

### Asymptotics-based method

First, we calculate *p*-values for iSPU separately. We apply the Theorem 1 to approximate *ψ*(*γ*), *ω*(*γ*) and R(Γ′) = (*ρ*_*st*_), respectively. Specifically, ψ(1)=0, $\psi \left(\gamma \right)=\frac{\gamma !}{d!2^{d}}n^{-d}\sum_{j=1}^{p}\sigma_{jj}^{d}+o\left(pn^{-d}\right)$ if *γ* = 2*d* and $\psi \left(\gamma \right)=o\left(pn^{-\left(d+1\right)}\right)$ if *γ* = 2*d* + 1; $\omega^{2}\left(1\right)=\frac{1}{n}\sum_{1\leq i,j\leq p}\sigma_{ij}+o\left(pn^{-1}\right)$ and

$$\omega^{2}\left(\gamma \right)=\psi \left(2\gamma \right)-\sum_{j=1}^{p}{\left\{\psi^{\left(j\right)}\left(\gamma \right)\right\}}^{2}+\frac{1}{n^{\gamma }}\sum_{i\neq j}\sum_{\begin{array}{l} 2c_{1}+c_{3}=\gamma \\ 2c_{2}+c_{3}=\gamma \\ c_{3}>0 \end{array}}\frac{{\left(\gamma !\right)}^{2}}{c_{3}!c_{1}!c_{2}!2^{c_{1}+c_{2}}}\sigma_{ii}^{c_{1}}\sigma_{jj}^{c_{2}}\sigma_{ij}^{c_{3}}+o\left(pn^{-\gamma }\right)$$

if *γ* ≥ 2;

$$\begin{array}{l} \mathrm{cov}\left[L\left(t,\mu_{0}\right),L\left(s,\mu_{0}\right)\right]\\ =\psi \left(t+s\right)-\sum_{j=1}^{p}\psi^{\left(j\right)}\left(t\right)\psi^{\left(j\right)}\left(s\right)+\frac{1}{n^{c}}\sum_{k\neq j}\sum_{\begin{array}{c} 2c_{1}+c_{3}=t\\ 2c_{2}+c_{3}=s\\ c_{3}>0 \end{array}}\frac{t!s!}{c_{3}!c_{1}!c_{2}!2^{c_{1}+c_{2}}}\sigma_{kk}^{c_{1}}\sigma_{jj}^{c_{2}}\sigma_{kj}^{c_{3}}+o\left(pn^{-\left(t+s\right)/2}\right) \end{array}$$

if *s*+*t* is even and $\mathrm{cov}\left[L\left(t,\mu_{0}\right),L\left(s,\mu_{0}\right)\right]=o\left(pn^{-\left(t+s\right)/2}\right)$ if *s*+*t* is odd; *ρ*_*ss*_ = 1 for *s* ∈ Γ′ and $\rho_{st}=\mathrm{cov}\left[L\left(s,\mu_{0}\right),L\left(t,\mu_{0}\right)\right]/\left(\omega \left(s\right)\omega \left(t\right)\right)$ for $s\neq t\in \Gamma^{\prime }$. Then by Theorem 1, the *p*-values for individual iSPU(*γ*) can be calculated via either a normal or an extreme value distribution.

**Remark S2** In practice, Σ = (σ_kj_) is unknown and has to be estimated, which is a challenging problem under a high-dimensional setting. We discussed two situations separately: when Σ satisfies certain structures and when the structure is unknown.

When the covariance matrix Σ satisfies certain structures, we can apply some existing methods, such as banding and thresholding techniques (Bickel and Levina, 2008; Cai and Liu, 2011). See Fan et al. (2016) for an excellent review. For example, we can apply the banding method of Bickel and Levina (2008) to estimate covariance matrix Σ if the following α-mixing assumption holds: $W=\left\{W^{\left(j\right)}=\left(S_{ij},i=1,\ldots ,n\right):j\geq 1\right\}$ is α-mixing such that $\alpha_{W}\left(s\right)\leq M_{1}\delta_{1}^{s}$, where δ_1_ ∈ (0, 1) and M_1_ is some constant. Specifically, we calculate the sample covariance matrix $S=\left(s_{kj}\right)$ and then calculate the bandable covariance matrix as ${\hat{\Sigma }}_{k_{n}}=\left(s_{kj}I\left(\left|k-j\right|\leq k_{n}\right)\right)$. An optimal bandwidth k_n_ has been selected by five-fold cross- validation. For a properly chosen k_n_ = o(n^1/2^), the difference induced by estimating Σ is ignorable (Xu et al., 2016; Wu et al., 2019). We further define $\hat{\psi }\left(\gamma \right)$ and ω^2^(γ) as the corresponding estimated ψ(γ) and ω^2^(γ) by replacing Σ with ${\hat{\Sigma }}_{k_{n}}$. Under the mixing assumption, for any j, k, and ϵ > 0, there exists some constant C such that $\sigma_{kj}\leq C\delta^{\left|k-j\right|\epsilon /\left(2+\epsilon \right)}$, where δ ∈ (0, 1) (Guyon, 1995). Then for k_n_ → ∞ as n → ∞, the summation of terms involving σ_kj_ with |k − j| > k_n_ in ω^2^(γ) is ignorable, i.e., $n^{-\gamma }\sum_{k\neq j;\left|k-j\right|>k_{n}}C\sigma_{kk}^{c_{1}}\sigma_{jj}^{c_{2}}\sigma_{kj}^{c_{3}}=o\left(pn^{-\gamma }\right)$. Furthermore, there are O(k_n_p) terms in ω^2^(γ) involving σ_kj_ with |k − j| > k_n_. By noting that $s_{kj}=\sigma_{kj}+O_{p}\left(n^{-1/2}\right)$, we have $\omega^{2}\left(\gamma \right)-\omega^{2}\left(\gamma \right)=o_{p}\left(pn^{-\gamma }\right)$ if k_n_ = o(n^1/2^). Because $\omega^{2}\left(\gamma \right)\sim O\left(pn^{-\gamma }\right)$, we have ω^2^(γ) = (1 + o(1))ω^2^(γ). Similarly, we can derive $\hat{\psi }\left(\gamma \right)=\left(1+o\left(1\right)\psi \left(\gamma \right)\right)$. Under our motivating gene-environmental interaction problems, the genetic variants are weakly dependent, and the dependent structure is often local. In other words, the α-mixing assumption holds and the banding method of Bickel and Levina (2008) works.

On the other hand, when the structure of the covariance matrix Σ is unknown, applying the banding method of Bickel and Levina (2008) might be problematic. As an alternative, we propose a parametric bootstrap-based method to estimate ψ(γ), ω^2^(γ) and R(Γ′) = (ρ_st_), which circumvents the challenging problem of estimating the covariance matrix Σ. Specifically, we first fit a penalized regression model under H_0_ to obtain ${\hat{\mu }}_{0i}$ and then simulate a new set of responses $Y_{i}^{\left(b\right)}$ based on ${\hat{\mu }}_{0i}$ for b=1,2,…,B. Second, we refit the model with the same tuning parameters and calculate the corresponding score vector U^(b)^ and null statistics $L{\left(\gamma \right)}^{\left(b\right)}=\sum_{j=1}^{p}{\left(\frac{1}{n}\sum_{i=1}^{n}U_{ij}^{\left(b\right)}\right)}^{\gamma }$. In practice, we only need to repeat the above procedures for a relatively small B (say, 100) times. Then we estimate ψ(γ), ω^2^(γ) and $\text{R}\left(\Gamma^{\prime }\right)=\left(\rho_{st}\right)$ by $\hat{\psi }\left(\gamma \right)=\sum_{b=1}^{B}L{\left(\gamma \right)}^{\left(b\right)}/B$, ${\hat{\omega }}^{2}\left(\gamma \right)=\sum_{b=1}^{B}{\left(L{\left(\gamma \right)}^{\left(b\right)}-\hat{\psi }\left(\gamma \right)\right)}^{2}/\left(B-1\right)$ and $\hat{R}\left(\Gamma^{\prime }\right)=cor\left(L{\left(\Gamma^{\prime }\right)}^{\left(b\right)}\right)$, where cor is the sample correlation estimated by cor() function in R.

Second, we calculate the *p*-value for aiSPU. Because $\mathrm{cov}\left[L\left(t,\mu_{0}\right),L\left(s,\mu_{0}\right)\right]=o\left(pn^{-\left(t+s\right)/2}\right)$ if *s* + *t* is odd (Theorem 1 part one), iSPU with even *γ* and odd *γ* are asymptotically un- correlated. By Theorem 1 part one, iSPU with finite *γ*s converge jointly and weakly to a multivariate normal distribution as *n, p* → ∞, leading to iSPU with even *γ* and odd *γ* are asymptotically independent. Then by Theorem 1 part three, iSPU with even *γ*, odd *γ*, *γ* = ∞ are asymptotically independent to each other. Because for a finite *γ*, *L*(*γ*) − *ψ*(*γ*)*/ω*(*γ*) follows a standard normal distribution, taking the minimum *p*-values as test statistics equals to taking the maximum of |*L*(*γ*) − *ψ*(*γ*)|*/ω*(*γ*) as test statistics. Then we can analytically calculate the *p*-value for aiSPU by the following two steps. First, define $t_{O}=\max_{\text{odd}\;\;\gamma \in \Gamma }\left|\left(L\left(\gamma \right)-\psi \left(\gamma \right)\right)\right|/\omega \left(\gamma \right)$ and $t_{E}=\max_{\text{even}\;\;\gamma \in \Gamma }\left(L\left(\gamma \right)-\psi \left(\gamma \right)\right)/\omega \left(\gamma \right)$ as the observed test statistics from the data and calculate the p-values for *t*_*O*_ and *t*_*E*_ as $p_{O}=Pr\left[\max_{\text{odd}\;\;\gamma \in \Gamma }\left|\left(L\left(\gamma \right)-\psi \left(\gamma \right)\right)/\omega \left(\gamma \right)\right|>t_{O}\right]$ and $p_{E}=Pr\left[\max_{\text{even}\;\;\gamma \in \Gamma }\left(L\left(\gamma \right)-\psi \left(\gamma \right)\right)/\omega \left(\gamma \right)>t_{E}\right]$. We use *pmvnorm()* in R package *mvrnorm* to calculate the normal tail probabilities of *p*_*O*_ and *p*_*E*_. Further, let *p*_∞_ denote the *p*-value of iSPU(∞), which can be calculated by a extreme value distribution (Theorem 1 part 2). Second, take the minimum *p*-value from the above three categories, that is, *p*_min_ = min{*p*_*O*_*, p*_*E*_*, p*_∞_}. By the asymptotic independence, the asymptotic p-value for the aiSPU test is *p*_aiSPU_ = 1 − (1 − *p*_min_)^3^.

### Parametric bootstrap-based method

We can calculate *p*-values by parametric bootstrap as follows: first, we fit a penalized regression model under *H*_0_ to obtain ${\hat{\mu }}_{0i}$ and then simulate a new set of responses $Y_{i}^{\left(b\right)}$ based on ${\hat{\mu }}_{0i}$ for b=1,2,…,B; second, we refit the model with the same tuning parameters and calculate the corresponding score vector *U* ^(*b*)^ and null statistics $L{\left(\gamma \right)}^{\left(b\right)}=\sum_{j=1}^{p}{\left(\frac{1}{n}\sum_{i=1}^{n}U_{ij}^{\left(b\right)}\right)}^{\gamma }$; third, the *p*-value of iSPU(*γ*) is $P_{L\left(\gamma \right)}=\left[1+\sum_{b=1}^{B}I\left(\left|L{\left(\gamma \right)}^{\left(b\right)}\right|\geq \left|L\left(\gamma \right)\right|\right)\right]/\left(B+1\right)$ and the *p*-value for aiSPU, $P_{\text{aiSPU}}=\left[1+\sum_{b=1}^{B}I\left(T_{\text{aiSPU}}^{\left(b\right)}\leq T_{\text{aiSPU}}\right)\right]/\left(B+1\right)$ with $T_{\text{aiSPU}}^{\left(b\right)}=\min_{\gamma \in \Gamma }P_{L\left(\gamma \right)}^{\left(b\right)}$ and $P_{L\left(\gamma \right)}^{\left(b_{1}\right)}=\left[\sum_{b\neq b_{1}}I\left(\left|L{\left(\gamma \right)}^{\left(b\right)}\right|\geq \left|L{\left(\gamma \right)}^{\left(b_{1}\right)}\right|\right)\right]/B$.

In practice, selecting a good *B* is important for saving computational sources. Here, we start with a smaller *B*, say *B* = 1000 to scan all the tests and then repeatedly increase *B* for the tests that that pass the following criterion: *p*-value *<* 5*/B* in the previous step (Pan et al., 2014). Of note, the accuracy is bounded by the number of bootstraps *B* and calculating a very small *p*-value requires a very large *B*. This is different from asymptotics-based method, in which we only use a relatively small number of bootstraps (say, *B* = 100) to estimate mean, variance, and covariance matrix of iSPU and calculate the *p*-values by Theorem 1.

## Appendix C. Proof of Theorem 1

We prove each part in Theorem 1 separately.

(i) Similar to the proof of Theorem 1 in Wu et al. (2019), we can show that if the conditional mean of *Y*, *µ*_0_, is known, Theorem 1 holds. Specifically, by assumption C4, the order of double summation (across *j*_1_ and *j*_2_) of terms involving $\sigma_{j_{1}j_{2}}$ is *O*(*p*). Then by similar techniques used in Wu et al. (2019), we can calculate *ψ*(*γ*), *ω*(*γ*), and R(Γ′) as shown in Appendix B. We can further use Bernstein’s block idea (Ibragimov and Linnik, 1971) to prove iSPU with finite *γ*s follows a multivariate normal distribution asymptotically. Of note, in our previous work (Wu et al., 2019), we derive a similar Theorem under the *α*-mixing assumption, which is a special case of Theorem 1.

Then for any fixed and finite *γ*, we prove Theorem 1 holds by showing that the difference between *L*(*γ, µ*_0_) and $L\left(\gamma ,{\hat{\mu }}_{0}\right)$ with the TLP estimates is negligible.

Note that the Hellinger distance for linear regression is

$$h^{2}\left(\vartheta ,\vartheta^{0}\right)=1-E\left[\exp\left(-\frac{1}{8}{\left\|\mathbb{Z}\vartheta -\mathbb{Z}\vartheta^{0}\right\|}^{2}\right)\right]$$

and for logistic regression is

$$h^{2}\left(\vartheta ,\vartheta^{0}\right)=\frac{1}{2}E\left[\nu^{1/2}\left({\left(\vartheta^{0}\right)}^{\prime }\mathbb{Z}\right)-\nu^{1/2}\left(\vartheta^{\prime }\mathbb{Z}\right)+{\left(1-\nu \left({\left(\vartheta^{0}\right)}^{\prime }\mathbb{Z}\right)\right)}^{1/2}-{\left(1-\nu \left(\vartheta^{\prime }\mathbb{Z}\right)\right)}^{1/2}\right],$$

where $\nu \left(s\right)={\left(1+\exp\left(s\right)\right)}^{-1}$. We decompose A≡{j:1≤j≤q} into two parts:$A=A^{\tau^{+}}\cup A^{\tau^{-}}$, where $A^{\tau^{+}}\equiv \left\{j:\left|\vartheta_{j}\right|\geq \tau \right\}$ and $A^{\tau^{-}}\equiv \left\{j:\left|\vartheta_{j}\right|<\tau \right\}$. Further note that $\left|\frac{\partial h^{2}\left(\vartheta ,\vartheta^{0}\right)}{\partial \vartheta_{j}}\right|\leq 1/2E\left[\left|Z_{j}\right|\right]$ for 1 ≤ *j* ≤ *q* and $\vartheta \in R^{q}$. Then

$$\left|h^{2}\left(\vartheta ,\vartheta^{0}\right)-h^{2}\left(\vartheta_{\tau^{+}},\vartheta^{0}\right)\right|\leq \tau \left|\sum_{j\in A^{\tau^{-}}}\frac{\partial h^{2}\left(\vartheta ,\vartheta^{0}\right)}{\partial \vartheta_{j}}\right|\leq 2\tau \sum_{j\in A^{\tau^{-}}}E\left[\left|Z_{j}\right|\right]\leq 2\tau q\underset{j}{\max}{\text{Σ}}_{jj},$$

where $\vartheta_{\tau^{+}}=\left(\vartheta_{1}I\left(\left|\vartheta_{1}\right|\geq \tau \right),\ldots ,\vartheta_{q}I\left(\left|\vartheta_{q}\right|\geq \tau \right)\right)$. Then by assumption C7*, − log(1 − *x*) *> x* for any 0 *< x <* 1, and the derivative of 1 − exp(−1/8*×*^2^) and (1 + exp(*x*))^−1/2^ are bounded away from zero, the assumption C7 can be validated.

By assumption C5 and C7, through tuning, Theorem 2 in Shen et al. (2012) yields $P\left(\hat{\vartheta }\neq {\hat{\vartheta }}^{o}\right)\leq \exp\left(-cn+2\log\left(q+1\right)+3\right)$, where *c* is some positive constant. Then we can apply Theorem 2 in Shen et al. (2012) and get the feature selection consistency for $\hat{\vartheta }$, that is, $E\left[h^{2}\left(\hat{\vartheta },\vartheta^{0}\right)\right]=E\left[h^{2}\left({\hat{\vartheta }}^{o},\vartheta^{0}\right)\right]=O\left(q_{0}/n\right)\to 0$ as n → ∞. Then by the consistency property of MLE$\left\|{\hat{\vartheta }}^{o}-\vartheta^{0}\right\|=O_{p}\left(q_{0}n^{-1/2}\right)$, we have $\left\|\hat{\vartheta }-\vartheta^{0}\right\|=O_{p}\left(q_{0}n^{-1/2}\right)$.

Using Taylor expansion and the approach in Le Cessie and Van Houwelingen (1991), we have

$$\hat{D}=\left(I_{n}-W\tilde{\mathbb{Z}}{\left\{I\left(\vartheta \right)\right\}}^{-1}{\tilde{\mathbb{Z}}}^{\prime }\right)D+o_{p}\left(n^{-1/2}\right),$$

where $\hat{D}=\left(Y-{\hat{\mu }}_{0}\right)={\left\{Y_{1}-{\hat{\mu }}_{01},\ldots ,Y_{n}-{\hat{\mu }}_{0n}\right\}}^{\prime }$, $\hat{D}=\left(Y-\mu_{0}\right)={\left\{Y_{1}-\mu_{01},\ldots ,Y_{n}-\mu_{0n}\right\}}^{\prime }$, $I_{n}$ is the *n* × *n* identity matrix, W is a diagonal matrix, which is defined as $W=\text{diag}\left\{E\left(\epsilon_{01}^{2}|\mathbb{Z}\right),\ldots ,E\left(\epsilon_{0n}^{2}|\mathbb{Z}\right)\right\}$, $\tilde{\mathbb{Z}}$ contains the variables corresponding to $A_{0}=\left\{j:\vartheta_{j}^{0}\neq 0\right\}$, and I(ϑ) is a *q*_0_ × *q*_0_ matrix given by $I\left(\vartheta \right)={\tilde{\mathbb{Z}}}^{\prime }W\tilde{\mathbb{Z}}$. Since the smaller order term *o*_*p*_(*n*^−1*/*2^) can be ignored, we focus on the leading term in the subsequent analysis. For notation simplicity, further define $B=W\tilde{\mathbb{Z}}{\left\{I\left(\vartheta \right)\right\}}^{-1}{\tilde{\mathbb{Z}}}^{\prime }={\left(b_{ij}\right)}_{n\times n}$. By Cauchy-Schwarz inequality,

$$b_{ij}=W_{ii}{\tilde{Z}}_{i}{\left\{I\left(\vartheta \right)\right\}}^{-1}{\tilde{Z}}_{j}\leq W_{ii}{\left({\tilde{Z}}_{i}{\left\{I\left(\vartheta \right)\right\}}^{-1}{\tilde{Z}}_{i}\right)}^{1/2}{\left({\tilde{Z}}_{j}{\left\{I\left(\vartheta \right)\right\}}^{-1}{\tilde{Z}}_{j}\right)}^{1/2}.$$

By assumption C6, W is uniformly bounded and $\lim\inf n^{-1}\lambda_{\min}\left(I\left(\vartheta \right)\right)>0$. Then by assumption C3, $\tilde{\mathbb{Z}}$ is uniformly bounded, we have ${\tilde{Z}}_{i}{\left\{I\left\{\vartheta \right\}\right\}}^{-1}{\tilde{Z}}_{i}\leq O\left(q_{0}\right)\times \lambda_{\min}{\left(I\left(\vartheta \right)\right)}^{-1}=O\left(q_{0}n^{-1}\right)$. This leads to $b_{ij}=O\left(q_{0}n^{-1}\right)$ uniformly over *i, j*. By linear algebra, we have $\mu_{0i}-{\hat{\mu }}_{0i}=\sum_{l=1}^{n}b_{il}\epsilon_{0l}$ for 1 ≤ *i* ≤ *n*, where $b_{il}=O\left(q_{0}n^{-1}\right)$. To prove the difference between *L*(*γ, µ*_0_) and $L\left(\gamma ,{\hat{\mu }}_{0}\right)$ can be ignored, we discuss two cases: *γ* = 1 and 1 *< γ <* ∞ separately. To simplify the notation, we denote all the constants by *C* which may vary from place to place.

**For** *γ* = 1: we decompose the statistic $L\left(1,{\hat{\mu }}_{0}\right)$ as

$$L\left(1,{\hat{\mu }}_{0}\right)=\frac{1}{n}\sum_{j=1}^{p}\sum_{i=1}^{n}\left(Y_{i}-{\hat{\mu }}_{0i}\right)X_{ij}=\sum_{j=1}^{p}\sum_{i=1}^{n}\frac{1}{n}S_{ij}+\sum_{j=1}^{p}\sum_{i=1}^{n}\frac{\left(\mu_{0i}-{\hat{\mu }}_{0i}\right)X_{ij}}{n}=T_{10}+T_{11}.$$

Under the null hypothesis and proposed assumptions, Theorem 1 in Wu et al. (2019) leads to

$$T_{10}/\omega \left(1\right)\overset{d}{\to }N\left(0,1\right)\;\;\text{as}\;n\to \infty \;\text{and}\;p\to \infty .$$

For *T*_11_, since $\mu_{0i}-{\hat{\mu }}_{0i}=\sum_{l=1}^{n}b_{il}\epsilon_{0l}$, we have

$$\begin{array}{l} E\left[{\left(T_{11}\right)}^{2}\right]=\frac{1}{n^{2}}\sum_{j_{1}=1}^{p}\sum_{j_{2}=1}^{p}\sum_{i_{1}=1}^{n}\sum_{i_{2}=1}^{n}E\left[\left(\mu_{0i_{1}}-{\hat{\mu }}_{0i_{1}}\right)X_{i_{1}j_{1}}\left(\mu_{0i_{2}}-{\hat{\mu }}_{0i_{2}}\right)X_{i_{2}j_{2}}\right]\\ \;\;\;\;\;=\frac{1}{n^{2}}\sum_{j_{1}=1}^{p}\sum_{j_{2}=1}^{p}\sum_{i_{1}=1}^{n}\sum_{i_{2}=1}^{n}E\left[X_{i_{1}j_{1}}X_{i_{2}j_{2}}\sum_{l=1}^{n}\epsilon_{0l}b_{i_{1}l}\sum_{l=1}^{n}\epsilon_{0l}b_{i_{2}l}\right]\\ \;\;\;\;\;=\frac{1}{n^{2}}\sum_{j_{1}=1}^{p}\sum_{j_{2}=1}^{p}\sum_{i_{1}=1}^{n}\sum_{i_{2}=1}^{n}E[X_{i_{1}j_{1}}X_{i_{2}j_{2}}\left(\epsilon_{0i_{1}}b_{i_{1}i_{1}}+\epsilon_{0i_{2}}b_{i_{1}i_{2}}+\sum_{l\neq i_{1},i_{2}}\epsilon_{0l}b_{i_{1}l}\right)\\ \;\;\;\;\;\;\;\;\;\;\;\;\;\times \left(\epsilon_{0i_{1}}b_{i_{2}i_{1}}+\epsilon_{0i_{2}}b_{i_{2}i_{2}}+\sum_{l\neq i_{1},i_{2}}\epsilon_{0l}b_{i_{2}l}\right)]. \end{array}$$

Since *i*_1_ and *i*_2_ are symmetrical, we have

$$\begin{array}{l} \;\;E\left[{\left(T_{11}\right)}^{2}\right]\\ =\frac{C}{n^{2}}\sum_{j_{1},j_{2},i_{1},i_{2}}E\left[X_{i_{1}j_{1}}X_{i_{2}j_{2}}\epsilon_{0i_{1}}^{2}b_{i_{1}i_{1}}b_{i_{2}i_{1}}\right]+\frac{C}{n^{2}}\sum_{j_{1},j_{2},i_{1},i_{2}}E\left[X_{i_{1}j_{1}}X_{i_{2}j_{2}}\epsilon_{0i_{1}}b_{i_{1}i_{1}}\epsilon_{0i_{2}}b_{i_{2}i_{2}}\right]\\ \;\;\;\;+\frac{C}{n^{2}}\sum_{j_{1},j_{2},i_{1},i_{2}}E\left[X_{i_{1}j_{1}}X_{i_{2}j_{2}}\epsilon_{0i_{1}}b_{i_{1}i_{1}}\sum_{l\neq i_{1},i_{2}}\epsilon_{0l}b_{i_{2}l}\right]+\frac{C}{n^{2}}\sum_{j_{1},j_{2},i_{1},i_{2}}E\left[X_{i_{1}j_{1}}X_{i_{2}j_{2}}\sum_{l\neq i_{1},i_{2}}\epsilon_{{\;}_{0l}}^{2}b_{i_{1}l}b_{i_{2}l}\right]\\ =E\left[T_{111}\right]+E\left[T_{112}\right]+E\left[T_{113}\right]+E\left[T_{114}\right]. \end{array}$$

We discuss the order of each term and show that $\left|T_{11}\right|=o_{p}\left(\sqrt{p}n^{-1/2}\right)$ and thus can be ignored. By assumption C3, $\left|E\left[X_{ij}|\tilde{\mathbb{Z}}\right]\right|\neq 0$ only holds for *j* ∈ *P*_0_, then

$$\begin{array}{l} E\left[T_{111}|\tilde{\mathbb{Z}}\right]=\frac{C}{n^{2}}\sum_{j_{1}=1}^{p}\sum_{j_{2}=1}^{p}\sum_{i_{1}=1}^{n}\sum_{i_{2}=1}^{n}E\left[X_{i_{1}j_{1}}X_{i_{2}j_{2}}\epsilon_{0i_{1}}^{2}b_{i_{1}i_{1}}b_{i_{2}i_{1}}|\tilde{\mathbb{Z}}\right]\\ \;\;\;\;\;\;=\frac{C}{n^{2}}\sum_{j_{1}\notin P_{0}}\sum_{j_{2}\notin P_{0}}\sum_{i_{1}=1}^{n}\sum_{i_{2}=1}^{n}E\left[X_{i_{1}j_{1}}X_{i_{2}j_{2}}\epsilon_{0i_{1}}^{2}b_{i_{1}i_{1}}b_{i_{2}i_{1}}|\tilde{\mathbb{Z}}\right]\\ \;\;\;\;\;\;\;\;\;+\frac{C}{n^{2}}\sum_{j_{1}\in P_{0}}\sum_{j_{2}\notin P_{0}}\sum_{i_{1}=1}^{n}\sum_{i_{2}=1}^{n}E\left[X_{i_{1}j_{1}}X_{i_{2}j_{2}}\epsilon_{0i_{1}}^{2}b_{i_{1}i_{1}}b_{i_{2}i_{1}}|\tilde{\mathbb{Z}}\right]\\ \;\;\;\;\;\;\;\;\;+\frac{C}{n^{2}}\sum_{j_{1}\notin P_{0}}\sum_{j_{2}\in P_{0}}\sum_{i_{1}=1}^{n}\sum_{i_{2}=1}^{n}E\left[X_{i_{1}j_{1}}X_{i_{2}j_{2}}\epsilon_{0i_{1}}^{2}b_{i_{1}i_{1}}b_{i_{2}i_{1}}|\tilde{\mathbb{Z}}\right]\\ \;\;\;\;\;\;\;\;\;+\frac{C}{n^{2}}\sum_{j_{1}\in P_{0}}\sum_{j_{2}\in P_{0}}\sum_{i_{1}=1}^{n}\sum_{i_{2}=1}^{n}E\left[X_{i_{1}j_{1}}X_{i_{2}j_{2}}\epsilon_{0i_{1}}^{2}b_{i_{1}i_{1}}b_{i_{2}i_{1}}|\tilde{\mathbb{Z}}\right]. \end{array}$$

For the first term, by Assumptions C3 and C4, we have

$$\begin{array}{l} \;\;\;\;\frac{C}{n^{2}}\sum_{j_{1}\notin P_{0}}\sum_{j_{2}\notin P_{0}}\sum_{i_{1}=1}^{n}\sum_{i_{2}=1}^{n}E\left[X_{i_{1}j_{1}}X_{i_{2}j_{2}}\epsilon_{0i_{1}}^{2}b_{i_{1}i_{1}}b_{i_{2}i_{1}}|\tilde{\mathbb{Z}}\right]\\ =O\left(n^{-2}\right)\sum_{j_{1}\notin P_{0}}\sum_{j_{2}\notin P_{0}}\sum_{i_{1}=1}^{n}E\left[X_{i_{1}j_{1}}X_{i_{1}j_{2}}\epsilon_{0i_{1}}^{2}|\tilde{\mathbb{Z}}\times O\left(q_{0}^{2}n^{-2}\right)\right]\\ =O\left(n^{-2}\right)\times O\left(np^{2}\right)\times O\left(q_{0}^{2}n^{-2}\right)\\ =O\left(pn^{-1}\right)\times O\left(pq_{0}^{2}n^{-2}\right)=o\left(pn^{-1}\right). \end{array}$$

Of note, because *b*_*ij*_ is a function of $\tilde{\mathbb{Z}}$, it can be taken out of the expectation when conditional on $\tilde{\mathbb{Z}}$. For the second term, we have

$$\begin{array}{l} \;\;\;\frac{C}{n^{2}}\sum_{j_{1}\in P_{0}}\sum_{j_{2}\notin P_{0}}\sum_{i_{1}=1}^{n}\sum_{i_{2}=1}^{n}E\left[X_{i_{1}j_{1}}X_{i_{2}j_{2}}\epsilon_{0i_{1}}^{2}b_{i_{1}i_{1}}b_{i_{2}i_{1}}|\tilde{\mathbb{Z}}\right]\\ =O\left(n^{-2}\right)\sum_{j_{1}\in P_{0}}\sum_{j_{2}\notin P_{0}}\sum_{i_{1}=1}^{n}\sum_{i_{2}=1}^{n}E\left[X_{i_{1}j_{1}}X_{i_{2}j_{2}}\epsilon_{0i_{1}}^{2}|\tilde{\mathbb{Z}}\right]\times O\left(q_{0}^{2}n^{-2}\right)\\ =O\left(n^{-2}\right)\times O\left(pp_{0}n^{2}\right)\times O\left(q_{0}^{2}n^{-2}\right)=O\left(pp_{0}q_{0}^{2}n^{-2}\right)=o\left(pn^{-1}\right). \end{array}$$

By noting that $p_{0}=O\left(p^{\eta_{1}}\right)$ for a small positive *η*_1_ and assumption C5$pq_{0}^{4}/n^{2}=o\left(1\right)$, we can derive the last equation. Similar to the derivation of the second term, for the third term, we have

$$\frac{C}{n^{2}}\sum_{j_{1}\notin P_{0}}\sum_{j_{2}\in P_{0}}\sum_{i_{1}=1}^{n}\sum_{i_{2}=1}^{n}E\left[X_{i_{1}j_{1}}X_{i_{2}j_{2}}\epsilon_{0i_{1}}^{2}b_{i_{1}i_{1}}b_{i_{2}i_{1}}|\tilde{\mathbb{Z}}\right]=o\left(pn^{-1}\right).$$

For the last term, we have

$$\begin{array}{l} \;\;\;\;\frac{C}{n^{2}}\sum_{j_{1}\in P_{0}}\sum_{j_{2}\in P_{0}}\sum_{i_{1}=1}^{n}\sum_{i_{2}=1}^{n}E\left[X_{i_{1}j_{1}}X_{i_{2}j_{2}}\epsilon_{0i_{1}}^{2}b_{i_{1}i_{1}}b_{i_{2}i_{1}}|\tilde{\mathbb{Z}}\right]\\ =O\left(n^{-2}\right)\sum_{j_{1}\in P_{0}}\sum_{j_{2}\in P_{0}}\sum_{i_{1}=1}^{n}\sum_{i_{2}=1}^{n}E\left[X_{i_{1}j_{1}}X_{i_{2}j_{2}}\epsilon_{0i_{1}}^{2}|\tilde{\mathbb{Z}}\right]\times O\left(q_{0}^{2}n^{-2}\right)\\ =O\left(n^{-2}\right)\times O\left(p_{0}^{2}n^{2}\right)\times O\left(q_{0}^{2}n^{-2}\right)=O\left(n^{-2}q_{0}^{2}p_{0}^{2}\right)=o\left(pn^{-1}\right). \end{array}$$

By combining the above derivations, we have $E\left[T_{111}|\tilde{\mathbb{Z}}\right]=o\left(pn^{-1}\right)$. Importantly,

$$\sum_{j_{1},j_{2}}\sum_{i_{1},i_{2}}E\left[X_{i_{1}j_{1}}X_{i_{2}j_{2}}\epsilon_{0i_{1}}^{2}|\tilde{\mathbb{Z}}\right]=o\left(pq_{0}^{2}n^{2}\right).$$

Next, we discuss the order of $E\left[T_{112}|\tilde{\mathbb{Z}}\right]$. By noting that $E\left[X_{ij}\epsilon_{0i}|\tilde{\mathbb{Z}}\right]=0$, we have

$$\begin{array}{l} E\left[T_{112}|\tilde{\mathbb{Z}}\right]=\frac{C}{n^{2}}\sum_{j_{1}=1}^{p}\sum_{j_{2}=1}^{p}\sum_{i_{1}=1}^{n}\sum_{i_{2}=1}^{n}E\left[X_{i_{1}j_{1}}X_{i_{2}j_{2}}\epsilon_{0i_{1}}b_{i_{1}i_{1}}\epsilon_{0i_{2}}b_{i_{2}i_{2}}|\tilde{\mathbb{Z}}\right]\\ \;\;\;\;\;\;=O\left(n^{-2}\right)\sum_{j_{1}=1}^{p}\sum_{j_{2}=1}^{p}\sum_{i_{1}=1}^{n}E\left[X_{i_{1}j_{1}}\epsilon_{0i_{1}}^{2}X_{i_{2}j_{2}}|\tilde{\mathbb{Z}}\right]\times O\left(q_{0}^{2}n^{-2}\right)\\ \;\;\;\;\;\;=O\left(n^{-2}\right)\times O\left(p^{2}n\right)\times O\left(q_{0}^{2}n^{-2}\right)=O\left(pn^{-1}pq_{0}^{2}n^{-2}\right)=o\left(pn^{-1}\right). \end{array}$$

Next, we discuss the order of $E\left[T_{113}|\tilde{\mathbb{Z}}\right]$:

$$\begin{array}{l} E\left[T_{113}|\tilde{\mathbb{Z}}\right]=\frac{C}{n^{2}}\sum_{j_{1},j_{2},i_{1},i_{2}}E\left[X_{i_{1}j_{1}}X_{i_{2}j_{2}}\epsilon_{0i_{1}}b_{i_{1}i_{1}}\sum_{l\neq i_{1},i_{2}}\epsilon_{0l}b_{i_{2}l}|\tilde{\mathbb{Z}}\right]\\ =O\left(n^{-2}\right)\sum_{j_{1},j_{2},i_{1},i_{2}}E\left[X_{i_{1}j_{1}}X_{i_{2}j_{2}}\epsilon_{0i_{1}}b_{i_{1}i_{1}}|\tilde{\mathbb{Z}}\right]E\left[\sum_{l\neq i_{1},i_{2}}\epsilon_{0l}b_{i_{2}l}|\tilde{\mathbb{Z}}\right]\\ =O\left(n^{-2}\right)\sum_{j_{1},j_{2},i_{1},i_{2}}E\left[X_{i_{1}j_{1}}X_{i_{2}j_{2}}\epsilon_{0i_{1}}b_{i_{1}i_{1}}|\tilde{\mathbb{Z}}\right]\times 0=0. \end{array}$$

Next, we discuss the order of $E\left[T_{114}|\tilde{\mathbb{Z}}\right]$. Similarly, we decompose $E\left[T_{114}|\tilde{\mathbb{Z}}\right]$ into three parts and bound each part separately.

$$\begin{array}{l} E\left[T_{114}|\tilde{\mathbb{Z}}\right]=\frac{C}{n^{2}}\sum_{j_{1},j_{2},i_{1},i_{2}}E\left[X_{i_{1}j_{1}}X_{i_{2}j_{2}}\sum_{l\neq i_{1},i_{2}}\epsilon_{0l}^{2}b_{i_{1}l}b_{i_{2}l}|\tilde{\mathbb{Z}}\right]\\ \;\;\;\;\;\;=O\left(n^{-2}\right)\sum_{j_{1},j_{2}}\sum_{i_{1},i_{2}}E\left[X_{i_{1}j_{1}}X_{i_{2}j_{2}}|\tilde{\mathbb{Z}}\right]E\left[\sum_{l\neq i_{1},i_{2}}\epsilon_{0l}^{2}b_{i_{1}l}b_{i_{2}l}|\tilde{\mathbb{Z}}\right]\\ \;\;\;\;\;\;=O\left(n^{-2}\right)\sum_{j_{1},j_{2}}\sum_{i_{1},i_{2}}E\left[X_{i_{1}j_{1}}X_{i_{2}j_{2}}|\tilde{\mathbb{Z}}\right]\times O\left(q_{0}^{2}n^{-1}\right)\\ \;\;\;\;\;\;=O\left(n^{-2}\right)\sum_{j_{1}\notin P_{0},j_{2}\notin P_{0}}\sum_{i_{1},i_{2}}E\left[X_{i_{1}j_{1}}X_{i_{2}j_{2}}|\tilde{\mathbb{Z}}\right]\times O\left(q_{0}^{2}n^{-1}\right)\\ \;\;\;\;\;\;+O\left(n^{-2}\right)\sum_{j_{1}\notin P_{0},j_{2}\in P_{0}}\sum_{i_{1},i_{2}}E\left[X_{i_{1}j_{1}}X_{i_{2}j_{2}}|\tilde{\mathbb{Z}}\right]\times O\left(q_{0}^{2}n^{-1}\right)\\ \;\;\;\;\;\;+O\left(n^{-2}\right)\sum_{j_{1}\in P_{0},j_{2}\in P_{0}}\sum_{i_{1},i_{2}}E\left[X_{i_{1}j_{1}}X_{i_{2}j_{2}}|\tilde{\mathbb{Z}}\right]\times O\left(q_{0}^{2}n^{-1}\right). \end{array}$$

By assumption C3, $E\left(X_{1j}|\tilde{\mathbb{Z}}\right)=0$ for $j\notin P_{0}$. Then by assumption C4, for the first part, we have

$$\begin{array}{l} \;\;\;O\left(n^{-2}\right)\sum_{j_{1}\notin P_{0},j_{2}\notin P_{0}}\sum_{i_{1},i_{2}}E\left[X_{i_{1}j_{1}}X_{i_{2}j_{2}}|\tilde{\mathbb{Z}}\right]\times O\left(q_{0}^{2}n^{-1}\right)\\ =O\left(q_{0}^{2}n^{-3}\right)\sum_{i_{1}=1}^{n}\sum_{j_{1}\notin P_{0},j_{2}\notin P_{0}}E\left[X_{i_{1}j_{1}}X_{i_{1}j_{2}}|\tilde{\mathbb{Z}}\right]\\ =O\left(q_{0}^{2}n^{-3}\right)\times O\left(pn\right)\\ =O\left(pn^{-1}n^{-1}q_{0}^{2}\right)=o\left(pn^{-1}\right). \end{array}$$

Similarly, we have the following for the second part:

$$\begin{array}{l} \;\;\;O\left(n^{-2}\right)\sum_{j_{1}\notin P_{0},j_{2}\in P_{0}}\sum_{i_{1},i_{2}}E\left[X_{i_{1}j_{1}}X_{i_{2}j_{2}}|\tilde{\mathbb{Z}}\right]\times O\left(q_{0}^{2}n^{-1}\right)\\ =O\left(q_{0}^{2}n^{-3}\right)\sum_{j_{1}\notin P_{0},j_{2}\in P_{0}}\sum_{i_{1}=1}^{n}E\left[X_{i_{1}j_{1}}X_{i_{2}j_{2}}|\tilde{\mathbb{Z}}\right]\\ =O\left(q_{0}^{2}n^{-3}\right)\times O\left(p_{0}pn\right)\\ =O\left(pn^{-1}p_{0}q_{0}^{2}n^{-1}\right)=o\left(pn^{-1}\right). \end{array}$$

Note that by assumptions C3 and C5, $p_{0}=O\left(p^{\eta_{1}}\right)$ for a small positive *η*_1_ and $q_{0}=O\left(n^{\eta_{2}}\right)$ for a small positive *η*_2_. Then $O\left(p_{0}q_{0}^{2}n^{-1}\right)=O\left(p^{\eta_{1}}n^{1-2\eta_{2}}\right)=o\left(1\right)$ and the above last equation holds.

For the last part, we have

$$\begin{array}{l} \;\;\;O\left(n^{-2}\right)\sum_{j_{1}\in P_{0},j_{2}\in P_{0}}\sum_{i_{1},i_{2}}E\left[X_{i_{1}j_{1}}X_{i_{2}j_{2}}|\tilde{\mathbb{Z}}\right]\times O\left(q_{0}^{2}n^{-1}\right)\\ =O\left(n^{-2}\right)\times O\left(p_{0}^{2}n^{2}\right)\times O\left(q_{0}^{2}n^{-1}\right)=O\left(q_{0}^{2}p_{0}^{2}n^{-1}\right)=o\left(pn^{-1}\right). \end{array}$$

By Combining the above equations, we have $E\left[T_{114}|\tilde{\mathbb{Z}}\right]=o\left(pn^{-1}\right)$. Importantly, we have

$$\sum_{j_{1},j_{2}}\sum_{i_{1},i_{2}}E\left[X_{i_{1}j_{1}}X_{i_{2}j_{2}}|\tilde{\mathbb{Z}}\right]=o\left(pn^{2}q_{0}^{-2}\right).$$

In summary, we have $E\left[{\left(T_{11}\right)}^{2}\right]=E\left[E\left[T_{111}|\tilde{\mathbb{Z}}\right]+E\left[T_{112}|\tilde{\mathbb{Z}}\right]+E\left[T_{113}|\tilde{\mathbb{Z}}\right]+E\left[T_{113}|\tilde{\mathbb{Z}}\right]\right]=o\left(pn^{-1}\right)$, leading to $\left|T_{11}\right|=o_{p}\left(n^{-1/2}\sqrt{p}\right)$. Thus *T*_11_ can be ignored and this completes the proof for *γ* = 1.

**For** 1 *< γ <* ∞: we decompose the statistic $L\left(\gamma ,{\hat{\mu }}_{0}\right)$ as

$$\begin{array}{l} L\left(\gamma ,{\hat{\mu }}_{0}\right)=\sum_{j=1}^{p}{\left(\frac{1}{n}\sum_{i=1}^{n}\left(Y_{i}-{\hat{\mu }}_{0i}\right)X_{ij}\right)}^{\gamma }\\ \;\;\;\;\;\;=\sum_{j=1}^{p}{\left(\frac{1}{n}\sum_{i=1}^{n}\left(\left(Y_{i}-\mu_{0i}\right)+\left(\mu_{0i}-{\hat{\mu }}_{0i}\right)\right)X_{ij}\right)}^{\gamma }\\ \;\;\;\;\;\;=\sum_{j=1}^{p}{\left(\frac{1}{n}\sum_{i=1}^{n}S_{ij}\right)}^{\gamma }+\sum_{1\leq v\leq \gamma }\left(\begin{array}{l} \gamma \\ v \end{array}\right)\sum_{j=1}^{p}{\left(\frac{1}{n}\sum_{i=1}^{n}S_{ij}\right)}^{\gamma -v}{\left(\frac{1}{n}\sum_{i=1}^{n}\left(\mu_{0i}-{\hat{\mu }}_{0i}\right)X_{ij}\right)}^{v}\\ \;\;\;\;\;\;=T_{\gamma 0}+\sum_{v=1}^{\gamma }T_{\gamma v},\;\;\;\;\;\text{say}. \end{array}$$

Under the null hypothesis and proposed assumptions, we have $\left\{T_{\gamma 0}-\psi \left(\gamma \right)\right\}/\omega \left(\gamma \right)\overset{d}{\to }N\left(0,1\right)$ as *n, p* → ∞. Then we discuss two cases: *v* = 1 and *v >* 1 separately for the orders of *T*_*γv*_, 1 ≤ *v* ≤ *γ*.

When *v* = 1, we have

$$\begin{array}{l} E\left[{\left(T_{\gamma 1}\right)}^{2}\right]\\ =E\left[\frac{C}{n^{2}}\sum_{j_{1}}^{p}\sum_{j_{2}}^{p}{\left(\sum_{i=1}^{n}\frac{1}{n}S_{ij_{1}}\right)}^{\gamma -1}{\left(\sum_{i=1}^{n}\frac{1}{n}S_{ij_{2}}\right)}^{\gamma -1}\sum_{i=1}^{n}\left(\left(\mu_{0i}-{\hat{\mu }}_{0i}\right)X_{ij_{1}}\right)\sum_{i=1}^{n}\left(\left(\mu_{0i}-{\hat{\mu }}_{0i}\right)X_{ij_{2}}\right)\right]\\ =\frac{C}{n^{2}}\sum_{j_{1},j_{2}}\sum_{i_{1},i_{2},i_{3},i_{4}}E[X_{i_{1}j_{1}}X_{i_{2}j_{2}}\epsilon_{0i_{3}}b_{i_{1}i_{3}}\epsilon_{0i_{4}}b_{i_{2}i_{4}}\times {\left(\sum_{l\in \left\{i_{1},i_{2},i_{3},i_{4}\right\}}\frac{\epsilon_{0l}X_{lj_{1}}}{n}+\sum_{l\notin \left\{i_{1},i_{2},i_{3},i_{4}\right\}}\frac{\epsilon_{0l}X_{lj_{1}}}{n}\right)}^{\gamma -1}\\ \;\;\;\;\;\;\times {\left(\sum_{l\in \left\{i_{1},i_{2},i_{3},i_{4}\right\}}\frac{\epsilon_{0l}X_{lj_{2}}}{n}+\sum_{l\notin \left\{i_{1},i_{2},i_{3},i_{4}\right\}}\frac{\epsilon_{0l}X_{lj_{2}}}{n}\right)}^{\gamma -1}]. \end{array}$$

By Binomial theorem, we have

$$\begin{array}{l} {\left(\sum_{l}\frac{\epsilon_{0l}X_{lj_{1}}}{n}\right)}^{\gamma -1}\leq {\left(\sum_{l\notin \left\{i_{1},\ldots ,i_{4}\right\}}\frac{\epsilon_{0l}X_{lj_{1}}}{n}\right)}^{\gamma -1}+C\sum_{l\in \left\{i_{1},\ldots ,i_{4}\right\}}\frac{\epsilon_{0l}X_{lj_{1}}}{n}{\left(\sum_{l\notin \left\{i_{1},\ldots ,i_{4}\right\}}\frac{\epsilon_{0l}X_{lj_{1}}}{n}\right)}^{\gamma -2}+\ldots \\ \;\;\;\;\;\;\;\;+C{\left(\sum_{l\in \left\{i_{1},\ldots ,i_{4}\right\}}\frac{\epsilon_{0l}X_{lj_{1}}}{n}\right)}^{\gamma -2}\sum_{l\notin \left\{i_{1},\ldots ,i_{4}\right\}}\frac{\epsilon_{0l}X_{lj_{1}}}{n})+C{\left(\sum_{l\in \left\{i_{1},\ldots ,i_{4}\right\}}\frac{\epsilon_{0l}X_{lj_{1}}}{n}\right)}^{\gamma -1}. \end{array}$$

Then

$$\begin{array}{l} E\left[{\left(T_{\gamma 1}\right)}^{2}\right]=\sum_{k_{1}=1}^{\gamma }\sum_{k_{2}=1}^{\gamma }\frac{C}{n^{2}}\sum_{j_{1},j_{2}}\sum_{i_{1},i_{2},i_{3},i_{4}}E[X_{i_{1}j_{1}}X_{i_{2}j_{2}}\epsilon_{0i_{3}}b_{i_{1}i_{3}}\epsilon_{0i_{4}}b_{i_{2}i_{4}}{\left(\sum_{l\in \left\{i_{1},\ldots ,i_{4}\right\}}\frac{\epsilon_{0l}X_{lj_{1}}}{n}\right)}^{k_{1}-1}\\ \;\;\;\;\;\;\;\;\;\;\times {\left(\sum_{l\in \left\{i_{1},\ldots ,i_{4}\right\}}\frac{\epsilon_{0l}X_{lj_{2}}}{n}\right)}^{k_{2}-1}{\left(\sum_{l\notin \left\{i_{1},i_{2},i_{3},i_{4}\right\}}\frac{\epsilon_{0l}X_{lj_{1}}}{n}\right)}^{\gamma -k_{1}}{\left(\sum_{l\notin \left\{i_{1},i_{2},i_{3},i_{4}\right\}}\frac{\epsilon_{0l}X_{lj_{2}}}{n}\right)}^{\gamma -k_{2}}]\\ \;\;\;\;\;\;=\sum_{k_{1}=1}^{\gamma }\sum_{k_{2}=1}^{\gamma }T_{\gamma 1k_{1}k_{2}},\;\;\;\;\;\text{say}. \end{array}$$

To prove the order of |*T*_*γ*1_| is ignorable, we discuss two situations: *k*_1_+*k*_2_ ≤ 6 and *k*_1_+*k*_2_ *>* 6. First, we focus on the situation with *k*_1_+*k*_2_ ≤ 6 and discuss the order of each term separately. For *T*_*γ*111_, we have

$$\begin{array}{l} \;\;\;T_{\gamma 111}\\ =\frac{C}{n^{2}}\sum_{j_{1},j_{2}}\sum_{i_{1},i_{2},i_{3},i_{4}}E\left[X_{i_{1}j_{1}}X_{i_{2}j_{2}}\epsilon_{0i_{3}}b_{i_{1}i_{3}}\epsilon_{0i_{4}}b_{i_{2}i_{4}}{\left(\sum_{l\notin \left\{i_{1},i_{2},i_{3},i_{4}\right\}}\frac{\epsilon_{0l}X_{lj_{1}}}{n}\right)}^{\gamma -1}{\left(\sum_{l\notin \left\{i_{1},i_{2},i_{3},i_{4}\right\}}\frac{\epsilon_{0l}X_{lj_{2}}}{n}\right)}^{\gamma -1}\right]\\ =\frac{C}{n^{2}}\sum_{j_{1},j_{2}}\sum_{i_{1},i_{2},i_{3},i_{4}}E\left[X_{i_{1}j_{1}}X_{i_{2}j_{2}}\epsilon_{0i_{3}}b_{i_{1}i_{3}}\epsilon_{0i_{4}}b_{i_{2}i_{4}}{\left(\sum_{l\notin \left\{i_{1},\ldots ,i_{4}\right\}}\frac{\epsilon_{0l}X_{lj_{1}}}{n}\right)}^{\gamma -1}{\left(\sum_{l\notin \left\{i_{1},\ldots ,i_{4}\right\}}\frac{\epsilon_{0l}X_{lj_{2}}}{n}\right)}^{\gamma -1}\right]\\ =O\left(n^{-2}\right)\sum_{j_{1},j_{2}}\sum_{i_{1},i_{2},i_{3},i_{4}}E\left[X_{i_{1}j_{1}}X_{i_{2}j_{2}}\epsilon_{0i_{3}}b_{i_{1}i_{3}}\epsilon_{0i_{4}}b_{i_{2}i_{4}}\right]\times O\left(n^{-\left(\gamma -1\right)}\right)\\ =O\left(n^{-2}\right)\sum_{j_{1},j_{2}}\sum_{i_{1},i_{2},i_{3},i_{4}}E\left[X_{i_{1}j_{1}}X_{i_{2}j_{2}}\epsilon_{0i_{3}}b_{i_{1}i_{3}}\epsilon_{0i_{4}}b_{i_{2}i_{4}}\right]\times O\left(n^{-\left(\gamma -1\right)}\right). \end{array}$$

Note that

$$\begin{array}{l} \;\;\;\sum_{j_{1},j_{2}}\sum_{i_{1},i_{2},i_{3},i_{4}}E\left[X_{i_{1}j_{1}}X_{i_{2}j_{2}}\epsilon_{0i_{3}}b_{i_{1}i_{3}}\epsilon_{0i_{4}}b_{i_{2}i_{4}}|\tilde{\mathbb{Z}}\right]\times O\left(n^{-\left(\gamma -1\right)}\right)\\ =\sum_{j_{1},j_{2}}\sum_{i_{1},i_{2},i_{3},i_{4}}E\left[X_{i_{1}j_{1}}X_{i_{2}j_{2}}\epsilon_{0i_{3}}\epsilon_{0i_{4}}|\tilde{\mathbb{Z}}\right]\times O\left(q_{0}^{2}n^{-\left(\gamma +1\right)}\right)\\ =\sum_{j_{1},j_{2}}\sum_{i_{1},i_{2},i_{3}}E\left[X_{i_{1}j_{1}}X_{i_{2}j_{2}}\epsilon_{0i_{3}}^{2}|\tilde{\mathbb{Z}}\right]\times O\left(q_{0}^{2}n^{-\left(\gamma +\right)}\right)\\ \;\;\;+\sum_{j_{1},j_{2}}\sum_{i_{1},i_{2}}E\left[X_{i_{1}j_{1}}\epsilon_{0i_{1}}X_{i_{2}j_{2}}\epsilon_{0i_{2}}|\tilde{\mathbb{Z}}\right]\times O\left(q_{0}^{2}n^{-\left(\gamma +1\right)}\right)\\ \;\;\;+\sum_{j_{1},j_{2}}\sum_{i_{1},i_{2}}E\left[X_{i_{1}j_{1}}\epsilon_{0i_{1}}^{2}X_{i_{2}j_{2}}|\tilde{\mathbb{Z}}\right]\times O\left(q_{0}^{2}n^{-\left(\gamma +1\right)}\right). \end{array}$$

We discuss each term separately. By a similar discussion of $E\left[T_{114}|\tilde{\mathbb{Z}}\right]$, for the first term in *T*_*γ*111_, we have

$$\begin{array}{l} \;\;\;\sum_{j_{1},j_{2}}\sum_{i_{1},i_{2},i_{3}}E\left[X_{i_{1}j_{1}}X_{i_{2}j_{2}}\epsilon_{0i_{3}}^{2}|\tilde{\mathbb{Z}}\right]\times O\left(q_{0}^{2}n^{-\left(\gamma +1\right)}\right)\\ =\sum_{j_{1},j_{2}}\sum_{i_{1},i_{2}}E\left[X_{i_{1}j_{1}}X_{i_{2}j_{2}}|\tilde{\mathbb{Z}}\right]\times O\left(q_{0}^{2}n^{-\gamma }\right)\\ =o\left(pn^{2}q_{0}^{-2}\right)\times O\left(q_{0}^{2}n^{-\gamma }\right)=o\left(pn^{-\gamma +2}\right). \end{array}$$

For the second term in *T*_*γ*111_, by noting that $E\left[X_{ij}\epsilon_{0i}|\mathbb{Z}\right]=0$, we have

$$\begin{array}{l} \;\;\;\sum_{j_{1},j_{2}}\sum_{i_{1},i_{2}}E\left[X_{i_{1}j_{1}}\epsilon_{0i_{1}}X_{i_{2}j_{2}}\epsilon_{0i_{2}}|\tilde{\mathbb{Z}}\right]\times O\left(q_{0}^{2}n^{-\left(\gamma +1\right)}\right)\\ =\sum_{j_{1},j_{2}}\sum_{i_{1}}E\left[X_{i_{1}j_{1}}X_{i_{2}j_{2}}\epsilon_{0i_{1}}^{2}|\tilde{\mathbb{Z}}\right]\times O\left(q_{0}^{2}n^{-\left(\gamma +1\right)}\right)\\ =O\left(p^{2}n\right)\times O\left(q_{0}^{2}n^{-\left(\gamma +1\right)}\right)=O\left(q_{0}^{2}p^{2}n^{-\gamma }\right)=o\left(pn^{-\gamma +2}\right). \end{array}$$

For the third term in *T*_*γ*111_, similar to the discussion of *T*_111_, we have

$$\begin{array}{l} \;\;\;\sum_{j_{1},j_{2}}\sum_{i_{1},i_{2}}E\left[X_{i_{1}j_{1}}\epsilon_{0i_{1}}^{2}X_{i_{2}j_{2}}|\tilde{\mathbb{Z}}\right]\times O\left(q_{0}^{2}n^{-\left(\gamma +1\right)}\right)\\ =o\left(pn^{2}q_{0}^{-2}\right)\times O\left(q_{0}^{2}n^{-\left(\gamma +1\right)}\right)=o\left(pn^{-\gamma +1}\right)=o\left(pn^{-\gamma +2}\right). \end{array}$$

By combing the above three equations, we have *T*_*γ*111_ = *o*(*pn*^−*γ*^).

Similarly,

$$\begin{array}{l} T_{\gamma 121}\\ =\frac{C}{n^{2}}\sum_{j_{1},j_{2}}\sum_{i_{1},\ldots ,i_{4}}E[X_{i_{1}j_{1}}X_{i_{2}j_{2}}\epsilon_{0i_{3}}b_{i_{1}i_{3}}\epsilon_{0i_{4}}b_{i_{2}i_{4}}\sum_{l\in \left\{i_{1},\ldots ,i_{4}\right\}}\frac{\epsilon_{0l}X_{lj_{1}}}{n}\\ \;\;\;\;\;\;\;\;\;\times {\left(\sum_{l\notin \left\{i_{1},\ldots ,i_{4}\right\}}\frac{\epsilon_{0l}X_{lj_{1}}}{n}\right)}^{\gamma -2}{\left(\sum_{l\notin \left\{i_{1},\ldots ,i_{4}\right\}}\frac{\epsilon_{0l}X_{lj_{2}}}{n}\right)}^{\gamma -1}]\\ =\frac{C}{n^{2}}\sum_{j_{1},j_{2}}\sum_{i_{1},\ldots ,i_{4}}E\left[X_{i_{1}j_{1}}X_{i_{2}j_{2}}\epsilon_{0i_{3}}b_{i_{1}i_{3}}\epsilon_{0i_{4}}b_{i_{2}i_{4}}\sum_{l\in \left\{i_{1},\ldots ,i_{4}\right\}}\frac{\epsilon_{0l}X_{lj_{1}}}{n}\right]\\ \;\;\;\;\;\;\;\;\;\times E\left[{\left(\sum_{l\notin \left\{i_{1},\ldots ,i_{4}\right\}}\frac{\epsilon_{0l}X_{lj_{1}}}{n}\right)}^{\gamma -2}{\left(\sum_{l\notin \left\{i_{1},\ldots ,i_{4}\right\}}\frac{\epsilon_{0l}X_{lj_{2}}}{n}\right)}^{\gamma -1}\right]\\ =\frac{C}{n^{2}}\sum_{j_{1},j_{2}}\sum_{i_{1},i_{2},i_{3}}E\left[X_{i_{1}j_{1}}^{2}X_{i_{2}j_{2}}\epsilon_{0i_{1}}b_{i_{1}i_{3}}b_{i_{2}i_{3}}\epsilon_{0i_{3}}^{2}+X_{i_{1}j_{1}}X_{i_{2}j_{2}}b_{i_{1}i_{3}}b_{i_{2}i_{3}}\epsilon_{0i_{3}}^{3}X_{i_{3}j_{1}}\right]\times O\left(n^{-\left(\gamma -1\right)}\right)\\ =O\left(n^{-\gamma -2}\right)\sum_{j_{1},j_{2}}\sum_{i_{1},i_{2},i_{3}}\left(E\left[X_{i_{1}j_{1}}^{2}X_{i_{2}j_{2}}\epsilon_{0i_{1}}b_{i_{1}i_{3}}b_{i_{2}i_{3}}\epsilon_{0i_{3}}^{2}\right]+E\left[X_{i_{1}j_{1}}X_{i_{2}j_{2}}b_{i_{1}i_{3}}b_{i_{2}i_{3}}\epsilon_{0i_{3}}^{3}X_{i_{3}j_{1}}\right]\right). \end{array}$$

Again, we discuss each term separately. Note that since $E\left[\epsilon |X,\tilde{\mathbb{Z}}\right]=0$, we have $E\left[X_{ij}^{2}\epsilon_{0i}|\tilde{\mathbb{Z}}\right]=0$. Thus

$$\begin{array}{l} \;\;\;\;\sum_{j_{1},j_{2}}\sum_{i_{1},i_{2},i_{3}}E\left[X_{i_{1}j_{1}}^{2}X_{i_{2}j_{2}}\epsilon_{0i_{1}}b_{i_{1}i_{3}}b_{i_{2}i_{3}}\epsilon_{0i_{3}}^{2}|\tilde{\mathbb{Z}}\right]\times O\left(n^{-\gamma -2}\right)\\ =\sum_{j_{1},j_{2}}\sum_{i_{1},i_{2}}E\left[X_{i_{1}j_{1}}^{2}X_{i_{2}j_{2}}\epsilon_{0i_{1}}^{3}|\tilde{\mathbb{Z}}\right]\times O\left(q_{0}^{2}n^{-\gamma -4}\right)\\ =O\left(p^{2}n^{2}\right)\times O\left(q_{0}^{2}n^{-\gamma -4}\right)=O\left(pn^{-\gamma }pq_{0}^{2}n^{-2}\right)=o\left(pn^{-\gamma }\right). \end{array}$$

Similarly, for the second term in *T*_*γ*121_, we have

$$\begin{array}{l} \;\;\;\sum_{j_{1},j_{2}}\sum_{i_{1},i_{2},i_{3}}E\left[X_{i_{1}j_{1}}X_{i_{2}j_{2}}b_{i_{1}i_{3}}b_{i_{2}i_{3}}\epsilon_{0i_{3}}^{3}X_{i_{3}j_{1}}|\tilde{\mathbb{Z}}\right]\times O\left(n^{-\gamma -2}\right)\\ =\sum_{j_{1},j_{2}}\sum_{i_{1},i_{2},i_{3}}E\left[X_{i_{1}j_{1}}X_{i_{2}j_{2}}|\tilde{\mathbb{Z}}\right]E\left[\epsilon_{0i_{3}}^{3}X_{i_{3}j_{1}}|\tilde{\mathbb{Z}}\right]\times O\left(q_{0}^{2}n^{-\gamma -4}\right)\\ =\sum_{j_{1},j_{2}}\sum_{i_{1},i_{2}}E\left[X_{i_{1}j_{1}}X_{i_{2}j_{2}}|\tilde{\mathbb{Z}}\right]\times O\left(q_{0}^{2}n^{-\gamma -3}\right)\\ =o\left(pn^{2}q_{0}^{-2}\right)\times O\left(q_{0}^{2}n^{-\gamma -3}\right)=o\left(pn^{-\gamma }\right). \end{array}$$

Combining the above two equations, we have *T*_*γ*121_ = *o*(*pn*^−*γ*^).

For *T*_*γ*122_, we have

$$\begin{array}{l} T_{\gamma 122}\\ =\frac{C}{n^{2}}\sum_{j_{1},j_{2}}\sum_{i_{1},\ldots ,i_{4}}E[X_{i_{1}j_{1}}X_{i_{2}j_{2}}\epsilon_{0i_{3}}b_{i_{1}i_{3}}\epsilon_{0i_{4}}b_{i_{2}i_{4}}\sum_{l\in \left\{i_{1},\ldots ,i_{4}\right\}}\frac{\epsilon_{0l}X_{lj_{1}}}{n}\sum_{l\in \left\{i_{1},\ldots ,i_{4}\right\}}\frac{\epsilon_{0l}X_{lj_{2}}}{n}\\ \;\;\;\;\;\;\;\;\;\times {\left(\sum_{l\notin \left\{i_{1},\ldots ,i_{4}\right\}}\frac{\epsilon_{0l}X_{lj_{1}}}{n}\right)}^{\gamma -2}{\left(\sum_{l\notin \left\{i_{1},\ldots ,i_{4}\right\}}\frac{\epsilon_{0l}X_{lj_{2}}}{n}\right)}^{\gamma -2}]\\ =\frac{C}{n^{2}}\sum_{j_{1},j_{2}}\sum_{i_{1},\ldots ,i_{4}}E\left[X_{i_{1}j_{1}}X_{i_{2}j_{2}}\epsilon_{0i_{3}}b_{i_{1}i_{3}}\epsilon_{0i_{4}}b_{i_{2}i_{4}}\sum_{l\in \left\{i_{1},\ldots ,i_{4}\right\}}\frac{\epsilon_{0l}X_{lj_{1}}}{n}\sum_{l\in \left\{i_{1},\ldots ,i_{4}\right\}}\frac{\epsilon_{0l}X_{lj_{2}}}{n}\right]\\ \;\;\;\;\;\;\;\;\;\times E\left[{\left(\sum_{l\notin \left\{i_{1},\ldots ,i_{4}\right\}}\frac{\epsilon_{0l}X_{lj_{1}}}{n}\right)}^{\gamma -2}{\left(\sum_{l\notin \left\{i_{1},\ldots ,i_{4}\right\}}\frac{\epsilon_{0l}X_{lj_{2}}}{n}\right)}^{\gamma -2}\right]\\ =\frac{C}{n^{2}}\sum_{j_{1},j_{2}}\sum_{i_{1},\ldots ,i_{4}}E\left[X_{i_{1}j_{1}}X_{i_{2}j_{2}}\epsilon_{0i_{3}}b_{i_{1}i_{3}}\epsilon_{0i_{4}}b_{i_{2}i_{4}}\sum_{l\in \left\{i_{1},\ldots ,i_{4}\right\}}\epsilon_{0l}X_{lj_{1}}\sum_{l\in \left\{i_{1},\ldots ,i_{4}\right\}}\epsilon_{0l}X_{lj_{2}}\right]\times O\left(n^{-\gamma +2}\right). \end{array}$$

Note that

$$\begin{array}{l} \;\;\;\sum_{j_{1},j_{2}}\sum_{i_{1},\ldots ,i_{4}}E\left[X_{i_{1}j_{1}}X_{i_{2}j_{2}}\epsilon_{0i_{3}}b_{i_{1}i_{3}}\epsilon_{0i_{4}}b_{i_{2}i_{4}}\sum_{l\in \left\{i_{1},\ldots ,i_{4}\right\}}\epsilon_{0l}X_{lj_{1}}\sum_{l\in \left\{i_{1},\ldots ,i_{4}\right\}}\epsilon_{0l}X_{lj_{2}}|\tilde{\mathbb{Z}}\right]\times O\left(n^{-\gamma +2}\right)\\ =\sum_{j_{1},j_{2}}\sum_{i_{1},i_{2}}E\left[X_{i_{1}j_{1}}^{2}X_{i_{2}j_{2}}^{2}\epsilon_{0i_{1}}^{2}\epsilon_{0i_{2}}^{2}+X_{i_{1}j_{1}}^{2}X_{i_{1}j_{2}}X_{i_{2}j_{2}}\epsilon_{0i_{1}}^{4}|\tilde{\mathbb{Z}}\right]\times O\left(q_{0}^{2}n^{-\gamma }\right)\\ \;\;\;+\sum_{j_{1},j_{2}}\sum_{i_{1},i_{2},i_{3}}E\left[X_{i_{1}j_{1}}^{2}\epsilon_{0i_{1}}X_{i_{2}j_{2}}^{2}\epsilon_{0i_{2}}\epsilon_{0i_{3}}^{2}|\tilde{\mathbb{Z}}\right]\times O\left(q_{0}^{2}n^{-\gamma }\right)\\ \;\;\;+\sum_{j_{1},j_{2}}\sum_{i_{1},i_{2},i_{3}}E\left[X_{i_{1}j_{1}}^{2}X_{i_{1}j_{2}}\epsilon_{0i_{1}}^{2}X_{i_{2}j_{2}}\epsilon_{0i_{3}}^{2}|\tilde{\mathbb{Z}}\right]\times O\left(q_{0}^{2}n^{-\gamma }\right)\\ \;\;\;+\sum_{j_{1},j_{2}}\sum_{i_{1},i_{2},i_{3}}E\left[X_{i_{1}j_{1}}X_{i_{2}j_{2}}\epsilon_{0i_{3}}^{4}X_{i_{3}j_{1}}X_{i_{3}j_{2}}|\tilde{\mathbb{Z}}\right]\times O\left(q_{0}^{2}n^{-\gamma }\right)\\ \;\;\;+\sum_{j_{1},j_{2}}\sum_{i_{1},i_{2},i_{3},i_{4}}E\left[X_{i_{1}j_{1}}X_{i_{2}j_{2}}X_{i_{3}j_{1}}\epsilon_{0i_{3}}^{2}X_{i_{4}j_{2}}\epsilon_{0i_{4}}^{2}|\tilde{\mathbb{Z}}\right]\times O\left(q_{0}^{2}n^{-\gamma }\right). \end{array}$$

Then we discuss each term separately. For the first term, we have

$$\begin{array}{l} \;\;\;\sum_{j_{1},j_{2}}\sum_{i_{1},i_{2}}E\left[X_{i_{1}j_{1}}^{2}X_{i_{2}j_{2}}^{2}\epsilon_{0i_{1}}^{2}\epsilon_{0i_{2}}^{2}+X_{i_{1}j_{1}}^{2}X_{i_{1}j_{2}}X_{i_{2}j_{2}}\epsilon_{0i_{1}}^{4}|\tilde{\mathbb{Z}}\right]\times O\left(q_{0}^{2}n^{-\gamma }\right)\\ =O\left(p^{2}n^{2}\right)\times O\left(q_{0}^{2}n^{-\gamma }\right)=o\left(pn^{-\gamma +4}\right). \end{array}$$

For the second term, by noting that $E\left[X_{ij}^{2}\epsilon_{0i}|\tilde{\mathbb{Z}}\right]=0$, we have

$$\begin{array}{l} \;\;\;\sum_{j_{1},j_{2}}\sum_{i_{1},i_{2},i_{3}}E\left[X_{i_{1}j_{1}}^{2}\epsilon_{0i_{1}}X_{i_{2}j_{2}}^{2}\epsilon_{0i_{2}}\epsilon_{0i_{3}}^{2}|\tilde{\mathbb{Z}}\right]\times O\left(q_{0}^{2}n^{-\gamma }\right)\\ =\sum_{j_{1},j_{2}}\sum_{i_{1},i_{2},i_{3}}E\left[X_{i_{1}j_{1}}^{2}\epsilon_{0i_{1}}X_{i_{2}j_{2}}^{2}\epsilon_{0i_{2}}|\tilde{\mathbb{Z}}\right]\times E\left[\epsilon_{0i_{3}}^{2}|\tilde{\mathbb{Z}}\right]\times O\left(q_{0}^{2}n^{-\gamma }\right)\\ =\sum_{j_{1},j_{2}}\sum_{i_{1},i_{3}}E\left[X_{i_{1}j_{1}}^{2}\epsilon_{0i_{1}}^{2}X_{i_{1}j_{2}}^{2}|\tilde{\mathbb{Z}}\right]\times E\left[\epsilon_{0i_{3}}^{2}|\tilde{\mathbb{Z}}\right]\times O\left(q_{0}^{2}n^{-\gamma }\right)\\ =O\left(p^{2}n\right)\times O\left(n\right)\times O\left(q_{0}^{2}n^{-\gamma }\right)=o\left(pn^{-\gamma +4}\right). \end{array}$$

For the third term, we have

$$\begin{array}{l} \;\;\;\sum_{j_{1},j_{2}}\sum_{i_{1},i_{2},i_{3}}E\left[X_{i_{1}j_{1}}^{2}X_{i_{1}j_{2}}\epsilon_{0i_{1}}^{2}X_{i_{2}j_{2}}\epsilon_{0i_{3}}^{2}|\tilde{\mathbb{Z}}\right]\times O\left(q_{0}^{2}n^{-\gamma }\right)\\ =\sum_{j_{1},j_{2}}\sum_{i_{1},i_{2}}E\left[X_{i_{1}j_{1}}^{2}X_{i_{1}j_{2}}\epsilon_{0i_{1}}^{2}X_{i_{2}j_{2}}|\tilde{\mathbb{Z}}\right]\times \sum_{i_{3}}E\left[\epsilon_{0i_{3}}^{2}|\tilde{\mathbb{Z}}\right]\times O\left(q_{0}^{2}n^{-\gamma }\right)\\ =\sum_{j_{1},j_{2}}\sum_{i_{1},i_{2}}E\left[X_{i_{1}j_{1}}^{2}X_{i_{1}j_{2}}\epsilon_{0i_{1}}^{2}X_{i_{2}j_{2}}|\tilde{\mathbb{Z}}\right]\times O\left(q_{0}^{2}n^{-\gamma +1}\right)\\ =\sum_{j_{1},j_{2}}\sum_{i_{1}\neq i_{2}}E\left[X_{i_{1}j_{1}}^{2}X_{i_{1}j_{2}}\epsilon_{0i_{1}}^{2}X_{i_{2}j_{2}}|\tilde{\mathbb{Z}}\right]\times O\left(q_{0}^{2}n^{-\gamma +1}\right)+\sum_{j_{1},j_{2}}\sum_{i_{1}}E\left[X_{i_{1}j_{1}}^{2}X_{i_{1}j_{2}}^{2}\epsilon_{0i_{1}}^{2}|\tilde{\mathbb{Z}}\right]\times O\left(q_{0}^{2}n^{-\gamma +1}\right). \end{array}$$

We discuss each term separately as follows. First,

$$\begin{array}{l} \;\;\;\sum_{j_{1},j_{2}}\sum_{i_{1}\neq i_{2}}E\left[X_{i_{1}j_{1}}^{2}X_{i_{1}j_{2}}\epsilon_{0i_{1}}^{2}X_{i_{2}j_{2}}|\tilde{\mathbb{Z}}\right]\times O\left(q_{0}^{2}n^{-\gamma +1}\right)\\ =\sum_{i_{1}\neq i_{2}}\sum_{j_{1}\notin P_{0},j_{2}\notin P_{0}}E\left[X_{i_{1}j_{1}}^{2}X_{i_{1}j_{2}}\epsilon_{0i_{1}}^{2}|\tilde{\mathbb{Z}}\right]\times E\left[X_{i_{2}j_{2}}|\tilde{\mathbb{Z}}\right]\times O\left(q_{0}^{2}n^{-\gamma +1}\right)\\ \;+\sum_{i_{1}\neq i_{2}}\sum_{j_{1}\notin P_{0},j_{2}\in P_{0}}E\left[X_{i_{1}j_{1}}^{2}X_{i_{1}j_{2}}\epsilon_{0i_{1}}^{2}|\tilde{\mathbb{Z}}\right]\times E\left[X_{i_{2}j_{2}}|\tilde{\mathbb{Z}}\right]\times O\left(q_{0}^{2}n^{-\gamma +1}\right)\\ \;+\sum_{i_{1}\neq i_{2}}\sum_{j_{1}\in P_{0},j_{2}\notin P_{0}}E\left[X_{i_{1}j_{1}}^{2}X_{i_{1}j_{2}}\epsilon_{0i_{1}}^{2}|\tilde{\mathbb{Z}}\right]\times E\left[X_{i_{2}j_{2}}|\tilde{\mathbb{Z}}\right]\times O\left(q_{0}^{2}n^{-\gamma +1}\right)\\ \;+\sum_{i_{1}\neq i_{2}}\sum_{j_{1}\in P_{0},j_{2}\in P_{0}}E\left[X_{i_{1}j_{1}}^{2}X_{i_{1}j_{2}}\epsilon_{0i_{1}}^{2}|\tilde{\mathbb{Z}}\right]\times E\left[X_{i_{2}j_{2}}|\tilde{\mathbb{Z}}\right]\times O\left(q_{0}^{2}n^{-\gamma +1}\right) \end{array}$$

By noting that $E\left[X_{i_{2}j_{2}}|\tilde{\mathbb{Z}}\right]=0$, we have

$$\begin{array}{l} \;\;\;\sum_{j_{1},j_{2}}\sum_{i_{1}\neq i_{2}}E\left[X_{i_{1}j_{1}}^{2}X_{i_{1}j_{2}}\epsilon_{0i_{1}}^{2}X_{i_{2}j_{2}}|\tilde{\mathbb{Z}}\right]\times O\left(q_{0}^{2}n^{-\gamma +1}\right)\\ =\sum_{i_{1}\neq i_{2}}\sum_{j_{1}\notin P_{0},j_{2}\in P_{0}}E\left[X_{i_{1}j_{1}}^{2}X_{i_{1}j_{2}}\epsilon_{0i_{1}}^{2}|\tilde{\mathbb{Z}}\right]\times E\left[X_{i_{2}j_{2}}|\tilde{\mathbb{Z}}\right]\times O\left(q_{0}^{2}n^{-\gamma +1}\right)\\ \;\;\;+\sum_{i_{1}\neq i_{2}}\sum_{j_{1}\in P_{0},j_{2}\in P_{0}}E\left[X_{i_{1}j_{1}}^{2}X_{i_{1}j_{2}}\epsilon_{0i_{1}}^{2}|\tilde{\mathbb{Z}}\right]\times E\left[X_{i_{2}j_{2}}|\tilde{\mathbb{Z}}\right]\times O\left(q_{0}^{2}n^{-\gamma +1}\right)\\ =\left[O\left(n^{2}pp_{0}\right)+\left[O\left(n^{2}p_{0}^{2}\right)\right]\times O\left(q_{0}^{2}n^{-\gamma +1}\right)\right]\\ =O\left(pn^{-\gamma +4}\right)\times \left[O\left(p_{0}q_{0}^{2}n^{-1}\right)+O\left(p_{0}^{2}p^{-1}q_{0}^{2}n^{-1}\right)\right]\\ =o\left(pn^{-\gamma +4}\right). \end{array}$$

According to assumptions C3 and C4, $p_{0}=O\left(p^{n_{1}}\right)$ for a small positive *η*_1_ and $q_{0}=O\left(n^{\eta_{2}}\right)$ for a small positive *η*_2_. Then $p^{1/2-\eta_{3}/2}q_{0}^{2}n^{-1}=o\left(n^{-1}\right)$ and we can derive the last equation accordingly.

Second, by the discussion of *T*_111_, we have

$$\begin{array}{l} \;\sum_{j_{1},j_{2}}\sum_{i_{1}}E\left[X_{i_{1}j_{1}}^{2}X_{i_{1}j_{2}}^{2}\epsilon_{0i_{1}}^{2}|\tilde{\mathbb{Z}}\right]\times O\left(q_{0}^{2}n^{-\gamma +1}\right)\\ =O\left(p^{2}n\right)\times O\left(q_{0}^{2}n^{-\gamma +1}\right)=O\left(pn^{-\gamma +4}\right)\times O\left(pq_{0}^{2}n^{-2}\right)=o\left(pn^{-\gamma +4}\right). \end{array}$$

For the fourth term, similar to the derivation of *E*[*T*_114_|Z˜], we have

$$\begin{array}{l} \;\;\;\sum_{j_{1},j_{2}}\sum_{i_{1},i_{2},i_{3}}E\left[X_{i_{1}j_{1}}X_{i_{2}j_{2}}\epsilon_{0i_{3}}^{4}X_{i_{3}j_{1}}X_{i_{3}j_{2}}|\tilde{\mathbb{Z}}\right]\times O\left(q_{0}^{2}n^{-\gamma }\right)\\ =\sum_{j_{1},j_{2}}\sum_{i_{1},i_{2},i_{3}}E\left[X_{i_{1}j_{1}}X_{i_{2}j_{2}}|\tilde{\mathbb{Z}}\right]E\left[\epsilon_{0i_{3}}^{4}X_{i_{3}j_{1}}X_{i_{3}j_{2}}|\tilde{\mathbb{Z}}\right]\times O\left(q_{0}^{2}n^{-\gamma }\right)\\ =\sum_{j_{1},j_{2}}\sum_{i_{1},i_{2}}E\left[X_{i_{1}j_{1}}X_{i_{2}j_{2}}|\tilde{\mathbb{Z}}\right]\times O(n)\times O\left(q_{0}^{2}n^{-\gamma }\right)\\ =o\left(pn^{2}q_{0}^{-2}\right)\times O\left(q_{0}^{2}n^{-\gamma +1}\right)=o\left(pn^{-\gamma +4}\right). \end{array}$$

For the last term, by the derivation of $E\left[T_{114}|\tilde{\mathbb{Z}}\right]$, we have

$$\begin{array}{l} \;\;\;\sum_{j_{1},j_{2}}\sum_{i_{1},i_{2},i_{3},i_{4}}E\left[X_{i_{1}j_{1}}X_{i_{2}j_{2}}X_{i_{3}j_{1}}\epsilon_{0i_{3}}^{2}X_{i_{4}j_{2}}\epsilon_{0i_{4}}^{2}|\tilde{\mathbb{Z}}\right]\times O\left(q_{0}^{2}n^{-\gamma }\right)\\ =\sum_{j_{1},j_{2}}\sum_{i_{1},i_{2}}E\left[X_{i_{1}j_{1}}X_{i_{2}j_{2}}|\tilde{\mathbb{Z}}\right]\times O\left(n^{2}\right)\times O\left(q_{0}^{2}n^{-\gamma }\right)\\ =\sum_{j_{1},j_{2}}\sum_{i_{1},i_{2}}E\left[X_{i_{1}j_{1}}X_{i_{2}j_{2}}|\tilde{\mathbb{Z}}\right]=o\left(pn^{2}q_{0}^{-2}\right)\times O\left(q_{0}^{2}n^{-\gamma +2}\right)=o\left(pn^{-\gamma +4}\right). \end{array}$$

Combining the above equations, we have *T*_*γ*122_ = *o*(*pn*^−*γ*^).

Then we discuss the order of *T*_*γ*131_, we have

$$\begin{array}{l} T_{\gamma 122}=\frac{C}{n^{2}}\sum_{j_{1},j_{2}}\sum_{i_{1},\ldots ,i_{4}}E[X_{i_{1}j_{1}}X_{i_{2}j_{2}}\epsilon_{0i_{3}}b_{i_{1}i_{3}}\epsilon_{0i_{4}}b_{i_{2}i_{4}}{\left(\sum_{l\in \left\{i_{1},\ldots ,i_{4}\right\}}\frac{\epsilon_{0l}X_{lj_{1}}}{n}\right)}^{2}\\ \;\;\;\;\;\;\;\;\;\times {\left(\sum_{l\notin \left\{i_{1},\ldots ,i_{4}\right\}}\frac{\epsilon_{0l}X_{lj_{1}}}{n}\right)}^{\gamma -3}{\left(\sum_{l\notin \left\{i_{1},\ldots ,i_{4}\right\}}\frac{\epsilon_{0l}X_{lj_{2}}}{n}\right)}^{\gamma -1}]\\ \;\;\;\;\;=O\left(n^{-2}\right)\sum_{j_{1},j_{2}}\sum_{i_{1},\ldots ,i_{4}}E\left[X_{i_{1}j_{1}}X_{i_{2}j_{2}}\epsilon_{0i_{3}}b_{i_{1}i_{3}}\epsilon_{0i_{4}}b_{i_{2}i_{4}}{\left(\sum_{l\in \left\{i_{1},\ldots ,i_{4}\right\}}\frac{\epsilon_{0l}X_{lj_{1}}}{n}\right)}^{2}\right]\\ \;\;\;\;\;\;\;\;\;\times E\left[{\left(\sum_{l\notin \left\{i_{1},\ldots ,i_{4}\right\}}\frac{\epsilon_{0l}X_{lj_{1}}}{n}\right)}^{\gamma -3}{\left(\sum_{l\notin \left\{i_{1},\ldots ,i_{4}\right\}}\frac{\epsilon_{0l}X_{lj_{2}}}{n}\right)}^{\gamma -1}\right]\\ \;\;\;\;\;=O\left(n^{-2}\right)\sum_{j_{1},j_{2}}\sum_{i_{1},\ldots ,i_{4}}E\left[X_{i_{1}j_{1}}X_{i_{2}j_{2}}\epsilon_{0i_{3}}b_{i_{1}i_{3}}\epsilon_{0i_{4}}b_{i_{2}i_{4}}{\left(\sum_{l\in \left\{i_{1},\ldots ,i_{4}\right\}}\frac{\epsilon_{0l}X_{lj_{1}}}{n}\right)}^{2}\right]\times O\left(n^{-\gamma +2}\right). \end{array}$$

${\left(\sum_{l\in \left\{i_{1},\ldots ,i_{4}\right\}}\epsilon_{0l}X_{lj_{1}}/n\right)}^{2}$ and $\left(\sum_{l\in \left\{i_{1},\ldots ,i_{4}\right\}}\epsilon_{0l}X_{lj_{1}}/n\right)\times \left(\sum_{l\in \left\{i_{1},\ldots ,i_{4}\right\}}\epsilon_{0l}X_{lj_{2}}/n\right)$ have the same effect to the order. Similar to the discussion of *T*_*γ*122_, we have *T*_*γ*131_ = *o*(*pn*^−*γ*^). Next, we discuss the order of *T*_*γ*132_:

$$\begin{array}{l} \;\;\;T_{\gamma 132}\\ =\frac{C}{n^{2}}\sum_{j_{1},j_{2}}\sum_{i_{1},\ldots ,i_{4}}E[X_{i_{1}j_{1}}X_{i_{2}j_{2}}\epsilon_{0i_{3}}b_{i_{1}i_{3}}\epsilon_{0i_{4}}b_{i_{2}i_{4}}{\left(\sum_{l\in \left\{i_{1},\ldots ,i_{4}\right\}}\frac{\epsilon_{0l}X_{lj_{1}}}{n}\right)}^{2}\sum_{l\in \left\{i_{1},\ldots ,i_{4}\right\}}\frac{\epsilon_{0l}X_{lj_{2}}}{n}\\ \;\;\;\;\;\;\;\;\;\times {\left(\sum_{l\notin \left\{i_{1},\ldots ,i_{4}\right\}}\frac{\epsilon_{0l}X_{lj_{1}}}{n}\right)}^{\gamma -3}{\left(\sum_{l\notin \left\{i_{1},\ldots ,i_{4}\right\}}\frac{\epsilon_{0l}X_{lj_{2}}}{n}\right)}^{\gamma -2}]\\ \;\;\;\;\;=O\left(n^{-\gamma -3}\right)\sum_{j_{1},j_{2}}\sum_{i_{1},\ldots ,i_{4}}E\left[X_{i_{1}j_{1}}X_{i_{2}j_{2}}\epsilon_{0i_{3}}b_{i_{1}i_{3}}\epsilon_{0i_{4}}b_{i_{2}i_{4}}{\left(\sum_{l\in \left\{i_{1},\ldots ,i_{4}\right\}}\epsilon_{0l}X_{lj_{1}}\right)}^{2}\sum_{l\in \left\{i_{1},\ldots ,i_{4}\right\}}\epsilon_{0l}X_{lj_{2}}\right]. \end{array}$$

For a fixed *j*_1_ and *j*_2_,

$$\sum_{j_{1},j_{2}}\sum_{i_{1},\ldots ,i_{4}}E\left[X_{i_{1}j_{1}}X_{i_{2}j_{2}}\epsilon_{0i_{3}}b_{i_{1}i_{3}}\epsilon_{0i_{4}}b_{i_{2}i_{4}}{\left(\sum_{i\in \left\{i_{1},\ldots ,i_{4}\right\}}\epsilon_{0l}X_{lj_{1}}\right)}^{2}\sum_{i\in \left\{i_{1},\ldots ,i_{4}\right\}}\epsilon_{0l}X_{lj_{2}}\right]$$

contains 5 $\epsilon_{0i}$ and $E\left[\epsilon_{0i}|X,\tilde{\mathbb{Z}}\right]=0$. Then it at most contains *n*^2^ terms with non-zero expectation. Since *b*_*ij*_ = *O*(*q*_0_*n*^−1^), we have

$$T_{\gamma 132}=O\left(n^{-\gamma -3}\right)\times O\left(p^{2}n^{2}q_{0}^{2}n^{-2}\right)=O\left(p^{2}q_{0}^{2}n^{-\gamma -3}\right)=O\left(pn^{-\gamma }\right)\times O\left(pq_{0}^{2}n^{-3}\right)=o\left(pn^{-\gamma }\right).$$

Similarly, we can prove *T*_*γ*141_ = *o*(*pn*^−*γ*^). For *T*_*γ*133_, we have

$$\begin{array}{l} \;\;\;T_{\gamma 133}\\ =\frac{C}{n^{2}}\sum_{j_{1},j_{2}}\sum_{i_{1},\ldots ,i_{4}}E[X_{i_{1}j_{1}}X_{i_{2}j_{2}}\epsilon_{0i_{3}}b_{i_{1}i_{3}}\epsilon_{0i_{4}}b_{i_{2}i_{4}}{\left(\sum_{l\in \left\{i_{1},\ldots ,i_{4}\right\}}\frac{\epsilon_{0l}X_{lj_{1}}}{n}\right)}^{2}{\left(\sum_{l\in \left\{i_{1},\ldots ,i_{4}\right\}}\frac{\epsilon_{0l}X_{lj_{2}}}{n}\right)}^{2}\\ \;\;\;\;\;\;\;\;\;\times {\left(\sum_{l\notin \left\{i_{1},\ldots ,i_{4}\right\}}\frac{\epsilon_{0l}X_{lj_{1}}}{n}\right)}^{\gamma -3}{\left(\sum_{l\notin \left\{i_{1},\ldots ,i_{4}\right\}}\frac{\epsilon_{0l}X_{lj_{2}}}{n}\right)}^{\gamma -3}]\\ \;\;\;\;\;=O\left(n^{-\gamma -3}\right)\sum_{j_{1},j_{2}}\sum_{i_{1},\ldots ,i_{4}}E\left[X_{i_{1}j_{1}}X_{i_{2}j_{2}}\epsilon_{0i_{3}}b_{i_{1}i_{3}}\epsilon_{0i_{4}}b_{i_{2}i_{4}}{\left(\sum_{l\in \left\{i_{1},\ldots ,i_{4}\right\}}\epsilon_{0l}X_{lj_{1}}\right)}^{2}{\left(\sum_{l\in \left\{i_{1},\ldots ,i_{4}\right\}}\epsilon_{0l}X_{lj_{2}}\right)}^{2}\right]. \end{array}$$

Since $\sum_{i_{1},\ldots ,i_{4}}E\left[X_{i_{1}j_{1}}X_{i_{2}j_{2}}\epsilon_{0i_{3}}b_{i_{1}i_{3}}\epsilon_{0i_{4}}b_{i_{2}i_{4}}{\left(\sum_{l\in \left\{i_{1},\ldots ,i_{4}\right\}}\epsilon_{0l}X_{lj_{1}}\right)}^{2}{\left(\sum_{l\in \left\{i_{1},\ldots ,i_{4}\right\}}\epsilon_{0l}X_{lj_{2}}\right)}^{2}\right]$ contains 6 $\epsilon_{0i}$ and $E\left[\epsilon_{0i}|X,\tilde{\mathbb{Z}}\right]=0$, for a fixed *j*_1_ and *j*_2_, it at most contains *n*^3^ terms with non-zero expectation. Thus

$$T_{\gamma 133}=O\left(n^{-\gamma -3}\right)\times O\left(p^{2}n^{3}q_{0}^{2}n^{-2}\right)=O\left(q_{0}^{2}p^{2}n^{-\gamma -2}\right)=O\left(pn^{-\gamma }\right)\times O\left(pq_{0}^{2}n^{-2}\right)=o\left(pn^{-\gamma }\right).$$

Similarly, we can prove $T_{\gamma 1k_{1}k_{2}}=o\left(pn^{-\gamma }\right)$ for *k*_1_ + *k*_2_ = 6.

For *k*_1_ + *k*_2_ ≥ 7, we have

$$\begin{array}{l} \;\;\;\frac{C}{n^{2}}\sum_{j_{1},j_{2}}\sum_{i_{1},\ldots ,i_{4}}E[X_{i_{1}j_{1}}X_{i_{2}j_{2}}\epsilon_{0i_{3}}b_{i_{1}i_{3}}\epsilon_{0i_{4}}b_{i_{2}i_{4}}\times {\left(\sum_{l\in \left\{i_{1},\ldots ,i_{4}\right\}}\frac{\epsilon_{0l}X_{lj_{1}}}{n}\right)}^{{\;}^{k_{1}-1}}\\ \;\;\;\;\times {\left(\sum_{l\in \left\{i_{1},\ldots ,i_{4}\right\}}\frac{\epsilon_{0l}X_{lj_{2}}}{n}\right)}^{k_{2}-1}{\left(\sum_{l\notin \left\{i_{1},\ldots ,i_{4}\right\}}\frac{\epsilon_{0l}X_{lj_{1}}}{n}\right)}^{\gamma -k_{1}}{\left(\sum_{l\notin \left\{i_{1},\ldots ,i_{4}\right\}}\frac{\epsilon_{0l}X_{lj_{2}}}{n}\right)}^{\gamma -k_{2}}]\\ =O\left(n^{-2}\right)\sum_{j_{1},j_{2}}\sum_{i_{1},\ldots ,i_{4}}E\left[X_{i_{1}j_{1}}X_{i_{2}j_{2}}\epsilon_{0i_{3}}b_{i_{1}i_{3}}\epsilon_{0i_{4}}b_{i_{2}i_{4}}{\left(\sum_{l\in \left\{i_{1},\ldots ,i_{4}\right\}}\frac{\epsilon_{0l}X_{lj_{1}}}{n}\right)}^{k_{1}-1}{\left(\sum_{l\in \left\{i_{1},\ldots ,i_{4}\right\}}\frac{\epsilon_{0l}X_{lj_{2}}}{n}\right)}^{k_{2}-1}\right]\\ \;\;\;\;\times E\left[{\left(\sum_{l\notin \left\{i_{1},\ldots ,i_{4}\right\}}\frac{\epsilon_{0l}X_{lj_{1}}}{n}\right)}^{\gamma -k_{1}}{\left(\sum_{l\notin \left\{i_{1},\ldots ,i_{4}\right\}}\frac{\epsilon_{0l}X_{lj_{2}}}{n}\right)}^{\gamma -k_{2}}\right]\\ =O\left(n^{-2}\right)\sum_{j_{1},j_{2}}\sum_{i_{1},\ldots ,i_{4}}E\left[X_{i_{1}j_{1}}X_{i_{2}j_{2}}\epsilon_{0i_{3}}b_{i_{1}i_{3}}\epsilon_{0i_{4}}b_{i_{2}i_{4}}{\left(\sum_{l\in \left\{i_{1},\ldots ,i_{4}\right\}}\frac{\epsilon_{0l}X_{lj_{1}}}{n}\right)}^{{\;}^{k_{1}-1}}{\left(\sum_{l\in \left\{i_{1},\ldots ,i_{4}\right\}}\frac{\epsilon_{0l}X_{lj_{2}}}{n}\right)}^{{\;}^{k_{2}-1}}\right]\\ \;\;\;\;\times O\left(n^{-\gamma +\left\lfloor \left(k_{1}+k_{2}\right)/2\right\rfloor }\right)\\ =O\left(n^{-2}\right)O\left(p^{2}\times n^{4}\times q_{0}^{2}n^{-2}\times n^{-\left(k_{1}+k_{2}-2\right)}\right)\times O\left(n^{-\gamma +\left\lfloor \left(k_{1}+k_{2}\right)/2\right\rfloor }\right)\\ =O\left(pn^{-\gamma }\right)\times O\left(q_{0}^{2}pn^{-\left(k_{1}+k_{2}-2\right)+\left\lfloor \left(k_{1}+k_{2}\right)/2\right\rfloor }\right)=o\left(pn^{-\gamma }\right). \end{array}$$

By noting that $pq_{0}^{2}=o\left(n^{2}\right)$ and for *k*_1_ + *k*_2_ ≥ 7, −(*k*_1_ + *k*_2_ − 2) +$\left\lfloor \left(k_{1}+k_{2}\right)/2\right\rfloor \geq 2$, we can derive the last equation. In summary, we have $\left|T_{\gamma 1}\right|=o_{p}\left(n^{-\gamma /2}\sqrt{p}\right)$.

Next, we discuss 1 *< v* ≤ *γ*.

By Minkowski’s inequality, we have

$$E\left\|T_{\gamma v}\right\|\leq C\sum_{j=1}^{p}E\left[\left|{\left(\frac{1}{n}\sum_{i=1}^{n}S_{ij}\right)}^{\gamma -v}{\left(\frac{1}{n}\sum_{i=1}^{n}\left(\mu_{0i}-{\hat{\mu }}_{0i}\right)X_{ij}\right)}^{v}\right|\right].$$

Then by Cauchy-Schwarz inequality,

$$\begin{array}{l} E\left[\left|T_{\gamma v}\right|\right]\leq C\sum_{j=1}^{p}E{\left[{\left(\frac{1}{n}\sum_{i=1}^{n}S_{ij}\right)}^{2\left(\gamma -v\right)}\right]}^{1/2}E{\left[{\left(\frac{1}{n}\sum_{i=1}^{n}\left(\mu_{0i}-{\hat{\mu }}_{0i}\right)X_{ij}\right)}^{2v}\right]}^{1/2}\\ \;\;\;\;\;\;\;\;\;\;\;\;\leq O\left(n^{-\left(\gamma -v\right)/2}\right)\sum_{j=1}^{p}E{\left[{\left(\frac{1}{n}\sum_{i=1}^{n}\left(\mu_{0i}-{\hat{\mu }}_{0i}\right)X_{ij}\right)}^{2v}\right]}^{1/2}. \end{array}$$

Next, we derive the order of $T_{2v,j}={\left(\frac{1}{n}\sum_{i=1}^{n}\left(\mu_{0i}-{\hat{\mu }}_{0i}\right)X_{ij}\right)}^{2v}$ for any positive integer *v*. For *j* ∈ *P*_0_, we have

$$\begin{array}{l} E\left[T_{2v,j}|\tilde{\mathbb{Z}}\right]=\frac{1}{n^{2v}}\sum_{i_{1},i_{2},\ldots ,i_{4v}}E\left[X_{i_{1}j}b_{i_{1}i_{2v+1}}\epsilon_{0i_{2v+1}}X_{i_{2}j}b_{i_{2}i_{2v+2}}\epsilon_{0i_{2v+1}}\times \cdots \times X_{i_{2v}j}b_{i_{2v}i_{4v}}\epsilon_{0i_{4v}}|\tilde{\mathbb{Z}}\right]\\ \;\;\;\;\;\;\;\;\leq \frac{Cq_{0}^{2v}}{n^{4v}}\sum_{i_{1},i_{2},\ldots ,i_{3v}}E\left[X_{i_{1}j}X_{i2j}\times \cdots \times X_{i_{2v}j}\times \epsilon_{0i_{2v+1}}^{2}\epsilon_{0i_{2v+2}}^{2}\times \cdots \times \epsilon_{0i_{3v}}^{2}|\tilde{\mathbb{Z}}\right]\\ \;\;\;\;\;\;\;\;=O\left(q_{0}^{2v}n^{-v}\right). \end{array}$$

Note that for $j\notin P_{0}$, $E\left[X_{ij}|\tilde{\mathbb{Z}}\right]=0$. Then for For $j\notin P_{0}$,

$$\begin{array}{l} E\left[T_{2v,j}|\tilde{\mathbb{Z}}\right]=\frac{1}{n^{2v}}\sum_{i_{1},i_{2},\ldots ,i_{4v}}E\left[X_{i_{1}j}b_{i_{1}i_{2v+1}}\epsilon_{0i_{2v+1}}X_{i_{2}j}b_{i_{2}i_{2v+2}}\epsilon_{0i_{2v+1}}\times \cdots \times X_{i_{2v}j}b_{i_{2v}i_{4v}}\epsilon_{0i_{4v}}|\tilde{\mathbb{Z}}\right]\\ \;\;\;\;\;\;\;\;\leq \frac{Cq_{0}^{2v}}{n^{4v}}\sum_{i_{1},i_{2},\ldots ,i_{2v}}E\left[X_{i_{1j}}^{2}X_{i_{2j}}^{2}\times \cdots \times X_{i_{v}j}^{2}\times \epsilon_{0i_{v+1}}^{2}\epsilon_{0i_{v+2}}^{2}\times \cdots \times \epsilon_{0i_{2v}}^{2}|\tilde{\mathbb{Z}}\right]\\ \;\;\;\;\;\;\;\;=O\left(q_{0}^{2v}n^{-2v}\right). \end{array}$$

In summary, $E\left[T_{2v,j}\right]=O\left(q_{0}^{2v}n^{-v}\right)$ if *j* ∈ *P*_0_ and $E\left[T_{2v,j}\right]=O\left(q_{0}^{2v}n^{-2v}\right)$ if $j\notin P_{0}$. Then we have $\sum_{j=1}^{p}E{\left[{\left(\frac{1}{n}\sum_{i=1}^{n}\left(\mu_{0i}-{\hat{\mu }}_{0i}\right)X_{ij}\right)}^{2v}\right]}^{1/2}=O\left(p_{0}q_{0}^{v}n^{-v/2}+pq_{0}^{v}n^{-v}\right)$. This leads to

$$\begin{array}{l} E\left[\left|T_{\gamma v}\right|\right]\leq O\left(n^{-\left(\gamma -v\right)/2}\right)\times O\left(p_{0}q_{0}^{v}n^{-v/2}+pq_{0}^{v}n^{-v}\right)\\ \;\;\;\;\;=O\left(p_{0}q_{0}^{v}n^{-\gamma /2}+\sqrt{p}n^{-\gamma /2}\sqrt{p}n^{-v/2}q_{0}^{v}\right). \end{array}$$

Note that by assumption C5, $pq_{0}^{4}=o\left(n^{2}\right)$, *v* ≥ 2, and by assumption C4, $p_{0}=O\left(p^{\eta_{1}}\right)$, $q_{0}=O\left(n^{\eta_{2}}\right)$ for some small positive *η*_1_ and *η*_2_, we have $E\left[\left|T_{\gamma v}\right|\right]=o\left(\sqrt{p}n^{-\gamma /2}\right)$, leading to $\left|T_{\gamma v}\right|=o_{p}\left(\sqrt{p}n^{-\gamma /2}\right)$.

In summary, we have proved for any finite *γ*,

$${\left[\left\{L\left(\gamma ,{\hat{\mu }}_{0}\right)-\psi \left(\gamma \right)\right\}/\omega \left(\gamma \right)\right]}_{\gamma \in \Gamma^{\prime }}^{'}={\left[\left\{L\left(\gamma ,\mu_{0}\right)-\psi \left(\gamma \right)\right\}/\omega \left(\gamma \right)\right]}_{\gamma \in \Gamma^{\prime }}^{'}+o_{p}\left(1\right).$$

(ii) Asymptotic null distribution for iSPU(∞). Define

$${\tilde{V}}_{ij}=\left(Y_{i}-{\hat{\mu }}_{0i}\right)X_{ij}/\sqrt{\sigma_{jj}},\;\;\;1\leq i\leq n,\;1\leq j\leq p.$$

Let ${\tilde{W}}_{j}=\sum_{i=1}^{n}{\tilde{V}}_{ij}/\sqrt{n}$. We discuss two cases: *j* ∈ *P*_0_ and $j\notin P_{0}$.

For case *j* ∈ *P*_0_: we define *ϵ* is a small constant. Note that

$$\begin{array}{l} \;\;\;\;\;Pr\left(\underset{j\in P_{0}}{\max}{\tilde{W}}_{j}^{2}>\epsilon \log p\right)\\ \leq Pr\left(\underset{j\in P_{0}}{\max}\left|{\tilde{W}}_{j}\right|>\epsilon {\left(\log p\right)}^{1/2}\right)\\ \leq Pr\left(\underset{j\in P_{0}}{\max}\left|\frac{\sum_{i=1}^{n}\left(Y_{i}-\mu_{0i}+\mu_{0i}-{\hat{\mu }}_{0i}\right)X_{ij}}{\sqrt{\sigma_{jj}n}}\right|>{\left(\epsilon \log p\right)}^{1/2}\right)\\ \leq Pr\left(\underset{j\in P_{0}}{\max}\left|\frac{\sum_{i=1}^{n}\left(Y_{i}-\mu_{0i}\right)X_{ij}}{\sqrt{\sigma_{jj}n}}\right|>\frac{{\left(\epsilon \log p\right)}^{1/2}}{2}\right)\\ \;\;\;\;\;\;+Pr\left(\underset{j\in P_{0}}{\max}\left|\frac{\sum_{i=1}^{n}\left(\mu_{0i}-{\hat{\mu }}_{0i}\right)X_{ij}}{\sqrt{\sigma_{jj}n}}\right|>\frac{{\left(\epsilon \log p\right)}^{1/2}}{2}\right). \end{array}$$

For the first term, we have

$$\leq Pr\left(\underset{j\in P_{0}}{\max}\left|\frac{\sum_{i=1}^{n}\left(Y_{i}-\mu_{0i}\right)X_{ij}}{\sqrt{\sigma_{jj}n}}\right|>\frac{{\left(\epsilon \log p\right)}^{1/2}}{2}\right)\leq p_{0}\leq Pr\left(\left|\frac{\sum_{i=1}^{n}S_{ij}}{\sqrt{\sigma_{jj}n}}\right|>\frac{{\left(\epsilon \log p\right)}^{1/2}}{2}\right).$$

Note that *S*_*ij*_ follows a sub-Gaussian distribution (C2) and $S_{i_{1}j}$ and $S_{i_{2}j}$ are independent for $i_{1}\neq i_{2}$. Suppose $S_{1j},\ldots ,S_{nj}$ be *n* independent random variables such that *S*_*ij*_ follows sub-Gaussian distribution subG(0*, σ*^2^). Then for any $a\in {\mathbb{R}}^{n}$, using a Chernoff bound, we have $Pr\left(\left|\sum_{i=1}^{n}a_{i}S_{ij}\right|>t\right)\leq 2\exp\left(-t^{2}/\left(2\sigma^{2}{\left\|a\right\|}_{2}^{2}\right)\right)$. Similarly, we have

$$\begin{array}{l} \;Pr\left(\underset{j\in P_{0}}{\max}\left|\frac{\sum_{i=1}^{n}\left(Y_{i}-\mu_{0i}\right)X_{ij}}{\sqrt{\sigma_{jj}n}}\right|>\frac{{\left(\epsilon \log p\right)}^{1/2}}{2}\right)\\ \leq p_{0}\times 2\exp\left(-\frac{\epsilon \log p/4}{2}\right)=2p_{0}p^{-\epsilon /8}=o\left(1\right). \end{array}$$

By noting that $p_{0}=p^{\eta_{1}}$, where *η*_1_ is a small constant, we have the last equation.

For the second term, we have

$$\begin{array}{l} \;\;Pr\left(\underset{j\in P_{0}}{\max}\left|\frac{\sum_{i=1}^{n}\left(\mu_{0i}-{\hat{\mu }}_{0i}\right)X_{ij}}{\sqrt{\sigma_{jj}n}}\right|>\frac{{\left(\epsilon \log p\right)}^{1/2}}{2}\right)\\ \leq \mathrm{Pr}\left(\underset{j\in P_{0}}{\max}\left|\frac{\sum_{i_{1},i_{2}}^{n}X_{i_{1}j}\epsilon_{0i_{2}}b_{i_{1}i_{2}}}{\sqrt{\sigma_{jj}n}}\right|>\frac{{\left(\epsilon \log p\right)}^{1/2}}{2}\right)\\ \leq Pr\left(\underset{j\in P_{0}}{\max}\left|\sum_{i_{2}=1}^{n}\epsilon_{0i_{2}}\left(\frac{\sum_{i_{1}}X_{i_{1}j}b_{i_{1}i_{2}}}{\sqrt{\sigma_{jj}n}}\right)\right|>\frac{{\left(\epsilon \log p\right)}^{1/2}}{2}|\underset{i_{2}}{\max}\frac{\sum_{i_{1}}X_{i_{1}j}b_{i_{1}i_{2}}}{\sqrt{\sigma_{jj}n}}<\frac{C}{\sqrt{\sigma_{jj}n}}\right)\\ \;\;\;\;\;\;\;+Pr\left(\underset{i_{2}}{\max}\frac{\sum_{i_{1}}X_{i_{1}j}b_{i_{1}i_{2}}}{\sqrt{\sigma_{jj}n}}\geq \frac{C}{\sqrt{\sigma_{jj}n}}\right). \end{array}$$

We discuss these two terms separately. For the first term, we have

$$\begin{array}{l} \;\;Pr\left(\underset{j\in P_{0}}{\max}\left|\sum_{i_{2}=1}^{n}\epsilon_{0i_{2}}\left(\frac{\sum_{i_{1}}X_{i_{1}j}b_{i_{1}i_{2}}}{\sqrt{\sigma_{jj}n}}\right)\right|>\frac{{\left(\epsilon \log p\right)}^{1/2}}{2}|\underset{i_{2}}{\max}\frac{\sum_{i_{1}}X_{i_{1}j}b_{i_{1}i_{2}}}{\sqrt{\sigma_{jj}n}}<\frac{C}{\sqrt{\sigma_{jj}n}}\right)\\ \leq p_{0}Pr\left(\left|\sum_{i_{2}=1}^{n}\epsilon_{0i_{2}}\left(\frac{\sum_{i_{1}}X_{i_{1}j}b_{i_{1}i_{2}}}{\sqrt{\sigma_{jj}n}}\right)\right|>\frac{{\left(\epsilon \log p\right)}^{1/2}}{2}|\underset{i_{2}}{\max}\frac{\sum_{i_{1}}X_{i_{1}j}b_{i_{1}i_{2}}}{\sqrt{\sigma_{jj}n}}<\frac{C}{\sqrt{\sigma_{jj}n}}\right)\\ \leq p_{0}E\left[Pr\left(\left|\sum_{i_{2}=1}^{n}\epsilon_{0i_{2}}\left(\frac{\sum_{i_{1}}X_{i_{1}j}b_{i_{1}i_{2}}}{\sqrt{\sigma_{jj}n}}\right)\right|>\frac{{\left(\epsilon \log p\right)}^{1/2}}{2}|\underset{i_{2}}{\max}\frac{\sum_{i_{1}}X_{i_{1}j}b_{i_{1}i_{2}}}{\sqrt{\sigma_{jj}n}}<\frac{C}{\sqrt{\sigma_{jj}n}},X,\tilde{\mathbb{Z}}\right)\right]. \end{array}$$

By noting that *ϵ*_0*i*_ follows a sub-Gaussian distribution, we have

$$\begin{array}{l} Pr\left(\underset{j\in P_{0}}{\max}\left|\sum_{i_{2}=1}^{n}\epsilon_{0i_{2}}\left(\frac{\sum_{i_{1}}X_{i_{1}j}b_{i_{1}i_{2}}}{\sqrt{\sigma_{jj}n}}\right)\right|>\frac{{\left(\epsilon \log p\right)}^{1/2}}{2}|\underset{i_{2}}{\max}\frac{\sum_{i_{1}}X_{i_{1}j}b_{i_{1}i_{2}}}{\sqrt{\sigma_{jj}n}}<\frac{C}{\sqrt{\sigma_{jj}n}},X,\tilde{\mathbb{Z}}\right)\\ \leq p_{0}\times 2\exp\left(-\frac{\epsilon \log p/4}{2C^{2}}\right)=2p_{0}p^{-\epsilon /\left(8C^{2}\right)}=o\left(1\right). \end{array}$$

For the second term, we have

$$Pr\left(\underset{i_{2}}{\max}\frac{\sum_{i_{1}}X_{i_{1}j}b_{i_{1}i_{2}}}{\sqrt{\sigma_{jj}n}}\geq \frac{C}{\sqrt{\sigma_{jj}n}}\right)\leq nE\left[Pr\left(\frac{\sum_{i_{1}}X_{i_{1}j}}{n}\geq C/q_{0}|\tilde{\mathbb{Z}}\right)\right].$$

By central limit theorem and the Gaussian tail inequality, we have

$$Pr\left(\underset{i_{2}}{\max}\frac{\sum_{i_{1}}X_{i_{1}j}b_{i_{1}i_{2}}}{\sqrt{\sigma_{jj}n}}\geq \frac{C}{\sqrt{\sigma_{jj}n}}\right)\leq n\times \frac{2\exp\left(-{\left(Cn^{1/2}/q_{0}\right)}^{2}/2\right)}{Cn^{1/2}/q_{0}}.$$

By noting that $q_{0}=n^{\eta_{2}}$ for a small positive *η*_2_, we have

$$Pr\left(\underset{i_{2}}{\max}\frac{\sum_{i_{1}}X_{i_{1}j}b_{i_{1}i_{2}}}{\sqrt{\sigma_{jj}n}}\geq \frac{C}{\sqrt{\sigma_{jj}n}}\right)\leq Cn^{1/2+\eta_{2}}\times \exp\left(-n^{1-2\eta_{2}}/2\right)=o\left(1\right).$$

In summary, as *n, p* → ∞, $Pr\left(\max_{j\in P_{0}}{\tilde{W}}_{j}^{2}>\epsilon \log p\right)=o\left(1\right)$. Then we focus on the second situation. Define $V_{ij}=\left(Y_{i}-\mu_{0i}\right)X_{ij}/\sqrt{\sigma_{jj}}$, ${\hat{V}}_{ij}=V_{ij}I\left(\left|V_{ij}\right|\leq \tau_{n}\right)$ for i=1,…,n and j=1,…,p, where $\tau_{n}=2\eta^{-0.5}\sqrt{\log\left(p+n\right)}$. Further define $W_{j}=\sum_{i=1}^{n}\left(Y_{i}-\mu_{0i}\right)X_{ij}/\sqrt{\sigma_{jj}n}$ and ${\hat{W}}_{j}=\sum_{i=1}^{n}{\hat{V}}_{ij}/\sqrt{n}$. Then we have

$$\begin{array}{l} Pr\left(\underset{j\notin P_{0}}{\max}\left|{\tilde{W}}_{j}-W_{j}\right|\geq \frac{1}{\log p}\right)\\ \leq np\underset{j\notin P_{0}}{\max}Pr\left(\left|V_{1j}\right|\geq \tau_{n}\right)+Pr\left(\underset{j\notin P_{0}}{\max}\left|\sum_{i=1}^{n}\frac{\left(\mu_{0i}-{\hat{\mu }}_{0i}\right)X_{ij}}{\sqrt{\sigma_{jj}n}}\right|\geq \frac{1}{\log p}\right). \end{array}$$

From the proof of Lemma 1, the first term is *O*(1*/p* + 1*/n*) and thus we only need discuss the second term. By the Markov inequality and the Jensen’s inequality,

$$\begin{array}{l} Pr\left(\underset{j\notin P_{0}}{\max}\left|\sum_{i=1}^{n}\frac{\left(\mu_{0i}-{\hat{\mu }}_{0i}\right)X_{ij}}{\sqrt{\sigma_{jj}n}}\right|\geq \frac{1}{\log p}\right)\\ \;\leq Pr\left(\underset{j\notin P_{0}}{\max}{\left(\sum_{i=1}^{n}\frac{\left(\mu_{0i}-{\hat{\mu }}_{0i}\right)X_{ij}}{\sqrt{\sigma_{jj}n}}\right)}^{32}\geq \frac{1}{{\left(\log p\right)}^{32}}\right)\\ \;\leq pPr\left({\left(\sum_{i=1}^{n}\frac{\left(\mu_{0i}-{\hat{\mu }}_{0i}\right)X_{ij}}{\sqrt{\sigma_{jj}n}}\right)}^{32}\geq \frac{1}{{\left(\log p\right)}^{32}}\right)\\ \;\leq p\log pE\left[{\left(\sum_{i=1}^{n}\frac{\left(\mu_{0i}-{\hat{\mu }}_{0i}\right)X_{ij}}{\sqrt{\sigma_{jj}n}}\right)}^{32}\right]\\ \;\leq p\log p\times O\left(n^{-16}q_{0}^{16}\right)=o\left(1\right). \end{array}$$

Thus, we have $Pr\left(\max_{1\leq j\leq p}\left|{\tilde{W}}_{j}-W_{j}\right|\geq 1/\log p\right)=o\left(1\right)$ as *n, p* → ∞. Further note that

$$\left|\underset{j\notin P_{0}}{\max}W_{j}^{2}-\underset{j\notin P_{0}}{\max}{\tilde{W}}_{j}^{2}\right|\leq 2\underset{j\notin P_{0}}{\max}\left|W_{j}\right|\underset{j\notin P_{0}}{\max}\left|W_{j}-{\tilde{W}}_{j}\right|+\underset{j\notin P_{0}}{\max}{\left|W_{j}-{\tilde{W}}_{j}\right|}^{2}.$$

The above two inequalities indicate that when *n, p* → ∞, $\left|\max_{j\notin P_{0}}W_{j}^{2}-\max_{j\notin P_{0}}{\tilde{W}}_{j}^{2}\right|\to 0$ By Cai et al. (2014), we have

$$Pr\left(\underset{j\notin P_{0}}{\max}{\tilde{W}}_{j}^{2}-2\log p+\log\log p\leq x\right)\to \exp\left\{-\pi^{-1/2}\exp\left(-x/2\right)\right\}.$$

Note that

$$\underset{1\leq j\leq p}{\max}{\tilde{W}}_{j}^{2}=\max\left(\underset{j\in P_{0}}{\max}{\tilde{W}}_{j}^{2},\underset{j\notin P_{0}}{\max}{\tilde{W}}_{j}^{2}\right)=\underset{j\notin P_{0}}{\max}{\tilde{W}}_{j}^{2}.$$

Thus,

$$Pr\left(\underset{1\leq j\leq p}{\max}{\tilde{W}}_{j}^{2}-2\log p+\log\log p\leq x\right)\to \exp\left\{-\pi^{-1/2}\exp\left(-x/2\right)\right\}.$$

Note that ${\hat{\sigma }}_{jj}=\left(1+o\left(1\right)\right)\sigma_{jj}$ and by Slutsky’s theorem, we have

$$Pr\left(\underset{1\leq j\leq p}{\max}\frac{n{\left(\frac{1}{n}\sum_{i=1}^{n}U_{ij}\right)}^{2}}{\overset{\vee }{\sigma_{jj}}}-2\log p+\log\log p\leq x\right)\to \exp\left\{-\pi^{-1/2}\exp\left(-x/2\right)\right\}.$$

(iii) By proof in (i) and (ii), we have

$${\left[\left\{L\left(\gamma ,{\hat{\mu }}_{0}\right)-\psi \left(\gamma \right)\right\}/\omega \left(\gamma \right)\right]}_{\gamma \in \Gamma^{\prime }}^{'}={\left[\left\{L\left(\gamma ,\mu_{0}\right)-\psi \left(\gamma \right)\right\}/\omega \left(\gamma \right)\right]}_{\gamma \notin \Gamma^{\prime }}^{'}+o_{p}\left(1\right)$$

and $L\left(\infty ,{\hat{\mu }}_{0}\right)=L\left(\infty ,\mu_{0}\right)+o_{p}\left(1\right)$. By Lemma 1, ${\left[\left\{L\left(\gamma ,\mu_{0}\right)-\psi \left(\gamma \right)\right\}/\omega \left(\gamma \right)\right]}_{\gamma \in \Gamma^{\prime }}^{'}$ is asymptotically independent with *L*(∞, *µ*_0_). Note that *o*_*p*_(1) is asymptotic independent with *L*(∞, *µ*_0_) and ${\left[\left\{L\left(\gamma ,\mu_{0}\right)-\psi \left(\gamma \right)\right\}/\omega \left(\gamma \right)\right]}_{\gamma \in \Gamma^{\prime }}^{'}$, thus ${\left[\left\{L\left(\gamma ,{\hat{\mu }}_{0}\right)-\psi \left(\gamma \right)\right\}/\omega \left(\gamma \right)\right]}_{\gamma \in \Gamma^{\prime }}^{'}$ is asymptotically independent with $L\left(\infty ,{\hat{\mu }}_{0}\right)$. This completes the proof.

## Appendix D. Details of asymptotic power analysis

To derive some propositions, we define some additional notation. Under an alternative $H_{A}:\beta \neq 0$, we denote the mean and variance of *L*(*γ, µ*_0_) with *γ <* ∞ by $\psi_{A}\left(\gamma \right)=\sum_{j=1}^{p}\psi_{A}^{\left(j\right)}\left(\gamma \right)$ with $\psi_{A}^{\left(j\right)}\left(\gamma \right)=E\left[L^{\left(j\right)}\left(\gamma ,\mu_{0}\right)|H_{A}\right]$ for 1 ≤ *j* ≤ *p*, and by $\omega_{A}^{2}=\mathrm{var}\left[L\left(\gamma ,\mu_{0}\right)|H_{A}\right]$, respectively. Define ${\tilde{S}}_{ij}\equiv \left(Y_{i}-\mu_{0i}^{A}\right)X_{ij}$ for 1 ≤ *i* ≤ *n* and 1 ≤ *j* ≤ *p* and ${\tilde{\sigma }}_{kj}=\mathrm{cov}\left[{\tilde{S}}_{1k},{\tilde{S}}_{1j}\right]$ for 1 ≤ *k, j* ≤ *p*.

Next, we introduce Propositions 1 and 2 to calculate the *ψ*_*A*_(*γ*) and $\omega_{A}^{2}\left(\gamma \right)$, respectively.

Proposition 1. Under assumptions C8–C9 and $H_{A}:\beta \neq 0$, we have

$$\psi_{A}^{\left(j\right)}\left(\gamma \right)\sim {\tilde{\psi }}^{\left(j\right)}\left(\gamma \right)+\sum_{c=1}^{\gamma }\left(\begin{array}{l} \gamma \\ c \end{array}\right)\Delta_{j}^{c}{\tilde{\psi }}^{\left(j\right)}\left(\gamma -c\right),$$

where ∼ stands for the two sides are in the same order, ${\tilde{\psi }}^{\left(j\right)}\left(1\right)=0$, ${\tilde{\psi }}^{\left(j\right)}\left(\gamma \right)=\frac{\gamma !}{d!2^{d}}n^{-d}{\tilde{\sigma }}_{jj}^{d}+o\left(n^{-d}\right)$ if γ = 2d, and ${\tilde{\psi }}^{\left(j\right)}\left(\gamma \right)=o\left(n^{-\left(d+1\right)}\right)$ if γ = 2d + 1. In particular $\mu_{A}^{\left(j\right)}\left(1\right)=\Delta_{j}$ and $\mu_{A}\left(1\right)=\sum_{j=1}^{p}\Delta_{j}$.

**Proof of Proposition 1.** Under the *H*_*A*_, the true conditional mean of *Y*_*i*_ is $\mu_{0i}^{A}$. Then similarly to Theorem 1, we can calculate ${\tilde{\psi }}^{\left(j\right)}\left(\gamma \right)$ accordingly.

Under the alternative *H*_*A*_, it is trivial to find $\mu_{A}\left(1\right)=\sum_{i=1}^{p}\Delta_{i}$ since $\tilde{\psi }\left(1\right)=0$. Next, we focus on *γ* ≥ 2. The mean function of *L*^(*j*)^(*γ*) under *H*_*A*_ equals

$$\begin{array}{l} E\left[L^{\left(j\right)}\left(\gamma \right)\right]=E\left[{\left(\frac{1}{n}\sum_{i=1}^{n}U_{ij}\right)}^{\gamma }\right]\\ \;\;\;\;\;\;\;=E\left[{\left(\frac{1}{n}\sum_{i=1}^{n}\left(Y_{i}-\mu_{0i}^{A}\right)X_{ij}+\frac{1}{n}\sum_{i=1}^{n}\left(\mu_{0i}^{A}-\mu_{0i}\right)X_{ij}\right)}^{\gamma }\right]\\ \;\;\;\;\;\;\;=\sum_{0\leq a\leq \gamma }\left(\begin{array}{l} \gamma \\ a \end{array}\right)E\left[{\left(\frac{1}{n}\sum_{i=1}^{n}\left(Y_{i}-\mu_{0i}^{A}\right)X_{ij}\right)}^{a}\right]E\left[{\left(\frac{1}{n}\sum_{i=1}^{n}\left(\mu_{0i}^{A}-\mu_{0i}\right)X_{ij}\right)}^{\gamma -a}\right]. \end{array}$$

Note that under the local alternative considered here, $\Delta_{j}=O\left(n^{-1/2}{\left(\log p\right)}^{\kappa }\right)$ with *κ >* 0 for 1 ≤ *j* ≤ *p*. Then $E\left[\left|\left(\mu_{0i}^{A}-\mu_{0i}\right)X_{1j}\right|\right]\leq C\left|\Delta_{j}\right|$ for any positive integer *γ*, where *C* is some constant. Thus $E\left[{\left(\frac{1}{n}\sum_{i=1}^{n}\left(\mu_{0i}^{A}-\mu_{0i}\right)X_{ij}\right)}^{a}\right]=\Delta_{j}^{a}\left(1+o\left(1/n\right)\right)$. Then we have

$$\psi_{A}^{\left(j\right)}\left(\gamma \right)={\tilde{\psi }}^{\left(j\right)}+\sum_{c=1}^{\gamma }\left(\begin{array}{l} \gamma \\ c \end{array}\right)\Delta_{j}^{c}{\tilde{\psi }}^{\left(j\right)}\left(\gamma -c\right)\left[1+o\left(1/n\right)\right].$$

Then Proposition 1 follows directly from the above equation.

Proposition 2. Under assumptions C8–C9 and $H_{A}:\beta \neq 0$, we have

$$\omega_{A}^{2}\left(\gamma \right)\sim \psi_{A}\left(2\gamma \right)-\sum_{j=1}^{p}\psi_{A}^{\left(j\right)}{\left(\gamma \right)}^{2}+\sum_{k\neq j}\sum_{h=0}^{\gamma }\sum_{l=0}^{\gamma }\left(\begin{array}{l} \gamma \\ h \end{array}\right)\left(\begin{array}{l} \gamma \\ l \end{array}\right)\Delta_{k}^{h}\Delta_{j}^{l}r_{kj}\left(\gamma -h,\gamma -l\right),$$

where

$$r_{kj}\left(h,l\right)=\{\begin{array}{ll} \frac{1}{n^{c}}\sum_{\begin{array}{c} 2c_{1}+c_{3}=h\\ 2c_{2}+c_{3}=l\\ c_{3}>0 \end{array}}\frac{h!l!}{c_{3}!c_{1}!c_{2}!2^{c_{1}+c_{2}}}{\tilde{\sigma }}_{kk}^{c_{1}}{\tilde{\sigma }}_{jj}^{c_{3}}{\tilde{\sigma }}_{kj}^{c_{3}}+o\left(n^{-c}\right) & if\;h+l=2c;\\ \frac{1}{n^{c+1}}\sum_{\begin{array}{c} a+b=3\\ 2c_{1}+c_{3}=h-a\\ 2c_{2}+c_{3}=l-b \end{array}}\frac{h!l!}{a!b!c_{3}!c_{1}!c_{2}!2^{c_{1}+c_{2}}}{\tilde{\sigma }}_{kk}^{c_{1}}{\tilde{\sigma }}_{jj}^{c_{3}}{\tilde{\sigma }}_{kj}^{c_{3}}m_{k^{a}j^{b}}+o\left(n^{-\left(c+1\right)}\right) & if\;h+l=2c+1, \end{array}$$

with $m_{k^{a}j^{b}}=E\left[{\left(\left(Y_{1}-\mu_{01}^{A}\right)X_{1k}\right)}^{a}{\left(\left(Y_{1}-\mu_{01}^{A}\right)X_{1j}\right)}^{b}\right]$.

**Proof of Proposition 2.** Under the local alternative,

$$\begin{array}{l} \omega_{A}^{2}\left(\gamma \right)=E\left[{\left\{\sum_{j=1}^{p}{\left(\frac{1}{n}\sum_{i=1}^{n}\left(Y_{i}-\mu_{0i}\right)X_{ij}\right)}^{\gamma }\right\}}^{2}\right]-E{\left[\sum_{j=1}^{p}{\left(\frac{1}{n}\sum_{i=1}^{n}\left(Y_{i}-\mu_{0i}\right)X_{ij}\right)}^{\gamma }\right]}^{2}\\ \;\;\;\;\;\;=\psi_{A}\left(2\gamma \right)-\sum_{i=1}^{p}{\left\{\psi_{A}^{\left(i\right)}\left(\gamma \right)\right\}}^{2}-\sum_{k\neq j}\psi_{A}^{\left(k\right)}\left(\gamma \right)\psi_{A}^{\left(j\right)}\left(\gamma \right)\\ \;\;\;\;\;\;\;\;\;\;\;+E\left[\sum_{k\neq j}{\left(\frac{1}{n}\sum_{i=1}^{n}\left(Y_{i}-\mu_{0i}\right)X_{ik}\right)}^{\gamma }{\left(\frac{1}{n}\sum_{i=1}^{n}\left(Y_{s}-\mu_{0i}\right)X_{ij}\right)}^{\gamma }\right]\\ \;\;\;\;\;=\psi_{A}\left(2\gamma \right)-\sum_{i=1}^{p}{\left\{\psi_{A}^{\left(i\right)}\left(\gamma \right)\right\}}^{2}\\ \;\;\;\;\;\;\;\;\;\;\;-\sum_{k\neq j}\left[\sum_{c=0}^{\gamma }\left(\begin{array}{l} \gamma \\ c \end{array}\right)\Delta_{k}^{c}{\tilde{\psi }}^{\left(k\right)}\left(\gamma -c\right)\left(1+o\left(1/n\right)\right)\right]\left[\sum_{c=0}^{\gamma }\left(\begin{array}{l} \gamma \\ c \end{array}\right)\Delta_{j}^{c}{\tilde{\psi }}^{\left(j\right)}\left(\gamma -c\right)\left(1+o\left(1/n\right)\right)\right]\\ \;\;\;\;\;\;\;\;\;\;\;+\sum_{k\neq j}\sum_{h=0}^{\gamma }\sum_{l=0}^{\gamma }\left(\begin{array}{l} \gamma \\ h \end{array}\right)\left(\begin{array}{l} \gamma \\ l \end{array}\right)\Delta_{k}^{h}\Delta_{j}^{l}E\left[{\left(\frac{1}{n}\sum_{i=1}^{n}{\tilde{S}}_{ik}\right)}^{\gamma -h}{\left(\frac{1}{n}\sum_{i=1}^{n}{\tilde{S}}_{ij}\right)}^{\gamma -l}\right]\left(1+o\left(1/n\right)\right). \end{array}$$

By the derivation of Proposition 3 in Wu et al. (2019), the last two terms in the above equation can be simplified as

$$\begin{array}{l} \sum_{k\neq j}\sum_{h=0}^{\gamma }\sum_{l=0}^{\gamma }\left(\begin{array}{l} \gamma \\ h \end{array}\right)\left(\begin{array}{l} \gamma \\ l \end{array}\right)\Delta_{k}^{h}\Delta_{j}^{l}\left(E\left[{\left(\frac{1}{n}\sum_{i=1}^{n}{\tilde{S}}_{ik}\right)}^{\gamma -h}{\left(\frac{1}{n}\sum_{i=1}^{n}{\tilde{S}}_{ij}\right)}^{\gamma -l}\right]-\tilde{\psi }\left(k\right)\left(\gamma -h\right){\tilde{\psi }}^{\left(j\right)}\left(\gamma -l\right)\right)\\ \sim \sum_{k\neq j}\sum_{h=0}^{\gamma }\sum_{l=0}^{\gamma }\left(\begin{array}{l} \gamma \\ h \end{array}\right)\left(\begin{array}{l} \gamma \\ l \end{array}\right)\Delta_{k}^{h}\Delta_{j}^{l}r_{kj}\left(\gamma -h,\gamma -l\right). \end{array}$$

This completes the proof.

**Proof of iSPU(1) is more powerful when** ∆_*j*_ **is fixed at the same level.** We further assume ∆_*j*_ equals to ∆.for $j\in S_{\eta }$ under the alternative. Note that the asymptotic power of iSPU(*γ*) goes to 1 if $\left(\mu_{A}\left(\gamma \right)-\mu_{0}\left(\gamma \right)\right)n^{\gamma /2}p^{-1/2}\to \infty$, which implies that for any finite *γ*, a sufficient condition for the asymptotic power of iSPU(*γ*) goes to 1 is

$$\frac{\Delta }{n^{-1/2}p^{\left(2\eta -1\right)/2\gamma }}\to \infty ,\;\;\text{as}\;p,n\to \infty .$$

This sufficient condition comes from the fact that $\Delta =O\left(n^{-1/2}{\left(\log p\right)}^{\kappa }\right)$ and $\mu_{A}\left(\gamma \right)-\mu_{0}\left(\gamma \right)\sim \sum_{i=1}^{p}\sum_{c=1}^{\gamma }\Delta^{c}O\left(n^{-\left(\gamma -c\right)/2}\right)\sim p^{1-\eta }\Delta^{\gamma }$. Since 0 *< η <* 1/2, $p^{\left(2\eta -1\right)/\left(2\gamma \right)}\to 0$ as *p* → ∞. Thus, to compare the asymptotic powers of different iSPU(*γ*), we focus on the local alternative such that $n^{1/2}\Delta \to 0$, as *p, n* → ∞. Equivalently, we write $\Delta =n^{-1/2}r^{1/2}$, where *r* → 0 as *p, n* → ∞.

Then we calculate $\psi_{A}\left(\gamma \right)-\tilde{\psi }\left(\gamma \right)$. When *γ* is odd, by Proposition 1,

$$\begin{array}{l} \psi_{A}\left(\gamma \right)-\tilde{\psi }\left(\gamma \right)=\sum_{j=1}^{p}\sum_{c=1}^{\gamma }\left(\begin{array}{l} \gamma \\ c \end{array}\right)\Delta_{j}^{c}{\tilde{\psi }}^{\left(j\right)}\left(\gamma -c\right)\left(1+o\left(1/n\right)\right)\\ \;\;\;\;\;\;\;\;\sim \gamma \sum_{j=1}^{p}\Delta_{j}{\tilde{\psi }}^{\left(j\right)}\left(\gamma -1\right)\\ \;\;\;\;\;\;\;\;\sim \gamma \sum_{j=1}^{p}\Delta_{j}\frac{\left(\gamma -1\right)!}{\left(\left(\gamma -1\right)/2\right)!2^{\left(\gamma -1\right)/2}}n^{-\left(\gamma -1\right)/2}\\ \;\;\;\;\;\;\;\;\sim p^{1-\eta }\Delta \frac{\gamma !}{\left(\left(\gamma -1\right)/2\right)!2^{\left(\gamma -1\right)/2}}n^{-\gamma /2}n^{1/2}\\ \;\;\;\;\;\;\;\;\sim \frac{\gamma !}{\left(\left(\gamma -1\right)/2\right)!2^{\left(\gamma -1\right)/2}}\times r^{1/2}p^{1-\eta }n^{-\gamma /2}. \end{array}$$

Similarly, when *γ* is even

$$\begin{array}{l} \psi_{A}\left(\gamma \right)-\tilde{\psi }\left(\gamma \right)\sim \gamma \sum_{j=1}^{p}\Delta_{j}{\tilde{\psi }}^{\left(j\right)}\left(\gamma -1\right)+\left(\begin{array}{l} \gamma \\ 2 \end{array}\right)\sum_{j=1}^{p}\Delta_{j}^{2}{\tilde{\psi }}^{\left(j\right)}\left(\gamma -2\right)\\ \;\;\;\;\;\;\;\;\sim p^{1-\eta }n^{-1/2}r^{1/2}o\left(n^{-\gamma /2}\right)+p^{1-\eta }nrO\left(n^{-\left(\gamma +2\right)/2}\right)\\ \;\;\;\;\;\;\;\;\sim o\left(r^{1/2}p^{1-\eta }n^{-\gamma /2}\right). \end{array}$$

Further, under this local alternative, $\omega_{A}\left(\gamma \right)\sim \tilde{\omega }\left(\gamma \right)\sim c_{\gamma }p^{1/2}n^{-\gamma /2}$, where *c*_*γ*_ is some constant and can be calculated by Proposition 2. Next, we study $\left\{\psi_{A}\left(\gamma \right)-\tilde{\psi }\left(\gamma \right)\right\}/\omega_{A}\left(\gamma \right)$, which determines the power. As *n, p* → ∞,

$$\frac{\psi_{A}\left(\gamma \right)-\tilde{\psi }\left(\gamma \right)}{\omega_{A}\left(\gamma \right)}\sim \frac{\gamma !}{\left(\left(\gamma -1\right)/2\right)!2^{\left(\gamma -1\right)/2}c_{\gamma }}\times r^{1/2}p^{1/2-\eta },\;\;\;\;\gamma \;\text{is}\;\text{odd},$$

$$\frac{\psi_{A}\left(\gamma \right)-\tilde{\psi }\left(\gamma \right)}{\omega_{A}\left(\gamma \right)}\sim o\left(1\right)\times r^{1/2}p^{1/2-\eta },\;\;\;\;\;\;\;\;\;\;\gamma \;\text{is}\;\text{odd}.$$

The above results show that the asymptotic power of iSPU(*γ*) does not converge to 1 if $r^{1/2}p^{1/2-\eta }<\infty$. Thus we focus on the local alternative when *r* → 0 and $r^{1/2}p^{1/2-\eta }\to \infty$. Then the asymptotic power of iSPU with odd *γ* goes to 1 while iSPU with even *γ* does not, that is, under the considered alternative, iSPU with odd *γ* is more powerful than iSPU with even *γ*. Therefore, we only need to focus on odd *γ*’s and compare their power. To find which odd *γ* yields an asymptotically more powerful test, we only need to find which *γ* maximizes $\gamma !/\left(\left(\left(\gamma -1\right)/2\right)!2^{\left(\gamma -1\right)/2}c_{\gamma }\right)$. To simplify our discussion, we first consider the situation where ${\tilde{\sigma }}_{ij}=0$ for i≠j. In this case

$$\frac{\gamma !}{\left(\left(\gamma -1\right)/2\right)2^{\left(\gamma -1\right)/2}c_{\gamma }}=\frac{\gamma !}{\left(\left(\gamma -1\right)/2\right)!2^{\left(\gamma -1\right)/2}\sqrt{\left(2\gamma \right)!/\left(\gamma !2^{\gamma }\right)-{\left(\gamma !\right)}^{2}/\left({\left[\left(\gamma /2\right)!\right]}^{2}2^{\gamma }\right)}},$$

which has maximum value 1.66 at *γ* = 1. More generally, under the situation where ${\tilde{\sigma }}_{ij}\geq 0$, a similar calculation gives that iSPU(1) is asymptotically more powerful than iSPU test with other *γ*. This completes the proof.

**Proof of iSPU(2) is more powerful when the absolute values of** ∆_*j*_ **are the same but half being positive while the other half being negative.** We assume |∆_*j*_| equals to ∆ for $j\in S_{\eta }$ under the alternative. Like previous subsection, we consider the local alternative with $\Delta =n^{-1/2}r^{1/2}$, where *r* → 0 as *p, n* → ∞. Similarly, we calculate $\psi_{A}\left(\gamma \right)-\hat{\psi }\left(\gamma \right)$ for both odd and even *γ*.

For *γ* = 1, we have

$$\psi_{A}\left(1\right)-\tilde{\psi }\left(1\right)\sim \sum_{j=1}^{p}\Delta_{j}\sim 0.$$

When *γ* = 3, by noting that ${\tilde{\psi }}^{\left(j\right)}\left(1\right)=0$ for 1 ≤ *j* ≤ *p*, we have

$$\begin{array}{l} \psi_{A}\left(3\right)-\tilde{\psi }\left(3\right)=\sum_{j=1}^{p}\sum_{c=1}^{3}\left(\begin{array}{c} 3\\ c \end{array}\right)\Delta_{j}^{c}{\tilde{\psi }}^{\left(j\right)}\left(3-c\right)\left(1+o\left(1/n\right)\right)\\ \;\;\;\;\;\;\;\sim \sum_{j=1}^{p}\left(\Delta_{j}^{3}+\Delta_{j}{\tilde{\psi }}^{\left(j\right)}\left(2\right)\right)\\ \;\;\;\;\;\;\;\sim 0. \end{array}$$

For odd *γ >* 3, we have

$$\begin{array}{l} \psi_{A}\left(\gamma \right)-\tilde{\psi }\left(\gamma \right)=\sum_{j=1}^{p}\sum_{c=1}^{\gamma }\left(\begin{array}{l} \gamma \\ c \end{array}\right)\Delta_{j}^{c}{\tilde{\psi }}^{\left(j\right)}\left(\gamma -c\right)\left(1+o\left(1/n\right)\right)\\ \;\;\;\;\;\;\;\;\;\sim \left(\begin{array}{l} \gamma \\ 2 \end{array}\right)\sum_{j=1}^{p}\Delta_{j}^{2}{\tilde{\psi }}^{\left(j\right)}\left(\gamma -2\right)\\ \;\;\;\;\;\;\;\sim p^{1-\eta }nr\times o\left(n^{-\left(\gamma -1\right)/2}\right)\\ \;\;\;\;\;\;\;\sim o\left(rp^{1-\eta }n^{-\left(\gamma +1\right)/2}\right). \end{array}$$

Similarly, for even *γ* ≥ 2, we have

$$\begin{array}{l} \psi_{A}\left(\gamma \right)-\tilde{\psi }\left(\gamma \right)=\sum_{j=1}^{p}\sum_{c=1}^{\gamma }\left(\begin{array}{l} \gamma \\ c \end{array}\right)\Delta_{j}^{c}{\tilde{\psi }}^{\left(j\right)}\left(\gamma -c\right)\left(1+o\left(1/n\right)\right)\\ \;\;\;\;\;\;\;\;\sim \left(\begin{array}{l} \gamma \\ 2 \end{array}\right)\sum_{j=1}^{p}\Delta_{j}^{2}{\tilde{\psi }}^{\left(j\right)}\left(\gamma -2\right)\\ \;\;\;\;\;\;\;\;\sim \frac{\gamma \left(\gamma -1\right)}{2}p^{1-\eta }\Delta^{2}\frac{\left(\gamma -2\right)!}{\left(\left(\gamma -2\right)/2\right)!2^{\left(\gamma -2\right)/2}}n^{-\left(\gamma -2\right)/2}\\ \;\;\;\;\;\;\;\;\sim \frac{\gamma !}{\left(\left(\gamma -2\right)/2\right)!2^{\gamma /2}}p^{1-\eta }rn^{-\gamma /2}. \end{array}$$

Again, under this local alternative, $\omega_{A}\left(\gamma \right)\sim \tilde{\omega }\left(\gamma \right)\sim c_{\gamma }p^{1/2}n^{-\gamma /2}$, where *c*_*γ*_ is some constant. Next, we study $\left\{\mu_{A}\left(\gamma \right)-\mu_{0}\left(\gamma \right)\right\}/\sigma_{A}\left(\gamma \right)$, which determines the power. As *n, p* → ∞,

$$\frac{\psi_{A}\left(\gamma \right)-\tilde{\psi }\left(\gamma \right)}{\omega_{A}\left(\gamma \right)}\sim o\left(rp^{1/2-\eta }n^{-1/2}\right),\;\;\;\;\;\;\;\gamma \;\text{is}\;\text{odd},$$

$$\frac{\psi_{A}\left(\gamma \right)-\tilde{\psi }\left(\gamma \right)}{\omega_{A}\left(\gamma \right)}\sim \frac{\gamma !}{c_{\gamma }\left(\left(\gamma -2\right)/2\right)!2^{\gamma /2}}rp^{1/2-\eta },\;\;\;\;\gamma \;\text{is}\;\text{even}.$$

These results show that if $rp^{1/2-\eta }<\infty$, the asymptotic power of iSPU(*γ*) does not converge to 1. Thus we discuss the local alternative when *r* → 0 and $rp^{1/2-\eta }<\infty$. Then the asymptotic power of iSPU with even *γ* goes to 1 while iSPU with odd *γ* does not. In other words, under the considered alternative here, iSPU with even *γ* is more powerful than iSPU with odd *γ*. Therefore, we only need to focus on iSPU with even *γ*s and compare their power. To find which even *γ* yields an asymptotically more powerful test, we need to find which *γ* maximizes $\gamma !/\left(c_{\gamma }\left(\left(\gamma -2\right)/2\right)!2^{\gamma /2}\right)$. We first consider the situation where ${\tilde{\sigma }}_{ij}=0$ for i≠j. In this case

$$\frac{\gamma !}{c_{\gamma }\left(\left(\gamma -2\right)/2\right)2^{\gamma /2}}=\frac{\gamma !}{\left(\left(\gamma -2\right)/2\right)!2^{\gamma /2}\sqrt{\left(2\gamma \right)!/\left(\gamma !2^{\gamma }\right)-{\left(\gamma !\right)}^{2}/\left({\left[\left(\gamma /2\right)!\right]}^{2}2^{\gamma }\right)}},$$

which has maximum value $\sqrt{2}$ at *γ* = 2. More generally, under the situation where ${\tilde{\sigma }}_{ij}\geq 0$, a similar calculation yields that iSPU(2) is asymptotically more powerful than iSPU test with other *γ*. This completes the proof.
