# Supplementary material for: A Regularization-Based Adaptive Test for High-Dimensional Generalized Linear Models
Source: J Mach Learn Res. Author manuscript; Available in PMC 2020 Aug 13. (PMC7425805)
Supplement: 1 [file NIHMS1605534-supplement-1.pdf]

Appendix E. Supplementary Tables and Figures

Table S1: Empirical Type I error rates of various tests under $G \times E$ interaction simulations with $n = 2000$, $p = 200$ and various $q_1 = q_2$. n, p, q_1, and q_2 stand for the sample size, number of coefficients in $G \times E$ interaction effects, number of positive coefficients in main genetics effects, and number of negative coefficients in main genetics effects, respectively. * Conservative Type I error rates.

$q_1 = q_2$	2	5	7	10	20	30
GESAT	0.098	0.105	0.108	0.094	0.095	0.095
aiSPU(Oracle)	0.060	0.053	0.067	0.056	0.052	0.055
aiSPU(Lasso)	0.054	0.045	0.056	0.046	0.031	0.030*
aiSPU(Ridge)	0.052	0.044	0.058	0.044	0.037	0.029*
aiSPU(TLP)	0.058	0.056	0.067	0.059	0.056	0.064
aiSPU(Full)	0.085	0.104	0.107	0.097	0.084	0.093

Table S2: Empirical Type I error rates of various tests in rare variants simulations with $n = 2000$, $q_1 = 2$, $q_2 = 0$, and various p. n, p, q_1, and q_2 stand for the sample size, number of terms in $G \times E$ interaction, number of the positive genetic main effects, and number of the negative genetic main effects, respectively. * Inflated Type I error rates.

p	25	50	70	100	200	300	400	500
iSKAT	0.050	0.077	0.079	0.114*	0.229*	0.560*	0.909*	0.999*
MiSTi	0.051	0.060	0.085	0.088	0.201*	0.514*	0.881*	0.995*
Full	0.043	0.054	0.080	0.089	0.197*	0.557*	0.953*	1.000*
aiSPU(Oracle)	0.037	0.041	0.066	0.066	0.048	0.060	0.060	0.049
aiSPU(TLP)	0.043	0.039	0.060	0.064	0.043	0.053	0.058	0.049

Table S3: Empirical Type I error rates (in percentage) of various tests in rare variants simulations with $n = 2000$, $p = 300$ and various $q_1 = q_2$. n, p, q_1, and q_2 stand for the sample size, number of terms in $G \times E$ interaction, number of the positive genetic main effects, and number of the negative genetic main effects, respectively. * Inflated Type I error rates.

$q_1 = q_2$	2	5	7	10	20	30	50
iSKAT	0.535*	0.534*	0.53*	0.545*	0.550*	0.573*	0.573*
MiSTi	0.508*	0.504*	0.502*	0.508*	0.509*	0.503*	0.536*
Full	0.538*	0.539*	0.535*	0.555*	0.550*	0.573*	0.602*
aiSPU(Oracle)	0.046	0.041	0.052	0.051	0.061	0.071	0.070
aiSPU(TLP)	0.047	0.042	0.054	0.047	0.058	0.071	0.061

Table S4: Empirical Type I errors and power (in percentage) of various tests under $G \times E$ interactions with $p = 1000$ and $n = 200$. Zero signal strength $c = 0$ represents Type I errors, while $c \neq 0$ represents powers. The sparsity level was $s = 0.001$, leading to 1 non-zero elements in β. The results outside and inside parentheses were calculated from parametric bootstrap- and asymptotics-based methods, respectively.

c	0	1	2	3	4	5
iSPU(1)	4.9 (4.7)	5.7 (5.5)	4.5 (4.5)	4.9 (4.6)	6.2 (5.7)	5.9 (5.8)
iSPU(2)	2.7 (5.4)	2.3 (5.8)	6.3 (9.9)	11.6 (16.8)	20.6 (25.9)	26.9 (31.9)
iSPU(3)	5.8 (5.6)	6.8 (6.4)	15.6 (14.9)	31.7 (31.1)	42.7 (42.4)	52.4 (52.5)
iSPU(4)	3.1 (4.1)	5.9 (7.1)	23.4 (24.4)	42.6 (43.7)	54.4 (55.3)	61.4 (62.9)
iSPU(5)	5.8 (4.9)	9.1 (8.2)	29 (28.1)	47.6 (46.5)	59.8 (58.5)	67.6 (67)
iSPU(6)	3.8 (3.5)	8.9 (8)	30.8 (28.9)	51 (49.7)	60.1 (59.4)	69.7 (69)
iSPU(∞)	9 (7.6)	15.1 (13.1)	43.6 (40.8)	63.2 (61.8)	70.1 (69.3)	76.6 (75.9)
aiSPU	5.8 (6.3)	10 (10.6)	37.5 (38.6)	58.5 (58.3)	67.6 (68.1)	74.6 (74.8)

Table S5: Empirical Type I errors and power (in percentage) of various tests under $G \times E$ interactions with $p = 1000$ and $n = 200$. Zero signal strength $c = 0$ represents Type I errors, while $c \neq 0$ represents powers. The sparsity level was $s = 0.01$, leading to 10 non-zero elements in β. The results outside and inside parentheses were calculated from parametric bootstrap- and asymptotics-based methods, respectively.

c	0	1	2	3	4	5
iSPU(1)	4.8 (4.7)	4.1 (3.9)	5.1 (4.9)	5 (4.9)	5.7 (5.4)	5.7 (5.7)
iSPU(2)	2.6 (5.3)	11.7 (17.7)	40.9 (44.3)	62.7 (65.3)	73.1 (73.9)	78.5 (78.5)
iSPU(3)	5.9 (5.7)	9.2 (8.5)	28.8 (28)	44.9 (43)	50.3 (49.5)	55 (53.3)
iSPU(4)	3 (4)	25.4 (26.7)	82.5 (82.9)	95.1 (95.6)	98.2 (98.2)	99.1 (99.2)
iSPU(5)	5.9 (5)	19 (18.1)	64.2 (62)	79.3 (78.2)	84.6 (83.9)	86.9 (86.2)
iSPU(6)	3.7 (3.3)	30.1 (27.7)	89.3 (87.9)	97.5 (97.3)	98.9 (98.9)	99.6 (99.3)
iSPU(∞)	9 (7.5)	32.4 (28.7)	91.7 (89.1)	98.7 (98.3)	99.5 (99.5)	99.9 (99.9)
aiSPU	5.8 (6.2)	27.2 (29.7)	89.3 (89.4)	98.1 (98.3)	99.4 (99.4)	99.8 (99.8)

Table S6: Empirical Type I errors and power (in percentage) of various tests under $G \times E$ interactions with $p = 1000$ and $n = 200$. Zero signal strength $c = 0$ represents Type I errors, while $c \neq 0$ represents powers. The sparsity level was $s = 0.05$, leading to 50 non-zero elements in β. The results outside and inside parentheses were calculated from parametric bootstrap- and asymptotics-based methods, respectively.

c	0	1	2	3	4	5
iSPU(1)	5.8 (5.5)	4.8 (5.5)	6.2 (5.4)	6.1 (6)	7.3 (6.9)	6.9 (7.3)
iSPU(2)	2.3 (5.4)	45.3 (48.7)	75.4 (76.6)	84.8 (83.6)	86.2 (85.6)	86.9 (86.1)
iSPU(3)	5.4 (5.2)	11.4 (11.6)	17.8 (15.8)	19.8 (18.7)	20.3 (19.2)	21.8 (19.4)
iSPU(4)	2.7 (4.1)	56.7 (55.9)	88.5 (86.8)	93.7 (91.8)	95 (93.8)	95.4 (94.8)
iSPU(5)	6.1 (5)	25 (22.4)	37 (34.5)	40.6 (37.4)	43.6 (40.8)	45.5 (41.8)
iSPU(6)	4.1 (3.9)	53.7 (48.6)	83.7 (79.8)	90.8 (88.2)	91.5 (88.6)	92.3 (90.6)
iSPU(∞)	8.5 (7.3)	34.2 (27.5)	61.7 (52.1)	69.3 (59)	75 (63.4)	75.4 (64.1)
aiSPU	5.7 (6.5)	46.4 (46.9)	78.5 (78.9)	86.7 (86.2)	89.2 (88.2)	90.2 (89.8)

Table S7: Empirical Type I errors and power (in percentage) of various tests under high-dimensional linear models simulations. Zero signal strength $c = 0$ represents Type I errors, while $c \neq 0$ represents powers. Zero signal strength $c = 0$ represents Type I errors, while $c \neq 0$ represents powers. The sparsity level was $s = 0.2$, leading to 200 non-zero elements in β. The results outside and inside parentheses were calculated from parametric bootstrap- and asymptotics-based methods, respectively.

c	0	0.5	1	1.5	2
iSPU(1)	5.8 (5.5)	5.9 (5.1)	5.6 (5.5)	6.5 (6.1)	5.7 (5.9)
iSPU(2)	2.3 (5.4)	47.6 (51.6)	76.5 (75.5)	83 (81.1)	85.4 (85.3)
iSPU(3)	5.4 (5.2)	10.2 (9.1)	11.5 (10.5)	13.2 (11.5)	12.7 (10.8)
iSPU(4)	2.7 (4.1)	43.1 (42)	70.9 (67.2)	78.6 (71.7)	81 (77.1)
iSPU(5)	6.1 (5)	12.5 (11)	16.3 (14.4)	18.1 (15.7)	18.4 (16.5)
iSPU(6)	4.1 (3.9)	30.8 (25.2)	52.8 (43.1)	59.4 (52.6)	62.3 (53.8)
iSPU(Inf)	8.5 (7.4)	16.6 (11.7)	23.3 (15.5)	26.5 (19.4)	27.1 (19.4)
aiSPU	5.7 (6.5)	32.9 (34.4)	61.2 (58.6)	68.5 (65)	73.3 (71.4)

Table S8: Empirical Type I errors and power (in percentage) of various tests under $G \times E$ interactions with $p = 1000$ and $n = 200$. Zero signal strength $c = 0$ represents Type I errors, while $c \neq 0$ represents powers. The sparsity level was $s = 0.2$, leading to 200 non-zero elements in β. The informative variables in β was generated from a uniform distribution $U(0, c)$. The results outside and inside parentheses were calculated from parametric bootstrap- and asymptotics-based methods, respectively.

c	0	0.01	0.05	0.1	0.3	0.5
iSPU(1)	5.8 (5.5)	5.8 (5.7)	19.9 (19.2)	54.9 (53.1)	98.3 (98.4)	100 (99.9)
iSPU(2)	2.3 (5.4)	2.1 (5.8)	2.3 (7.6)	7.1 (12.7)	47.8 (52.8)	67.8 (70.2)
iSPU(3)	5.4 (5.2)	4.8 (5.1)	7.5 (7.9)	25.3 (23.8)	87.8 (86.2)	97.1 (96.5)
iSPU(4)	2.7 (4.2)	2.4 (3.1)	3.1 (4.3)	6.3 (7.5)	41.8 (39.1)	62.5 (56.3)
iSPU(5)	6.1 (5)	6.5 (5.4)	6.3 (5.4)	10 (8.9)	52.6 (48.3)	74.3 (70.1)
iSPU(6)	4.2 (4)	4.5 (4.1)	4.1 (3.6)	5.9 (5.2)	27.2 (22.2)	38.8 (32.4)
iSPU(∞)	8.5 (7.4)	9.2 (7.7)	10.5 (9.2)	10.2 (8.2)	21.2 (15.1)	21.9 (15.1)
aiSPU	5.7 (6.6)	6.2 (6.9)	13.8 (11.8)	34.3 (31.5)	96.3 (93.9)	99.3 (98.5)

Table S9: Empirical Type I errors and power (in percentage) of various tests under $G \times E$ interactions with $p = 1000$ and $n = 200$. Zero signal strength $c = 0$ represents Type I errors, while $c \neq 0$ represents powers. The informative variables in β was selected to be those with main effects and generated from a uniform distribution $U(-c, c)$. The results outside and inside parentheses were calculated from parametric bootstrap- and asymptotics-based methods, respectively.

c	0	1	2	3	4	5
iSPU(1)	4.9 (4.8)	5.6 (5.2)	6.4 (6)	6.1 (5.9)	6 (5.3)	6.1 (5.7)
iSPU(2)	2.7 (5.3)	6.9 (10.3)	19.4 (24.8)	37 (42)	51.2 (53.4)	58.5 (62)
iSPU(3)	5.9 (5.6)	6.3 (6.1)	27.3 (26.6)	50.4 (50.1)	62.3 (61.5)	69.7 (68.2)
iSPU(4)	3.1 (4)	12.4 (14.3)	58.1 (59.3)	85 (85.8)	94.5 (93.9)	97.2 (97.2)
iSPU(5)	5.9 (5)	13.6 (12.5)	58.7 (57.1)	80.7 (79.7)	88.6 (87.1)	92.6 (92.3)
iSPU(6)	3.8 (3.3)	18 (16.6)	70.6 (68.5)	91.8 (91.5)	96.9 (96.3)	98.6 (98.2)
iSPU(Inf)	9 (7.6)	24.1 (21.5)	82.6 (80.5)	96.5 (95.5)	98.7 (98.4)	99.7 (99.4)
aiSPU	5.8 (6.3)	17.3 (19.7)	76.2 (77.1)	94.5 (94.7)	98.4 (98)	99.1 (99.3)

Table S10: Empirical Type I errors and power (in percentage) of various tests under high-dimensional linear models simulations. Zero signal strength $c = 0$ represents Type I errors, while $c \neq 0$ represents powers. The sparsity level was $s = 0.001$, leading to 1 non-zero element in β. The results outside and inside parentheses were calculated from parametric bootstrap- and asymptotics-based methods, respectively.

c	0	0.3	0.5	0.7	1
iSPU(1)	5.6 (5.4)	6.7 (6.1)	6.6 (6.3)	7.5 (7.2)	8.9 (8.6)
iSPU(2)	3.6 (3.3)	4.2 (5.7)	6.6 (8.2)	15.3 (18.9)	32.2 (38.7)
iSPU(3)	5 (4.8)	6.4 (5.6)	14.6 (13.5)	41.7 (40.1)	64.2 (63.1)
iSPU(4)	3.8 (1.8)	9.1 (7.5)	29.5 (26.4)	54.6 (52.1)	71.3 (71.1)
iSPU(5)	5.5 (3.5)	16.5 (12.8)	36.1 (32.7)	57.7 (54.5)	72.1 (70.6)
iSPU(6)	4.9 (2.2)	18.2 (13.3)	38.8 (33.8)	61.9 (58.2)	73.7 (71.9)
iSPU(∞)	3.5 (4.6)	16.1 (18.3)	36.5 (38.7)	61.4 (61.9)	74.1 (74.5)
aiSPU	5.3 (4.1)	16.6 (16.5)	38.5 (38.3)	61.4 (60.1)	73.7 (73.7)

Table S11: Empirical Type I errors and power (in percentage) of various tests under high-dimensional linear models simulations. Zero signal strength $c = 0$ represents Type I errors, while $c \neq 0$ represents powers. The sparsity level was $s = 0.01$, leading to 10 non-zero elements in β. The results outside and inside parentheses were calculated from parametric bootstrap- and asymptotics-based methods, respectively.

c	0	0.1	0.2	0.3	0.4	0.5
iSPU(1)	5.2 (5.5)	7.1 (6)	6 (5.3)	7.7 (8.4)	9 (8.4)	8.7 (7.3)
iSPU(2)	4.1 (4.3)	4 (6.2)	10.4 (13.3)	24.4 (29.8)	42.6 (49.4)	52.9 (64.7)
iSPU(3)	5.1 (4.6)	5.7 (4.5)	10.2 (9)	21.2 (18.5)	35.7 (33.6)	47.3 (44)
iSPU(4)	5.6 (2.1)	5.9 (3.9)	19.5 (16.2)	55.3 (52.4)	84.4 (83.2)	95.3 (95.8)
iSPU(5)	5.2 (3.3)	5.6 (4)	18.3 (13)	40.8 (36.8)	68.4 (64.1)	81.4 (79.8)
iSPU(6)	5.9 (2.3)	6.6 (3.4)	24.7 (16.5)	67.9 (60.5)	93.9 (90)	98.8 (98.1)
iSPU(∞)	3.5 (4.6)	4.5 (5.2)	12.7 (16.2)	48.5 (52.8)	81.1 (83.6)	94.1 (96)
aiSPU	5.2 (4.5)	6.6 (5.4)	17.7 (16.9)	58.3 (55.7)	88.1 (86.7)	96.2 (96.5)

Table S12: Empirical Type I errors and power (in percentage) of various tests under high-dimensional linear models simulations. Zero signal strength $c = 0$ represents Type I errors, while $c \neq 0$ represents powers. The sparsity level was $s = 0.2$, leading to 200 non-zero elements in β. The results outside and inside parentheses were calculated from parametric bootstrap- and asymptotics-based methods, respectively.

c	0	0.05	0.07	0.1	0.2	0.3
iSPU(1)	6.3 (6)	5.5 (6.1)	7.4 (5.7)	3.4 (4)	5.3 (4.3)	6.4 (6.4)
iSPU(2)	2.8 (3.5)	13.9 (18.6)	23.3 (29.5)	42.7 (51.7)	85 (87.9)	95.7 (96.4)
iSPU(3)	5.4 (5.4)	6.1 (4.9)	6.7 (6.2)	8.4 (7.2)	12.1 (10.1)	15.7 (13.6)
iSPU(4)	3.7 (1.7)	12.4 (9.1)	22.8 (19.3)	41.7 (40.2)	84.5 (82.1)	95 (92.9)
iSPU(5)	6.1 (3.9)	7 (4)	6.7 (3.5)	8.7 (4.7)	23.2 (16.4)	27.1 (18.6)
iSPU(6)	4.7 (2)	9.9 (4.6)	14.9 (9.2)	33 (23.4)	77.8 (67.1)	85.7 (77.1)
iSPU(∞)	2.8 (4.3)	4.6 (5.5)	5.2 (6.4)	5.9 (7.8)	18.8 (20.8)	29.3 (27.9)
aiSPU	4.7 (3.7)	8.9 (8.9)	15.3 (15.3)	27.7 (33)	73.9 (79.2)	87.1 (89.3)

Table S13: Empirical Type I errors and power (in percentage) of various tests under high-dimensional linear models simulations. Zero signal strength $c = 0$ represents Type I errors, while $c \neq 0$ represents powers. The sparsity level was $s = 0.2$, leading to 200 non-zero elements in β. We generated informative variables in β from a uniform distribution $U(0, c)$. The results outside and inside parentheses were calculated from parametric bootstrap- and asymptotics-based methods, respectively.

c	0	0.01	0.02	0.03	0.04	0.05
iSPU(1)	6.2 (6.1)	24.6 (24)	59.6 (58.2)	86.2 (85.9)	96.1 (95.8)	98.7 (98.7)
iSPU(2)	3 (3.3)	4.5 (6.8)	18 (21.5)	40.6 (47)	65.8 (74.7)	82.2 (88.1)
iSPU(3)	5.2 (5.2)	19.3 (16.7)	51.7 (48.8)	80.4 (78.8)	93.8 (92.6)	98.7 (98)
iSPU(4)	3.6 (1.5)	5.6 (3.2)	16.6 (11.3)	38.2 (33.9)	59.8 (58.9)	78.2 (78.9)
iSPU(5)	5.5 (3.4)	12.1 (7.3)	32.1 (22.8)	60.1 (50.9)	80.4 (74.7)	92.4 (86.8)
iSPU(6)	4.4 (1.8)	7 (2.6)	13.3 (7.7)	26.7 (18.2)	46.7 (32.1)	63 (50.5)
iSPU(∞)	3.2 (4.1)	4.5 (6)	5.2 (7)	7.6 (8.8)	8.9 (11)	10.2 (13.9)
aiSPU	5.1 (3.9)	13.4 (11.4)	46.3 (41.8)	80.9 (77.4)	93.8 (92.9)	97.7 (97)

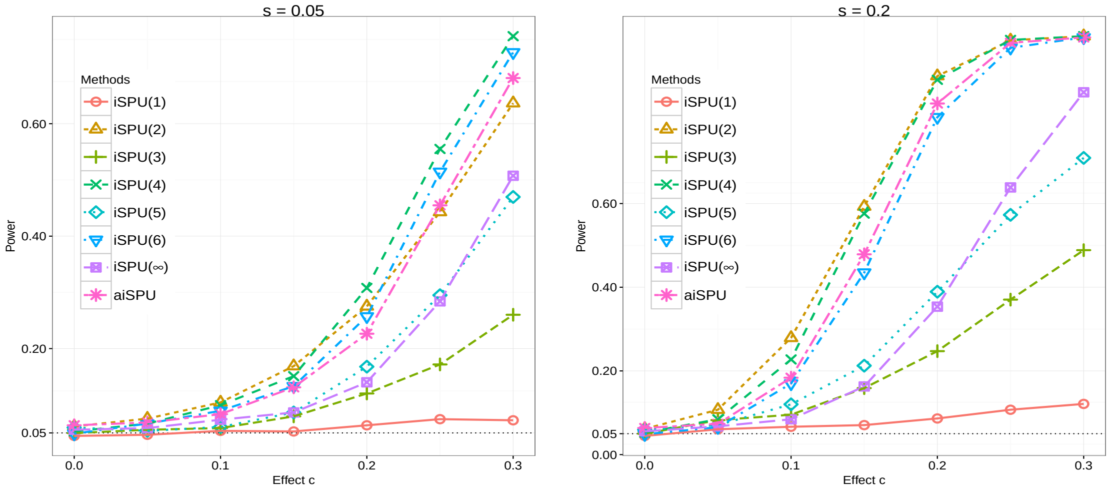

Figure S1: Power comparison for different methods under $G \times E$ interaction simulations with $n = 2000$, $p = 300$, and $q_1 = q_2 = 20$. n, p, q_1, and q_2 stand for the sample size, number of terms in $G \times E$ interaction, number of the positive genetic main effects, and number of the negative genetic main effects, respectively. All tests were based on TLP. We varied the sparsity level s.

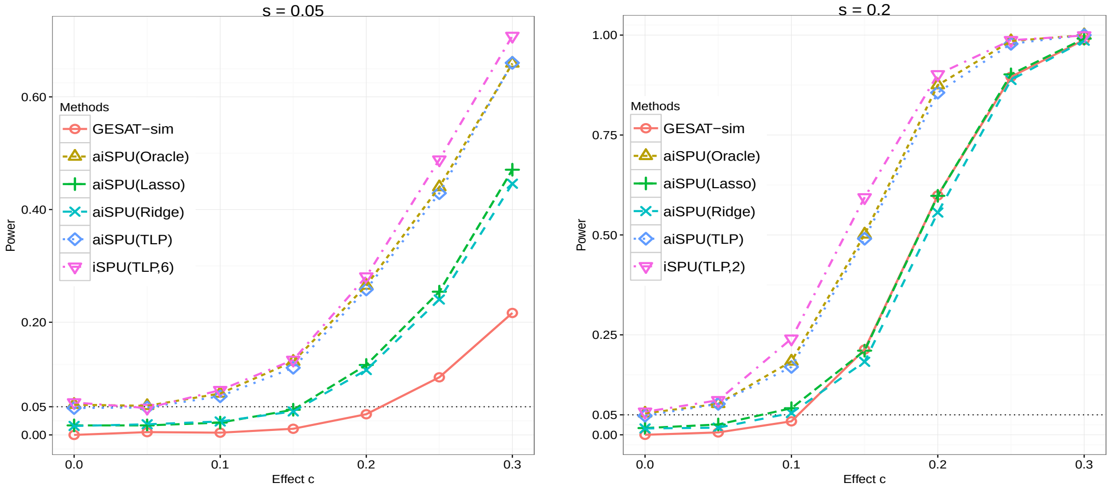

Figure S2: Power comparison for different methods under $G \times E$ interaction simulations with $n = 2000$, $p = 300$, and $q_1 = q_2 = 20$. n, p, q_1, and q_2 stand for the sample size, number of terms in $G \times E$ interaction, number of the positive genetic main effects, and number of the negative genetic main effects, respectively. The SNPs were correlated ($\rho = 0.3$). We varied the sparsity level s.

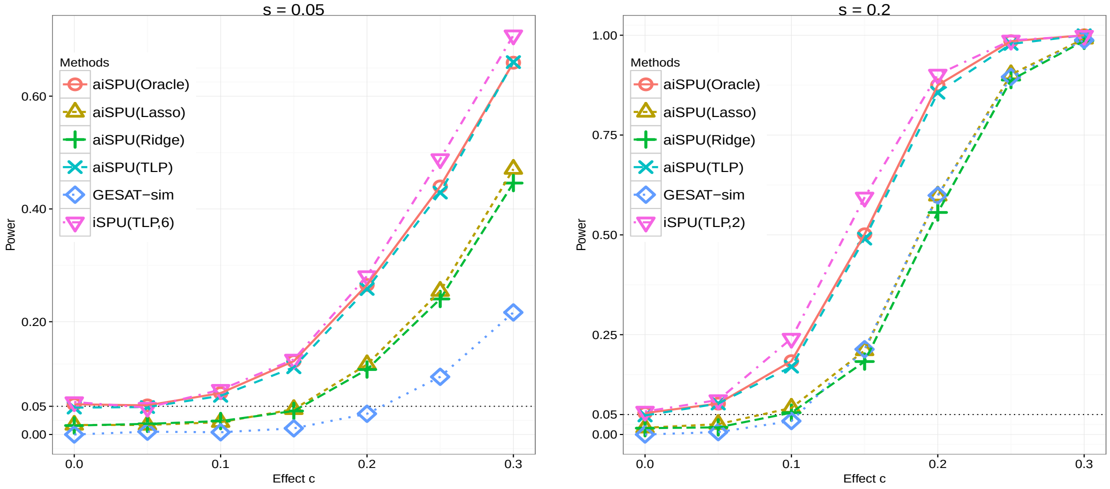

Figure S3: Power comparison for different methods under $G \times E$ interaction simulation with $n = 2000$, $p = 300$, and $q_1 = q_2 = 50$. n, p, q_1, and q_2 stand for the sample size, number of terms in $G \times E$ interaction, number of the positive genetic main effects, and number of the negative genetic main effects, respectively. The SNPs were correlated ($\rho = 0.3$). We varied the sparsity level s.

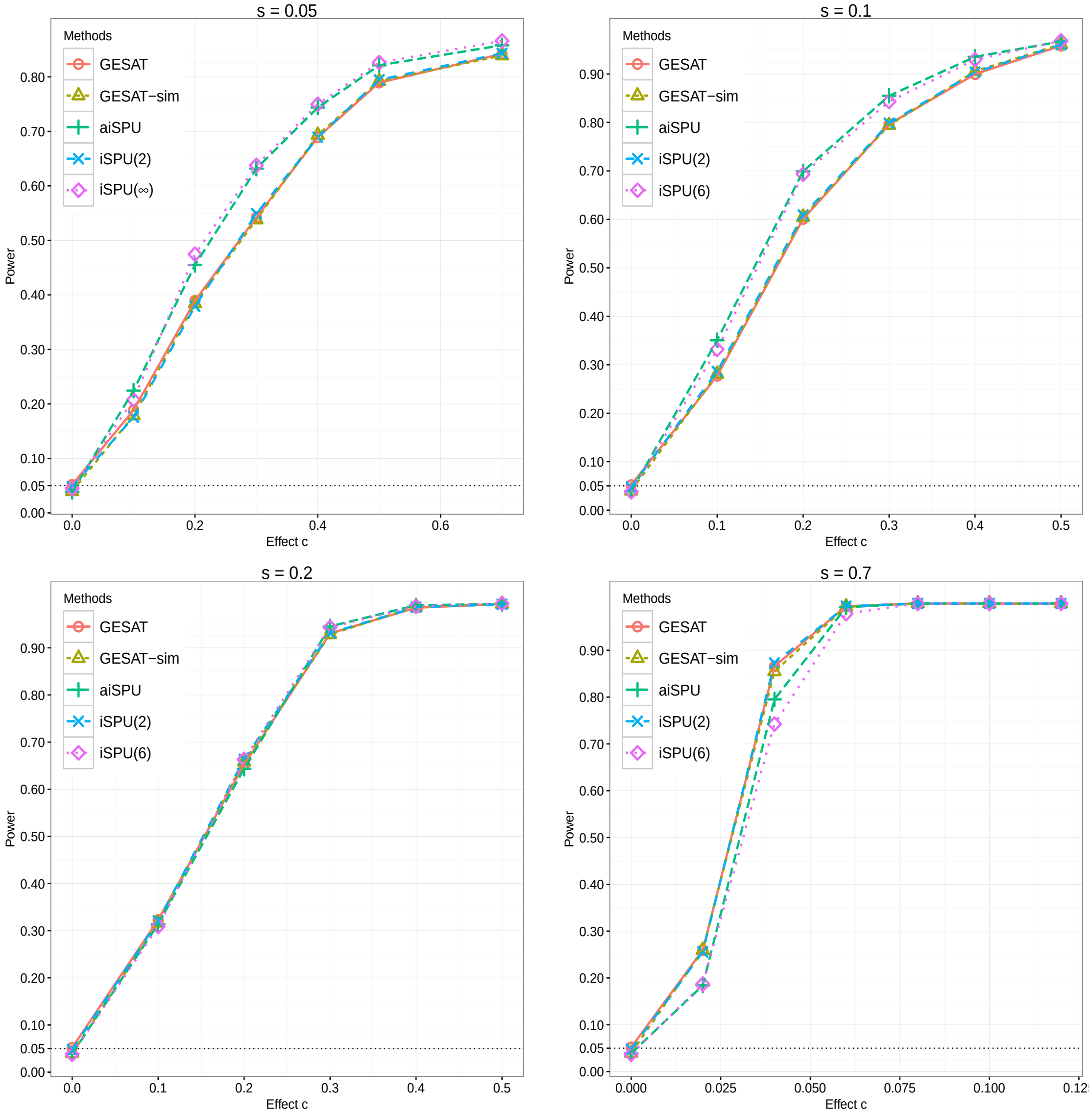

Figure S4: Power comparison for different methods under $G \times E$ interaction simulations with $n = 2000$, $q_1 = 2$, $q_2 = 0$, and $p = 25$. n, p, q_1, and q_2 stand for the sample size, number of coefficients in $G \times E$ interaction effects, number of positive coefficients in main genetics effects, and number of negative coefficients in main genetics effects, respectively. We varied the sparsity level s.

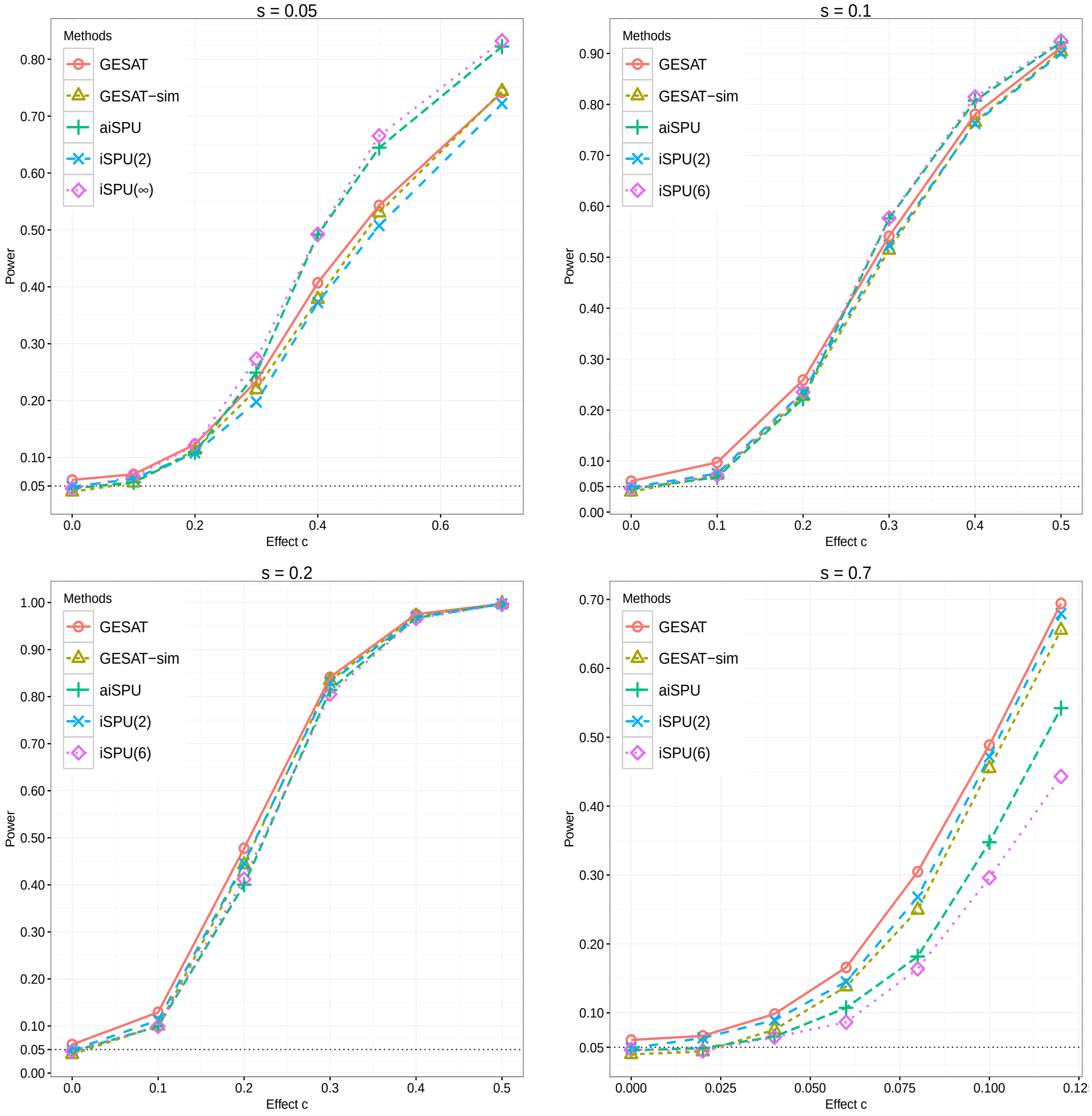

Figure S5: Power comparison for different methods under $G \times E$ interaction simulations with $n = 2000$, $q_1 = 2$, $q_2 = 0$, and $p = 50$. n, p, q_1, and q_2 stand for the sample size, number of coefficients in $G \times E$ interaction effects, number of positive coefficients in main genetics effects, and number of negative coefficients in main genetics effects, respectively. We varied the sparsity level s.

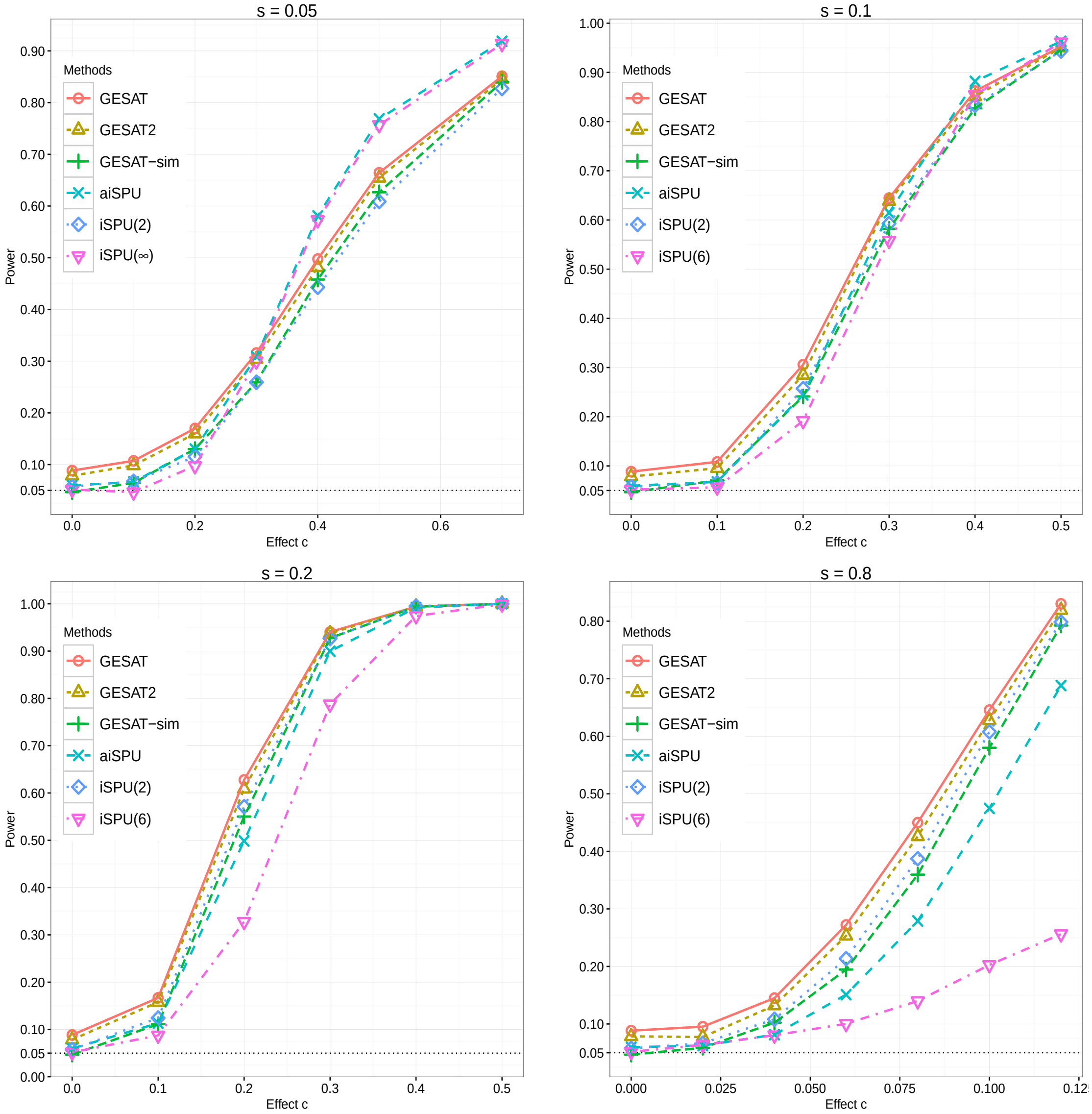

Figure S6: Power comparison for different methods under $G \times E$ interaction simulations with $n = 2000$, $q_1 = 2$, $q_2 = 0$, and $p = 75$. n, p, q_1, and q_2 stand for the sample size, number of coefficients in $G \times E$ interaction effects, number of positive coefficients in main genetics effects, and number of negative coefficients in main genetics effects, respectively. GESAT2 stands for the GESAT with much a larger searching region (from 1×10^{-6} to 44.7 (i.e. $\sqrt{n}$)) for tuning parameter λ. We varied the sparsity level s.

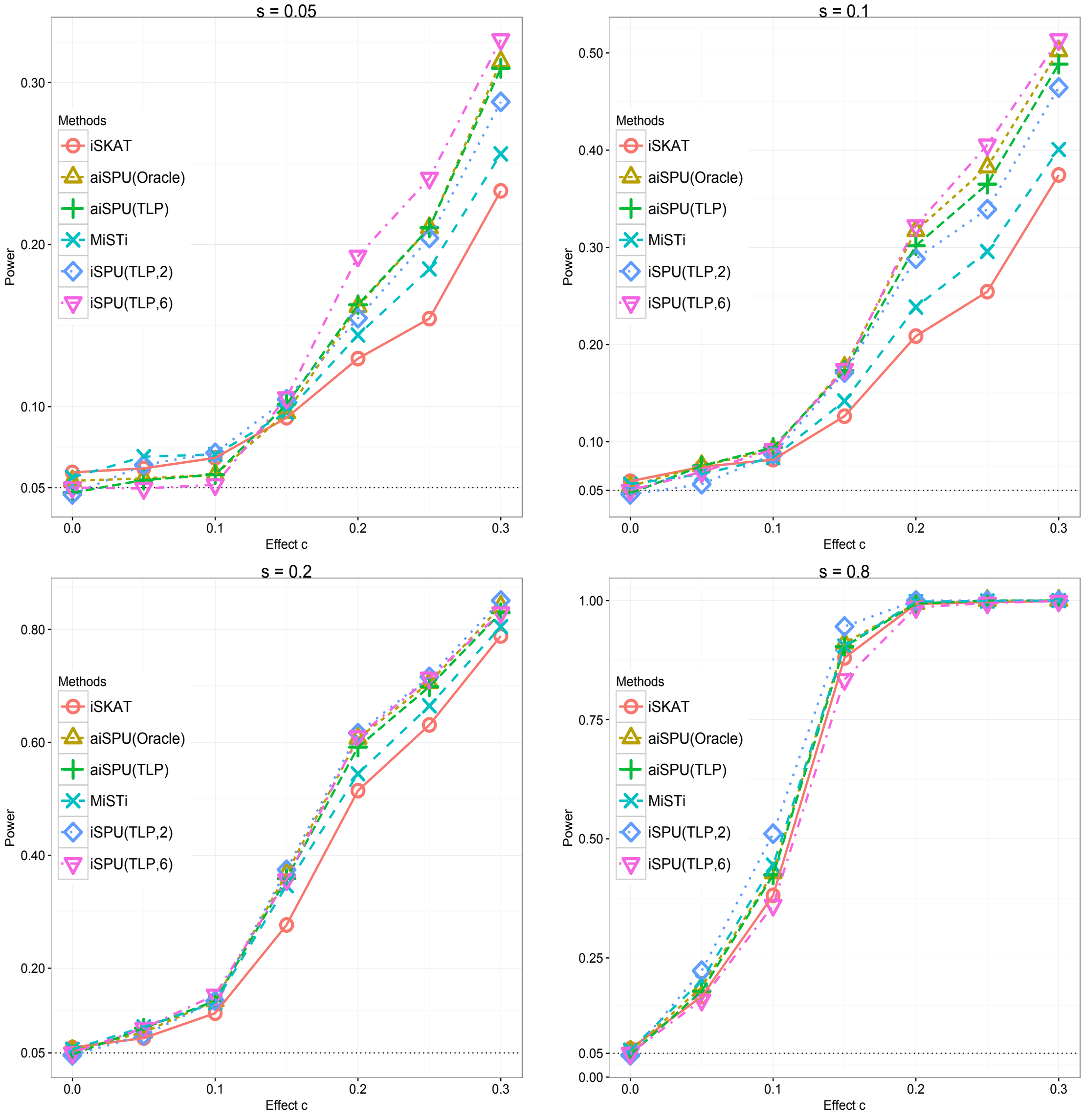

Figure S7: Power comparison for different methods in rare variants simulations with $n = 2000$, $q_1 = 2$, $q_2 = 0$, and $p = 25$. n, p, q_1, and q_2 stand for the sample size, number of terms in $G \times E$ interaction, number of the positive genetic main effects, and number of the negative genetic main effects, respectively. SNPs were generated with MAFs ranging from 0.005 to 0.05. We varied the sparsity level s.

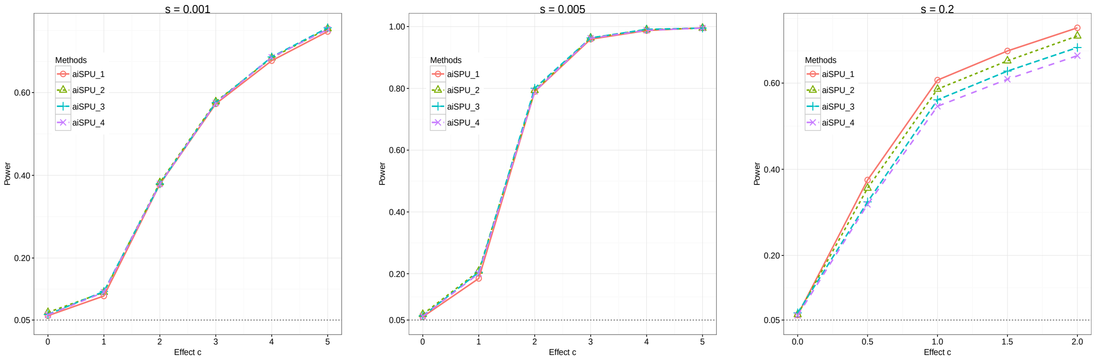

Figure S8: Empirical power of aiSPU with different Γ sets for $G \times E$ interactions with $n = 200$, $p = 1000$. aiSPU_1, aiSPU_2, aiSPU_3, aiSPU_4 represent aiSPU with $\Gamma_1 = \{1, 2, 3, 4; \infty\}$, $\Gamma_2 = \{1, 2, \ldots, 6, \infty\}$, $\Gamma_3 = \{1, \ldots, 8, \infty\}$, and $\Gamma_4 = \{1, 2, \ldots, 10, \infty\}$, respectively. We varied the sparsity level s.

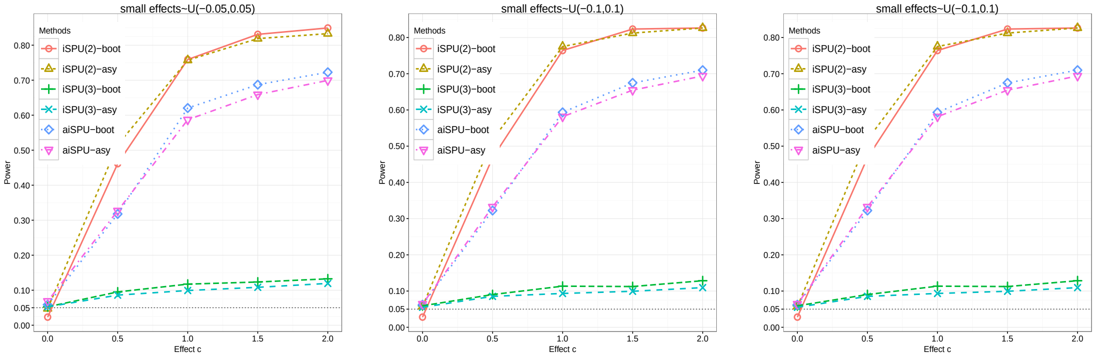

Figure S9: Empirical power of aiSPU under $G \times E$ interaction with $n = 200$, $p = 1000$, and sparsity level $s = 0.2$. We randomly selected 100 variables in $\mathbb{Z}$ and set the effect size followed a uniform distribution. -boot and -asy stand for the results based on bootstrap and asymptotics, respectively.

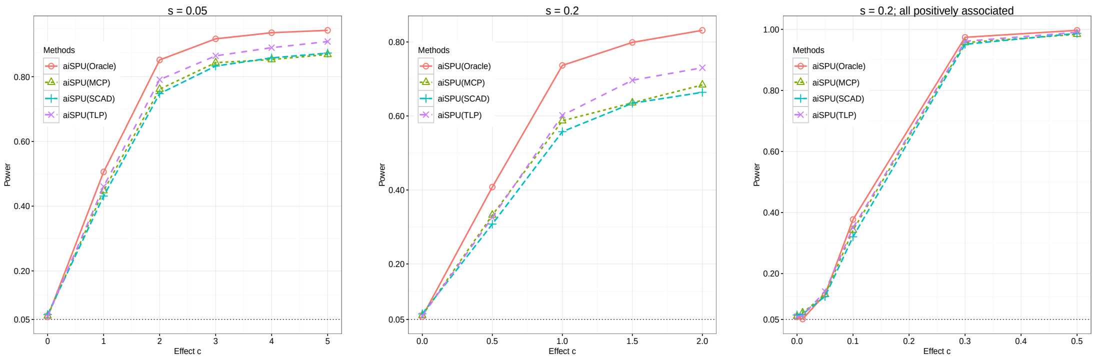

Figure S10: Empirical power of aiSPU with different non-convex penalities under $G \times E$ interaction with $n = 200$, $p = 1000$, and varied sparsity level s. For a fair comparison, all the results were based on the parametric bootstrap.

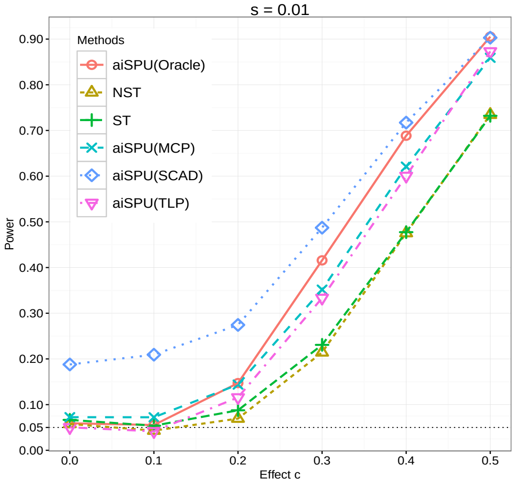

Figure S11: Empirical power of aiSPU with different non-convex penalities under high-dimensional linear models with $n = 200$, $p = 1000$, and sparsity level $s = 0.01$. For a fair comparison, all the aiSPU results were based on the parametric bootstrap.

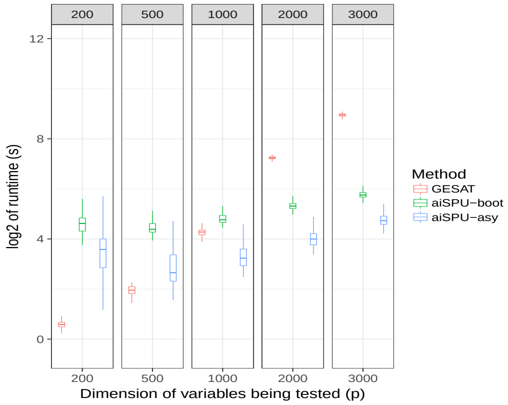

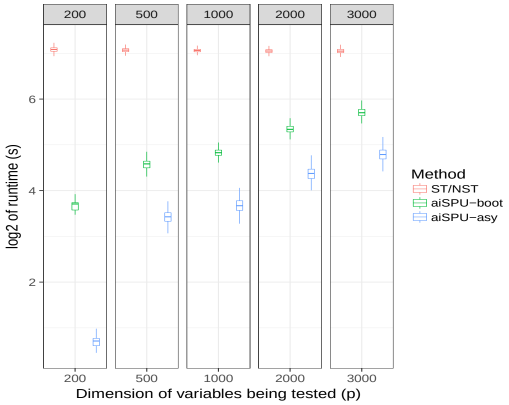

Figure S12: Computational time comparison under both $G \times E$ interaction models (left panel) and linear models (right panel) with $n = 200$, sparsity level $s = 0$. In Zhang and Cheng (2017), ST and NST have been calculated simultaneously; ST/NST stands for the runtime for ST plus NST. We varied the number of variables being tested, p.

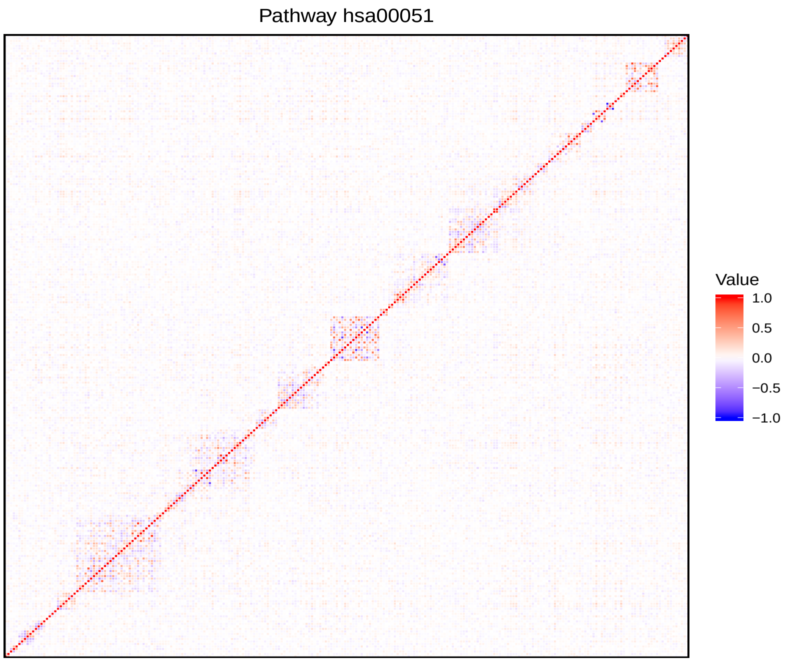

Figure S13: The correlation heatmap for SNPs used in pathway hsa00051. Pathway hsa00051 is the significant pathway identified by aiSPU.
